# Nanopipette dynamic microscopy unveils nano coffee ring

**DOI:** 10.1073/pnas.2314320121

**Published:** 2024-07-02

**Authors:** Deyi Zhang, Yi Shao, Jiayi Zhou, Qiangwei Zhan, Ziyang Wen, Sheng Mao, Jingjing Wei, Limin Qi, Yuanhua Shao, Huan Wang

**Affiliations:** ^a^Beijing National Laboratory for Molecular Sciences, College of Chemistry and Molecular Engineering, Key Laboratory of Polymer Chemistry and Physics, National Biomedical Imaging Center, Peking University, Beijing 100871, People’s Republic of China; ^b^College of Engineering, Peking University, Beijing 100871, People’s Republic of China; ^c^School of Chemistry and Chemical Engineering, Shandong University, Jinan 250100, People’s Republic of China

**Keywords:** liquid-phase electron microscopy, nanopipette, coffee-ring effect, directed assembly

## Abstract

Liquid-phase electron microscopy (LP-EM) imaging has revolutionized our understanding of nanosynthesis and assembly. However, the current closed geometry limits its application for open systems. The ubiquitous physical process of the coffee-ring phenomenon that underpins materials and engineering science remains elusive at the nanoscale due to the lack of experimental tools. We introduce a quartz nanopipette liquid cell with a tunable dimension that requires only standard microscopes. Depending on the imaging condition, the open geometry of the nanopipette allows the imaging of evaporation-induced pattern formation, but it can also function as an ordinary closed-geometry liquid cell where evaporation is negligible despite the nano opening. The nano coffee-ring phenomenon was observed by tracking individual nanoparticles in an evaporating nanodroplet created from a thin liquid film by interfacial instability. Nanoflows drive the assembly and disruption of a ring pattern with the absence of particle–particle correlations. With surface effects, nanoflows override thermal fluctuations at tens of nanometers, in which nanoparticles displayed a “drunken man trajectory” and performed work at a value much smaller than *k*_B_*T*.

## Significance

We report a nanopipette liquid cell for in situ electron microscopy imaging that allows direct imaging of both open and closed systems, such that the powerful but high-entry level liquid-phase electron microscopy (LP-EM) imaging requires sophisticated operators to become a routine method for ordinary users. Direct imaging elucidated the dominating role of capillary flow at dozens of nanometers in evaporating nanodroplets. Rich contact line dynamics in the formation of the nano coffee ring highlighted the interplay of nanoflows and surface forces in directing pattern formation against thermal fluctuations.

The coffee-ring effect (1–3) is a widely observed phenomenon in which a droplet with suspended particles forms a ring-like deposit upon evaporation on a solid surface due to the pinned contact lines and outward capillary flow. This effect has led to advances in many fields of science and engineering (4), including the fabrication of solution-processed solar cells (5), self-assembly (6), nanoparticle separation (7), and disease detection (8). Simulations showed coffee rings of tens of nanometers (9–11); however, experiments suggested a lower limit of ~10 μm (12). The interplay of capillarity (13), surface forces (14–16), interfacial stability (17), and nanofluidics (18) remains elusive at nanoscales, which can stimulate peculiar nonequilibrium dynamics that differ from the microscale (16, 17, 19, 20).

In situ imaging by liquid-phase electron microscopy (LP-EM) and single-particle analysis has revolutionized the understanding of nanoparticles (21–27), soft materials (28, 29), and their assembly (30–36). The mainstream closed-geometry liquid cells, including silicon nitride (SiN) and graphene liquid cells (GLC), are techniques that demand sophisticated operators. They are usually incompatible with the evaporation process and suffer from shape deformation when gas forms inside the liquid cell. Here, we report a nanopipette liquid cell with a sealable opening that dissipates extra pressure yet remains liquid-filled during evaporation and allows one to conduct LP-EM experiments with a limited budget.

## Results

### Fabrication of Nanopipettes for LP-EM Imaging.

**Fig. 1.**
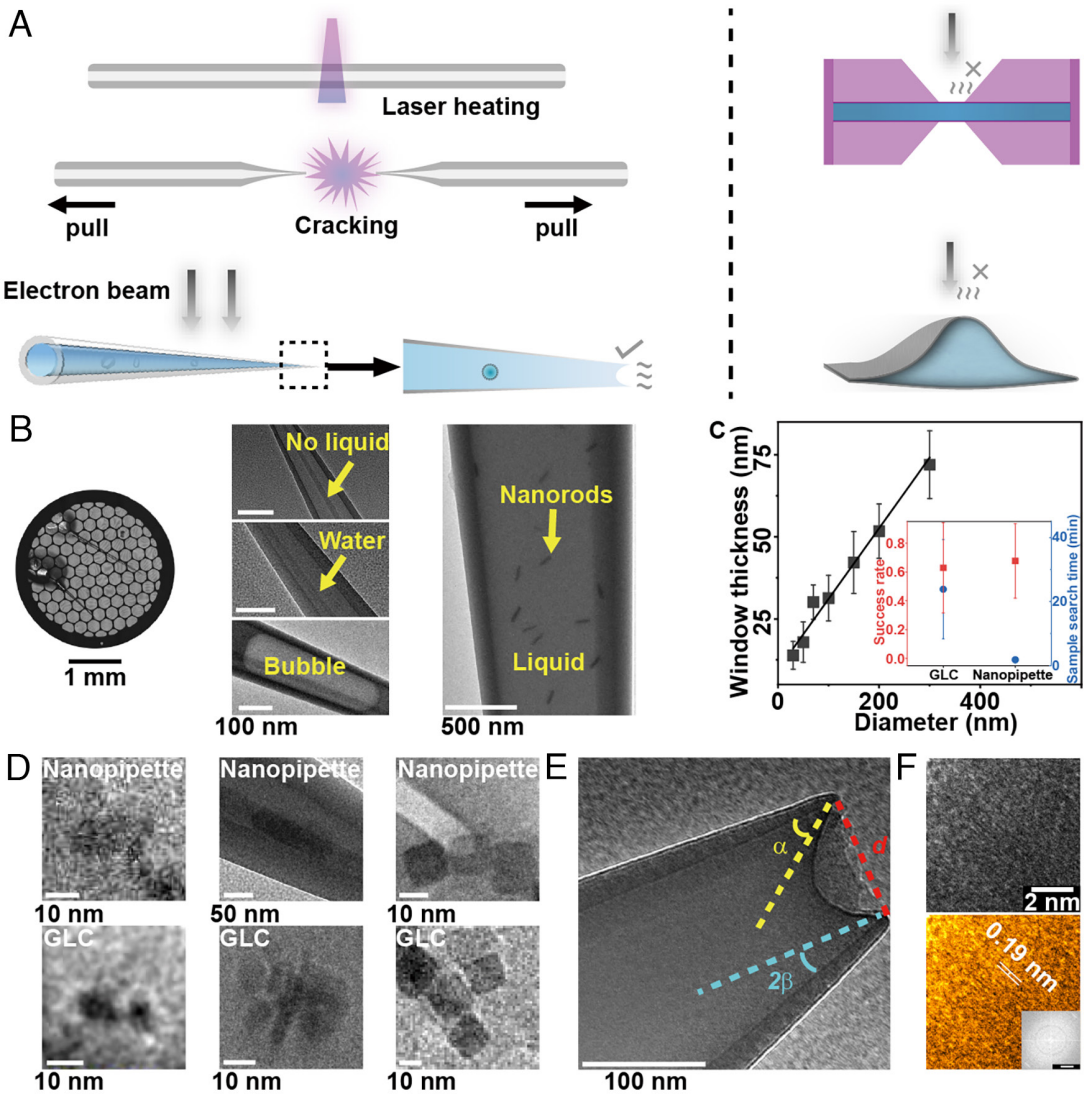
Quartz nanopipette for LP-EM. (*A*) Schematic diagram of a quartz nanopipette (*Left*), a SiN liquid cell (*Right*-*Top*), and a GLC (*Right*-*Bottom*). (*B*) The bright-field optical image (*Left*) and EM images (*Right*) of nanopipettes on a standard EM grid. (*C*) A plot of window (wall) thickness as a function of diameter for a nanopipette. *Inset*: comparison of nanopipettes’ success rate (*Left* axis) and mean sample search time (*Right* axis) with GLCs. (*D*) Comparisons of images obtained from nanopipette and GLC. Sample solutions: single-stranded DNA (*Left*, *Bottom* panel adapted from ref. 31), 100 mM lipoate in H_2_O (*Middle*, *Bottom* panel adapted from ref. 36), Fe_3_O_4_ nanocube in *o*DCB (*Right*). (*E*) Liquid surface curvature at the tip of the nanopipette, and the definition of *α*, *β* and *d*. (*F*) A HR image and corresponding pseudocolor image of a GNR were obtained from a nanopipette showing a lattice structure. Zoomed (Scale bar: 2 1/nm) shows corresponding Fast Fourier Transformation (FFT) analysis. Imaging condition and statistics in *SI Appendix*, *Text*.

We fabricated nanopipettes made of quartz (37) (*SI Appendix*, *Text* and Fig. 1*A*), a material commonly used for macroscale liquid cells such as Ultraviolet–Visible spectroscopy, fluorescence, and Raman spectroscopy. The fabrication of nanopipettes with heating and pulling is a well-established process (37) since nanopipettes are a type of ultramicroelectrodes that have been widely used for the miniaturization of liquid–liquid interfaces (38), for chemical and biosensing (39–41), as well as for imaging in scanning electrochemical microscopy (42) and scanning ion conductance microscopy (43–45). Nanopipettes have not been used as liquid cells for LP-EM as their nanosized opening were thought to be incompatible with vacuum environments required for EM. However, open-ended carbon nanotubes can retain liquids under EM (46). To test the feasibility, quartz capillaries with a circular cross-section were stretched by laser-assisted heating to create tapered nanopipettes (*SI Appendix*, Fig. S1). The sample solution was loaded from the wide-open end of the nanopipette, where it flows spontaneously to the tip with the aid of filament by capillary action. The wide-open end (0.5 to 1 mm) was cut by microscissors with vacuum grease to seal it and allow it to stick on the grid. Multiple nanopipettes can be loaded onto a single grid (Fig. 1*B*), which can be easily identified under EM within seconds (*SI Appendix*, Fig. S1*C*). The image contrast was sufficient to distinguish between dry, liquid-filled, and bubble-containing nanopipettes, the filament, and gold nanorods (GNR, length 80 to 100 nm, width 24 to 50 nm) (Fig. 1*B* and *SI Appendix*, Fig. S2*A*).

The tip diameter ranges from 20 to 400 nm, depending on the parameters (*SI Appendix*, Fig. S3 and Table S1). Since the cross-section of the nanopipette is circular, the volume of liquid contained is measurable. The wall thickness linearly increases with the diameter (Fig. 1*C*), with respective measurements of 52, 42, 31, and 18 nm for diameters 200, 150, 100, and 50 nm. These values are comparable to the standard SiN liquid cells, whose typical thickness is 20 to 50 nm and liquid height is 100 to 200 nm. GNRs were observed to experience diffusion (*SI Appendix*, Fig. S4 and Movie S1). Thinner wall thickness enables higher-resolution imaging, especially for poor electron scattering materials. Biological samples such as ssDNA (diameter: 5 to 20 nm), micelles (diameter: 20 to 50 nm), and inorganic samples like Fe_3_O_4_ nanocubes (square side length: 10 to 20 nm) were encapsulated and imaged at 200 kV, 1 to 50 e^−^/(Å^2^ s) (Fig. 1*D*). The signal-to-noise ratio (SNR) determined from the pixel intensity was 4 to 7 for nanopipettes and 5 to 8 for GLC [the state-of-art high-resolution (HR) liquid cell] (*SI Appendix*, Fig. S5). The SNR of the nanopipette increases as its diameter decreases and remains ~8 at 800 nm for GNRs (*SI Appendix*, Fig. S6). Sample search time in the microscope was reduced for nanopipettes compared to GLC, from ~20 min to less than 1 min (*SI Appendix*, *Text* and Fig. 1 *C*, *Inset*), and liquid cell fabrication time was reduced for nanopipettes compared to GLC, from ~5 to 7 h (47) to 30 min (*SI Appendix*, *Text*), while maintaining a success rate of ~65% for finding high-quality liquid cells (*SI Appendix*, Fig. S7). The open geometry of nanopipettes (Fig. 1*E*) facilitates gas dissipation (*SI Appendix*, Fig. S8). Experiments (48) and simulations (49) have suggested that major radiolytic products for aqueous solutions are gas. HR images of GNRs showing lattice structure (Lattice stripe spacing *d* = 0.19 nm or 0.22 nm) were obtained at positions where the wall was <50 nm (Fig. 1*F* and *SI Appendix*, Fig. S9).

### The Kelvin and Hertz–Knudsen Equations Predict the Liquid Retention Time in the Nanopipettes.

Under standard imaging conditions [200 kV, 1 to 50 e^−^/(Å^2^ s)], nanopipettes maintained a liquid-filled state for 10 to 60 min. The Kelvin equation, applicable at the nanoscale (20), describes the change in vapor pressure *p* at a curved liquid–vapor interface. *p* at such a concave curved surface is smaller than the vapor pressure at a flat interface *p*_0_. The pressure difference is a function of the radius *r* and the angle *α*, as defined (Fig. 1*E*). Combining with the Hertz–Knudsen equation and the known liquid volume, we estimated the liquid retention time in the nanopipette (*SI Appendix*, *Text*). Depending on their vapor pressure and boiling point, at different tip diameters (*d*), the number ranges from 8 × 10^3^ to 10 s for water (*SI Appendix*, Table S2), 10^4^ to 10^2^ s for the organic solvent, *o*-dichlorobenzene (*o*DCB) (*SI Appendix*, Table S3), and 10^9^ to 10^6^ s for oleylamine (*SI Appendix*, Table S4). Experimental values are consistent with these estimates (*SI Appendix*, Fig. S10).

### Nanoparticle and Soft Material Dynamics in Nanopipettes.

The nanopipettes are readily imaged with standard EM grids and microscopes, requiring no extra specialized holder. They are highly reproducible in dimension. Due to the thin wall and liquid thickness, the spatial resolution for liquid samples in the nanopipette is higher than that of environmental transmission electron microscopy, which also differs in the geometry and instrumentation (50, 51) (Fig. 1*F*). At a low electron dose rate, the nanopipette emulates the conditions of closed-geometry liquid cells according to the estimation and experiments (*SI Appendix*, Fig. S10 and Tables S2–S4). One can use a localized electron beam to selectively trigger the deformation of the tip to seal it (*SI Appendix*, Fig. S11 and *Text*) (52–55). At a high electron dose rate, solvent evaporation dominates, and nanopipettes allow imaging of nanoflows and out-of-equilibrium processes induced by solvent drying.

Interestingly, the appearance of bubbles—the primary concern for disturbing dynamics in liquid cell electron microscopy experiments (49, 56)—was suppressed in the nanopipettes compared to GLC. It has been reported that the formation of radiolytic bubbles (57) is retarded in GLC than in SiN. A reservoir from the large-diameter end replenishes liquids as gases are released from the tip, similar to using a flow system in SiN experiments, which can decrease the radical species in the solution by shifting the radiolysis chemical equilibrium (49). Although pure quartz is more conductive under electron beam irradiation than under ordinary direct current field (58), coating a thin layer (~5 nm) of conductive carbon is beneficial without significantly reducing the contrast (*SI Appendix*, Fig. S12).

We observed similar dynamics of nanoparticle assembly, etching, nucleation, growth, coalescence, and ssDNA diffusion in the nanopipettes as in SiN and GLC (*SI Appendix*, *Text*). GNRs interacted to form tip-to-tip assembly in the aqueous solution (*SI Appendix*, Fig. S2 and Movie S2) (59). Electron-induced etching of 10 nm Au nanoparticles was observed (*SI Appendix*, Fig. S13 and Movie S3) (60). Two pathways, monomer attachment (Movie S4) and coalescence (Movie S5), were identified from the Pt nanoparticles’ nucleation and growth process (*SI Appendix*, Figs. S14–S16) (27, 61). The growth of a GNR into a gold nano arrow in the presence of cetyltrimethylammonium bromide was observed (*SI Appendix*, Fig. S17 and Movie S6) (62–65). For soft materials, the dynamics and degradation of ssDNA in D_2_O were captured at a dose rate of 8.4 e^−^/(Å^2^ s) (*SI Appendix*, Fig. S18 and Movie S7), lower than the reported damaging threshold [110 e^−^/(Å^2^ s)] (66, 67). In all systems, consistent with previous reports on nanoparticles (68) and micelles in SiN liquid cells (69), synthetic macromolecules (16), and DNA molecules (31) in the GLCs, as well as macromolecules imaged with optical microscopy (70), we observed the characteristic surface-mediated long hops of single particle trajectories. The quartz surface can be modified with well-defined silane chemistry (*SI Appendix*, *Text*); using it as the liquid cell material is advantageous for LP-EM.

### The Formation of a Nano Coffee Ring.

To observe the nano coffee ring phenomenon in the *o*DCB solution containing Pt(acetylacetonate)_2_ and oleylamine, we explore the effect of experimental conditions (*SI Appendix*, *Text*). Independent experiments under different conditions (117 nanopipettes, *SI Appendix*, Table S5) suggest that a high-contrast (HC) TEM (*SI Appendix*, Figs. S19 and S20), a nanopipette with a clogged tip (*SI Appendix*, Figs. S11 and S20–S22), and an electron dose rate of <100 e^−^/(Å^2^ s) (*SI Appendix*, Fig. S23) are unfavorable for observing solvent evaporation and the generation of nanodroplets (*SI Appendix*, Figs. S19–S24). Under HR TEM, whose electron beam is more collimated than that of HC TEM, we observed the formation of nanodroplets at an electron dose rate of 180 e^−^/(Å^2^ s) at an electron acceleration voltage of 200 kV. Nanodroplets formed from the thin liquid film fluctuations upon Rayleigh–Taylor instability, as we (71) and others (17) observed previously. The liquid film became thin (176 s), subsequently dewetted (297 s), and finally beaded up on both sides of the inner wall (447 s) (*SI Appendix*, Figs. S8 and S25 and Movie S8).

**Fig. 2.**
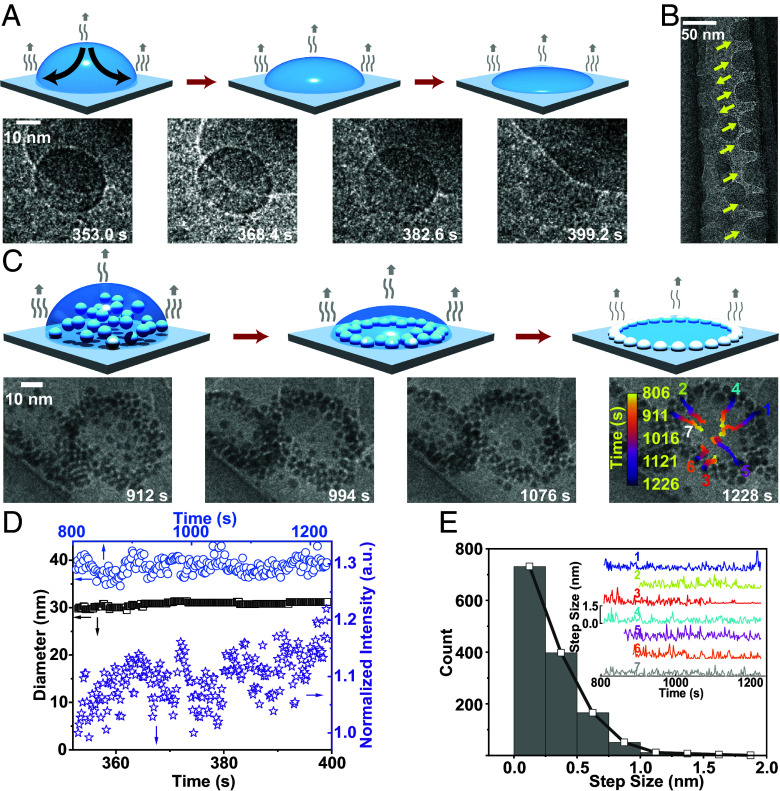
The formation of a nano coffee ring in a pinned nanodroplet (Movie S10). (*A–C*) Diagram (*Top* row) and time-lapse electron micrographs (*Bottom* row) of evaporating droplets: an evaporating plain droplet in *A* and its side view (highlighted by yellow arrows) in *B*; and a nanoparticle-containing droplet (*C*). Time zero denotes when the electron beam was on. The scale bar is 10 nm—details in *SI Appendix*, *Text*. In (*C*), the center-of-mass position of nanoparticles was tracked and superpositioned on the image of 1,228 s, and the color code indicates the time. (*D*) Quantifying changes in droplet diameter and mean intensity for *A* (squares and stars) and *C* (circles). Arrows point to the corresponding axis. (*E*) The step size distribution of nanoparticles of *C*. *Insets*: traces for individual nanoparticles, matching 1 to 7 in *B*. The solid black line is the guide to the eyes.

A pinned droplet is a prerequisite for forming a nano coffee ring, consistent with the microscale scenario (1). We observed evaporation of plain droplets (no nanoparticles) [150 e^−^/(Å^2^ s)] (Fig. 2*A* and Movies S9 and S10a) and droplets containing nanoparticles [900 e^−^/(Å^2^ s)] (Fig. 2*C* and Movies S10b and S11). The circular shape of the nanodroplet was retained as the evaporation proceeded (Fig. 2 *A*–*C*). The average pixel intensity of the droplet increased, capturing the loss of liquid (Fig. 2*D*). Benefiting from the nanopipette’s circular geometry, we captured both the top (Fig. 2*A*) and side views (Fig. 2*B*) of the nanodroplets. The contact angle was larger than 90°, confirming that higher evaporation should occur at the three-phase contact line; therefore, the liquid should flow from the center to the edge to compensate for the liquid loss. Consequently, nanoparticles changed from a scattered distribution (912 s) to a ring (1,228 s). Individual nanoparticles traveled up to 25 nm, while the diameter of the nanodroplet was retained at 40 nm. The radial outward flow was robust with the presence of surface interactions: the motion of nanoparticles was arrested (with step size less than 0.25 nm/s for ~80% of the time) with leaps (larger than 0.3 nm/s) occurring intermittently, which resembled drunken man’s trajectory (Fig. 2*C*, *Inset* of Fig. 2*E*). The step size distribution exhibited a strong non-Gaussian tail (Fig. 2*E*). Strikingly, although the center-of-mass of the droplet had changed ~10 nm, as the consequence of common surface effects observed under both optical and electron microscopes (*SI Appendix*, Fig. S26) (16, 70, 72, 73), the pinned shape and the outward transport of nanoparticles were unperturbed (*SI Appendix*, Fig. S27).

### The Reverse of a Nano Coffee Ring.

**Fig. 3.**
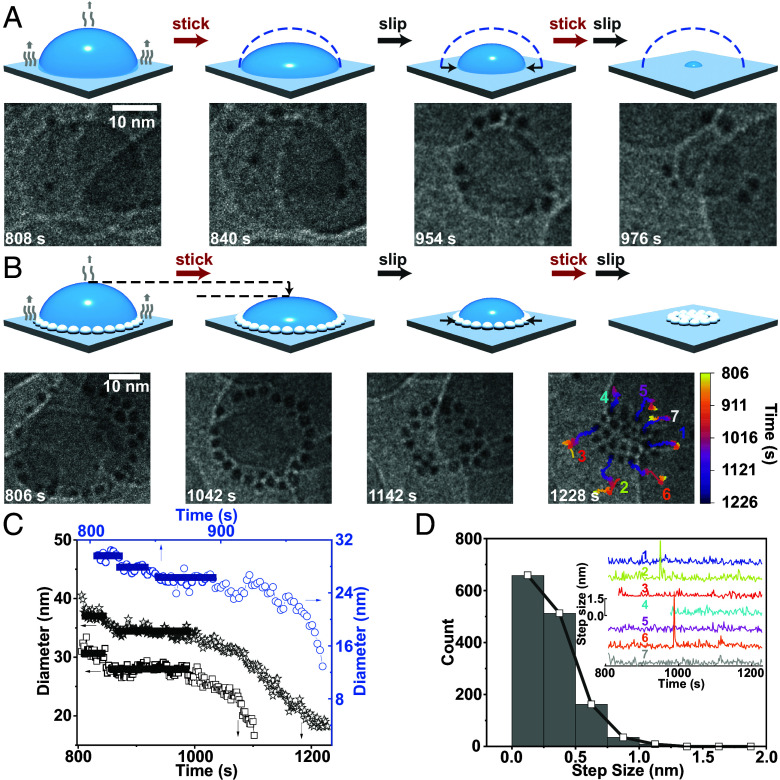
The reverse of a nano coffee ring in a “stick–slip” nanodroplet (Movie S12). (*A* and *B*) The schematic diagram (*Top*) of a droplet undergoes repetitive “stick–slip” transitions and the time-lapse LP-TEM images (*Bottom*) of a plain droplet (*A*) and a droplet containing nanoparticles (*B*). Color coding indicates the time: yellow for the initial and purple for the final. Time zero denotes when the electron beam was on. The scale bar is 10 nm. (*C*) The time-dependent changes of droplet diameters for data in *A* (circles) and *B* (stars: nanoparticles included, squares: nanoparticles excluded) are plotted as a function of time. (*D*) The step size distribution of individual particles in *B*. *Insets*: the traces of individual nanoparticles, matching 1 to 7 in *B*. The solid black line is the guide to the eyes.

Tuning contact line dynamics can effectively reverse nano coffee rings. By raising the dose rate from 150 to 900 e^−^/(Å^2^ s), some droplets appeared to less firmly adhere to the quartz surface while the contact line underwent “stick–slip” transitions (*SI Appendix*, Fig. S26) as it moved inward radially due to evaporation (74). We observed the disruption of the ordered ring pattern (Movies S11 and S12). At the initial stage, droplet height decreased to maintain the pinned periphery to compensate for liquid loss (Fig. 3 *A* and *B* and Movie S12). The contact angle became smaller, and a “slip” occurred when the surface tension surpassed the pinning energy. The nanodroplet then re-established an equilibrium, exhibiting the same contact angle as the initial state. The contact line repeated “stick–slip” process until the droplet fully evaporated, as was the case for microdroplet (75). When the electron beam dose rate was increased from 21 to 450 to 550 e^−^/(Å^2^ s), the chances of observing “stick–slip” also increased (*SI Appendix*, Fig. S28 and *Text* and Movies S13 and S14). The presence of nanoparticles did not change the qualitative pattern (Fig. 3*C*). In both cases, for the similar droplet diameters (35 to 50 nm), we observed 2 to 3 plateaus capturing the “stick” process, each lasting 30 to 100 s and 2 to 3 monotonic decays of 3 to 20 nm capturing the “slip” process, each lasting 20 to 50 s. At the early stage, up to 50 s, the contact line facilitated the nanoparticle to assemble into an ordered ring, in which some neighbor nanoparticles coalesced in less than a second (*SI Appendix*, Fig. S29 and Movies S5a and S15). The contact line did not function to energize the particles, as the coalescence rate was similar to those seen outside of the droplet (*SI Appendix*, Fig. S30). When the droplet diameter decreased to 20 nm (1,142 s), half of its original diameter, the ring (1,042 s) started to disassemble and broke down completely as the droplet dried out (1,228 s), leaving behind a pancake. Overall, the particles moved inward radially, opposite to the direction of the formation process, which exhibited similar trajectories but with fewer frequencies of large step sizes (Fig. 2*E*, *Inset* of Fig. 3*D*). Therefore, the step size distribution of the reverse process was more like the Gaussian distribution (Fig. 3*D*) than the ring forming process (Fig. 2*E*). The attenuated non-Gaussian feature indicated the weakened directionality of forces at play.

### Comparisons between Nano Coffee Ring and Micro Coffee Ring.

Our observations implied that the forces at play for forming a nano coffee ring are different from the microscale. Individual particles’ mean squared displacement (MSD) in four scenarios were compared: (i) the formation of a micro coffee ring (*SI Appendix*, Fig. S31 and Movie S16), (ii) the formation, (iii) the reverse of a nano coffee ring, and (iv) simulated Brownian particles (*SI Appendix*, Figs. S32–S34). In contrast to (iv), in which slopes from the MSD plots exhibited a narrow Gaussian distribution around the peak center at one, particles in (i) always display slopes larger than one due to the dominating effect of the convective flows that drive the directed motion of particles. For (ii), the slopes fluctuate around one with a broad distribution, indicating an attenuated directionality due to the tight competition between the thermal fluctuations, surface forces, and flows. Interestingly, slopes for (iii) were consistently below one, suggesting a dominating role of surface adsorption that hinders diffusion.

**Fig. 4.**
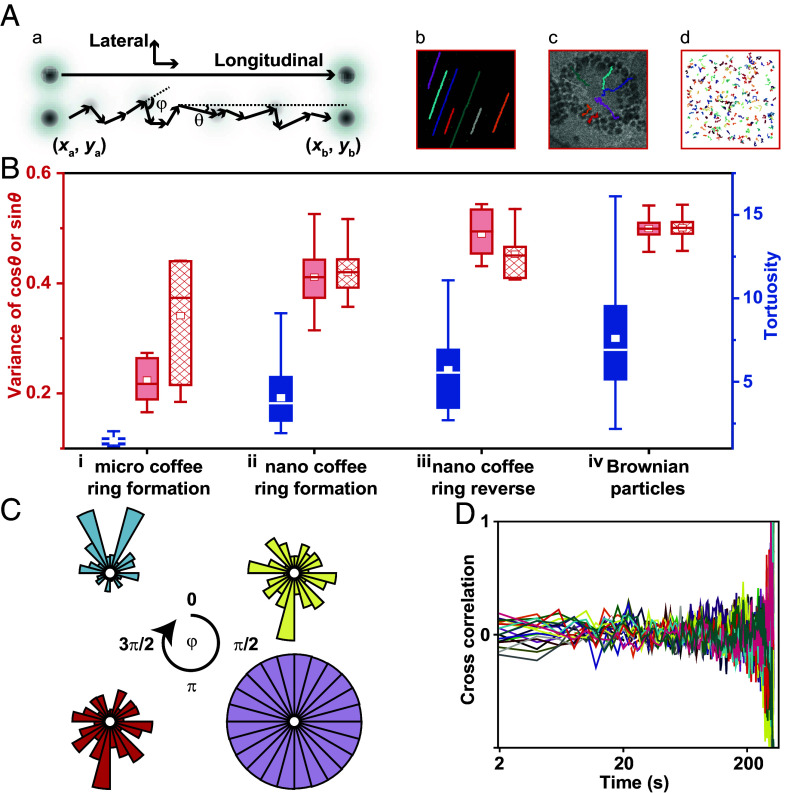
Comparisons of nano coffee ring to micro coffee ring. (*A*) (*a*) Schematic depiction of a particle motion without or with thermal fluctuation and definition of longitudinal and lateral direction. (*b*–*d*) Particle trajectories for the cases (*i*), (*ii*), and (*iv*) as described in the text. (*B*) Comparisons of tortuosity (blue symbols), the variance of cos*θ* (red filled symbols), and the variance of sin*θ* (red grid symbols) of the particles for the four cases (*i*–*iv*). (*C*) Distributions for the four cases (*i*–*iv*) in blue, yellow, red, and purple. (*D*) Cross-correlation of speed for every two particles in (*ii*) the nano coffee ring formation, *n* = 7.

We define *θ*, *φ*, and tortuosity to describe the trajectory and compare the effectiveness in executing the directed motion (*SI Appendix*, *Text* and Fig. 4 *A* and *B*). The variance of cos*θ* and sin*θ* described the fluctuations in the direction of outward flow and its tangential direction, respectively. The tortuosity represents the trajectory deviation from the shortest distance: the smaller values indicate more effective directed motion. Ranking from low to high are (i), (ii), (iii), and (iv) (Fig. 4*B* and *SI Appendix*, Fig. S35). *φ* is defined as the angle between two adjacent steps. It is a circle for Brownian particles. Abundance in 0 or 2π indicates a higher probability of executing directed motion, as shown for (i). In contrast, abundance in π shows constant flips in moving directions due to thermal fluctuations, as shown for (ii) and (iii) (Fig. 4*C*). Chances of having forward steps were higher in (i) but smaller in (ii) than in (iii), which appeared as backward motion.

We found no cross-correlation between the individual two particles in their instantaneous speed for all four scenarios (Fig. 4*D*), in cos*θ*, sin*θ*, longitudinal, and lateral velocities (*SI Appendix*, Fig. S36 and *Text*). This finding suggests the length scale of coupling is smaller than the neighboring distances of the selected particles, 5 to 10 nm. Yet, the interactions could shape nanoparticles into an ordered pattern with five times larger size, 40 to 50 nm.

## Discussion

In contrast to microdroplets, the contact line dynamics of nanodroplets are more susceptible to changes. Interactions between liquid-quartz determine a droplet’s pinning energy at the microscale, while interactions of liquid-nanoparticle and nanoparticle–nanoparticle become critical for nanodroplets (9, 10). The presence of nanoparticles can increase the lifetime of nanodroplets by a factor of about five, regardless of whether the three-phase contact line was pinned firmly (Fig. 2 *A* and *C*, 70 s versus 300 s) or loosely (Fig. 3 *A* and *B*, 90 s versus 400 s), which usually makes no difference for microdroplets. When the particle concentration is too high, we failed to observe the nano coffee ring phenomenon (*SI Appendix*, Fig. S37 and *Text*). The “stick–slip” transitions usually create concentric ring patterns for the microdroplet (75) but can reverse the nano coffee ring patterns.

In the microdroplets, a moving contact line (76, 77) or a faster evaporation rate (11, 77, 78) could suppress the coffee ring effect before its formation and create uniform patterns; the consequence is similar to the particle shape effect (2) that changes the particle alignment and adsorption at the air–liquid interface (2, 78). For nanodroplets, at the moving contact line, nanoflows and the hydrodynamic interactions that align nanoparticles were surpassed by the surface interactions and particle–particle repulsions that randomize the nanoparticles, which suggest a different underlying mechanism.

The capillary flow carries spherical particles to the contact line in pinned droplets (1). We measured its magnitude at the nanoscale. Based on the typical droplet volume (50 nm in diameter) and evaporation time (200 s), we estimated a capillary flow velocity of 0.11 nm/s (*SI Appendix*, *Text*). The number is consistent with the results obtained from single particle tracking, i.e., it took 200 s for a nanoparticle to travel from the center to a droplet’s rim. It is true that our inner wall surface is slightly curved since the droplet projected an angle of ~10°; our estimation is only slightly on the lower bound (*SI Appendix*, Fig. S38 and *Text*). Marangoni flow induced by temperature or surface tension gradients is important for microdroplets (3). However, the thermal Marangoni flow from the electron beam-induced heating effect is negligible (9, 10) as the heat dissipation is instantaneous, 10 ps (*SI Appendix*, *Text*). The solute Marangoni effect is also insignificant due to the absence of surfactants. Electroosmotic flow may be induced by a local electrical field from heterogeneous surface charging effects; however, such flow is usually unidirectional rather than radial.

The capillary flow is coupled with the surface effect. Occasionally, particles showed a larger velocity, up to 1.2 nm/s, than the force provided by capillary flow, estimated to be 10^−3^ pN based on Stokes law. The work to move away a nanoparticle 25 nm is approximately 10^−3^ *k*_B_*T* (*SI Appendix*, *Text*). Curiously, particles executed directional motions despite such a small number relative to thermal energy, *k*_B_*T*. This discrepancy confirms that diffusion in liquid cells is much smaller than in bulk solution (69). Early theories predicted that electrostatics and surface fluctuations could couple at tens of nanometers (18), potentially leading to anomalous fluid transport. Our observations suggested that surface-coupled capillary flow leads to collective outcomes at the corresponding scales, with a characteristic coupling distance that is five times smaller.

Electron beams can facilitate liquid evaporation and may trigger the “stick–slip” motion of the contact line from pinning, suggesting the modulation of the liquid–surface sticking energy. However, unlike the aqueous system, where the electron beam-induced charging effect is significant, the observed coffee-ring phenomenon in the organic solution is not largely affected (*SI Appendix*, *Text*). The charging effect is unable to fully explain the phenomena we observed.

We introduce nanopipettes as a versatile, easily accessible liquid cells for ordinary users to conduct LP-EM experiments. Nanopipette is more cost-effective than SiN and more reproducible than GLC. SNR in nanopipette is lower than that of GLC under identical conditions but similar to SiN; nanopipette is more confined than SiN but similar to GLC. The trade-off between confinement and contrast allows us to do HR imaging at the tip and study assembly problems at the larger diameter. When the tip is open, with an appropriate TEM and enough electron beam dose rate, it enables the study of nanoflows, evaporation-induced dynamics, i.e., solution-based process relevant to device fabrication, and chemical reactions with the dual channel geometry (*SI Appendix*, Fig. S39). When the tip is small or closed, it functions as a conventional closed-geometry liquid cell and can be routinely used for material research, including nanoparticle nucleation, growth, and interactions. The tunable properties of nanopipettes (tip diameter and angle) and the sophisticated surface modification methods on silica with silane chemistry (charge or alkyl modification) create a platform for studying the underpinning parameters one by one in a controllable manner. Meanwhile, the method allows the production of different geometries of nanopipettes; the cross-section can be circular or square (*SI Appendix*, Fig. S40 and *Text*), allowing one to study the shape effects when systems are under confinement. The nanopipettes also provide an opportunity to combine with light, heat, and electrochemistry as SiN liquid cells already achieved, potentially benefiting the development of multimodality imaging based on electron microscopes.

## Materials and Methods

We made nanopipette liquid cells by pulling quartz capillaries (10 cm long, i.d. 0.7 mm, o.d. 1.0 mm, product number: QF100-70-10, Sutter Instrument Co.) using a CO_2_-laser-assisted puller instrument (P-2000, Sutter Instrument Co.) as described previously (37). We used copper TEM grids with a mesh size of 100 (Beijing Zhongjingkeyi Technology Co., Ltd.). We used common electron microscopies: JEM-2100 Plus HC equipped with a Gatan One View IS camera of our own Lab; FEI Tecnai F20 (with an X-Max^N^TSR detector for EDXS), T20, and JEM-F200 (for HR image) at Electron Microscopy Laboratory of Peking University. Chemicals include vacuum grease (Shanghai Hushi Laboratorial Equipment Co., Ltd.), Pt(acetylacetonate)_2_ (purity 98%, Shanghai Eybridge Chemical Technology Co., Ltd), oleylamine (C_18_: 80 to 90%, Energy Chemical Co., Ltd, Shanghai), *o*DCB (purity 99%, Energy Chemical Co., Ltd, Shanghai), and carboxylate-modified polystyrene latex beads (Sigma) were used as received. GNRs, gold nanoparticles, Fe_3_O_4_ nanocubes, and LPA-OEG_7_ were synthesized using standard or customized protocols, detailed procedures are explained in detail in *SI Appendix*.

## Supplementary Material

Appendix 01 (PDF)

Movie S1. This movie shows GNRs experiencing diffusion at a wider part of the nanopipette. Each frame is 0.1594 s, played at 60 frames/s.

Movie S2. This movie shows GNRs interacted to form tip-to-tip assembly in the aqueous solution with trajectories of individual particles. Each frame is 0.1594 s, played at 7 frames/s.

Movie S3. This movie shows the electron-induced etching of Au nanoparticles. Each frame is 0.2 s, played at 5 frames/s.

Movie S4. This movie shows the monomer attachment pathway of Pt nanoparticles' nucleation and growth process. Solution: *o*DCB/oleyamine platinum precursor solution. Each frame is 0.2 s, played at 30 frames/s.

Movie S5. This movie shows the nanoparticle coalescence process occurring at the contact line (a) with that in evaporating solvent (b), with trajectories of individual particles tracked for comparisons. Solution: *o*DCB/oleyamine platinum precursor solution. Each frame is 0.2 s, played at 10 frames/s.

Movie S6. This movie shows a GNR grew up to a GNA in the presence of CTAB. Each frame is 0.1594 s, played at 60 frames/s.

Movie S7. This movie shows the dynamics and degradation of ssDNA in D_2_O with contours of ssDNA. Solution: 5 µM ssDNA in D_2_O. Each frame is 0.1594 s, played at 30 frames/s.

Movie S8. This movie shows the liquid film becoming thin, dewetted and finally beaded on both sides of the inner wall. Solution: *o*DCB/oleyamine platinum precursor solution. Each frame is 1 s, played at 10 frames/s.

Movie S9. This movie shows nanodroplets formed and the evaporation of a plain pinned droplet at 150 e/(Å2·s). The solution is an *o*DCB/oleyamine platinum precursor solution. Each frame is 0.2 s, played at 30 frames/s.

Movie S10. This movie shows that the coffee-ring effect occurs for firmly pinned nanodroplets, plain (a) or containing nanoparticle tracers (b), in an *o*DCB/oleyamine platinum precursor solution. Droplet diameters were plotted in real time for comparisons. Each frame is 0.2 s, played at 10 frames/s in (a) and 2 s, 10 frames/s in (b).

Movie S11. This movie shows pinned droplet evaporation with nanoparticles and “stick-slip” droplets with and without particles at 900 e^−^/(Å^2^·s). Solution: *o*DCB/oleyamine platinum precursor solution. Each frame is 0.2 s, played at 30 frames/s.

Movie S12. This movie shows stick-slip dynamics occur for loosely pinned nanodroplets that reverse the nano coffee ring, plain (a) or containing nanoparticle tracers (b), in *o*DCB/oleyamine platinum precursor solution. Droplet diameters were plotted in real time for comparisons. Each frame is 2 s, played at 10 frames/s. Plateaus indicate that the sticking stage occurs at 808–824 s, 806–846 s, and 850–894 s, three times for the plain nanodroplet; 812–840 s, and 860–990 s, twice for the nanodroplet containing nanoparticles.

Movie S13. This movie shows the high ratio of stick-slip to pinning at 450–550 e^−^/(Å2·s) with contours of nanodroplets. Solution: *o*DCB/oleyamine platinum precursor solution. Each frame is 0.797 s, played at 15 frames/s.

Movie S14. This movie shows no evaporation at 21 e^−^/(Å^2^·s). Solution: *o*DCB/oleyamine platinum precursor solution. Each frame is 0.1594 s, played at 60 frames/s.

Movie S15. This movie shows a full view of nanoparticle coalescence at the contact line. Solution: *o*DCB/oleyamine platinum precursor solution. Each frame is 0.2 s, played at 30 frames/s.

Movie S16. This movie shows particles' directed motion by capillary flow with micro coffee ring forming. Solution: 0.65 µL 1.25% carboxylate-modified polystyrene latex beads were dissolved in 400 µL glycerol and 400 µL deionized water. Each frame is 0.02 s, played at 50 frames/s.

## Data Availability

All study data are included in the article and/or supporting information.
