# Supplementary material for: Nanopipette dynamic microscopy unveils nano coffee ring
Source: Proc Natl Acad Sci U S A. 2024 Jul 2;121(28):e2314320121. doi: 10.1073/pnas.2314320121 (PMC11252805; doi:10.1073/pnas.2314320121)
Supplement: Supplementary file 1 — Appendix 01 (PDF) [file pnas.2314320121.sapp.pdf]

Supporting Information for

Nanopipette Dynamic Microscopy Unveils Nano Coffee Ring

Deyi Zhang[1,†], Yi Shao[1,†], Jiayi Zhou[1], Qiangwei Zhan[1], Ziyang Wen[1], Sheng Mao[2], Jingjing Wei[3], Limin Qi[1], Yuanhua Shao[1,*], Huan Wang[1,*]

[1] Beijing National Laboratory for Molecular Sciences, College of Chemistry and Molecular Engineering, Key Laboratory of Polymer Chemistry & Physics, National Biomedical Imaging Center, Peking University; Beijing, 100871, P.R. China.

[2] College of Engineering, Peking University; Beijing, 100871, P.R. China.

[3] School of Chemistry and Chemical Engineering, Shandong University; Jinan, 250100, P.R. China.

*Corresponding author.

Email: wanghuan_ccme@pku.edu.cn or yhshao@pku.edu.cn

Author Contributions: These authors contributed equally to this work: Deyi Zhang, Yi Shao

This PDF file includes:

Materials and Methods

Supporting Information Text

Figures S1 to S40

Tables S1 to S5

Legends for Movies S1 to S16

SI References

Other supporting materials for this manuscript include the following:

Movies S1 to S16

Materials and Methods

Materials. Ordinary quartz circular capillaries (10 cm long, i.d. 0.7 mm, o.d. 1.0 mm, product number: QF100-70-10) were bought from Sutter Instrument Co., Novato, CA. Custom-made quartz square capillaries (10 cm in length, i.d. 0.73 mm, o.d. 0.93 mm) were bought from Beijing Zhong Cheng Quartz Glass Co., Ltd. Copper grids (mesh size 100) were made by Beijing Zhongjingkeyi Technology Co., Ltd. Vacuum grease was purchased from Shanghai Hushi Laboratorial Equipment Co., Ltd. Pt(acetylacetonate)$_2$ (purity 98%) was purchased from Shanghai Eybridge Chemical Technology Co., Ltd. Oleylamine (C_{18}: 80%–90%) and *o*-dichlorobenzene (*o*DCB) (purity 99%) were purchased from Energy Chemical Co., Ltd (Shanghai, China). 3-Aminopropyldimethylethoxysilane (APTES) (purity ≥ 98%) and Trimethoxy(octadecyl)silane (purity ≥ 95%) were produced by Shanghai Macklin Biochemical Technology Co., Ltd. Latex beads (2.5%, carboxylate-modified polystyrene, fluorescent red) were purchased from Sigma (Shanghai, China). Deionized water (DI water) (18.2 MΩ·cm) was generated by the Milli-Q Academic A10 System (Millipore). Single-stranded DNA (ssDNA) (AGA CCG TAA TTG CCT CTG TTC CCA ATC ATC GTT GAT GTC GCT CAC CAG AAT CGC CCA GCA GTT AGG CCG TAT GAT ATG TAA GGT ATA ACG) was purchased from RuiBiotech (Beijing). Methoxy oligo ethylene glycol modified lipoate (LPA-OEG$_7$) was synthesized and supplied by the Lu group(1).

Sample preparation.

Gold nanorods. The gold nanorods (GNRs) were synthesized as previously reported(2). The precursor solution was prepared by adding 0.25 mL of 10 mM $HAuCl_4$ solution to 10 mL of 0.1 M cetyltrimethylammonium bromide (CTAB) solution in a glass vessel. A freshly prepared ice-cold 0.6 mL of 10 mM $NaBH_4$ was then injected into the mixture under vigorous stirring for 10 s. The resulting seed solution was aged for 30 min at 27°C before use. For the growth solution, 100 mL of 20 mM sodium oleate (NaOL) solution was mixed with 74 mL of 0.1 M CTAB solution and 26 mL of water in a beaker flask under mild stirring and thermostated at 30°C in a water bath, followed by injection of 3.84 mL of 10 mM $AgNO_3$, which was then left undisturbed for 15 min at 30°C. 10 mL of 10 mM $HAuCl_4$ solution was added. After 90 min of undisturbed standing, the solution became colorless. 1.2 mL of 37% HCl was introduced, followed by another 15 min of undisturbed stewing. 1.2 mL of 20 mM ascorbic acid (AA) solution was added, and the mixture was vigorously stirred for 30 s. Finally, 0.08 mL of the seed solution was added to the growth solution, and the reaction mixture was left undisturbed overnight at 27°C. This resulted in the formation of a GNR dispersion with a GNR particle concentration of approximately 0.6 nM, estimated using the Lambert-Beer law with a molar extinction coefficient of 5.5×10^9 M^{-1} cm^{-1} for GNRs. The GNR dispersion was centrifuged, and the GNRs were redispersed in 1 mM CTAB solution for further use.

Preparation of Fe_3O_4 nanocubes. 261 mg of iron oxide red powder, 4.1 mL oleic acid (OA), and 20 mL 1-octadene (ODE) were mixed in a 250 mL three-neck flask and then heated to 110°C in a vacuum for 30 min to remove moisture and oxygen. Then, the vacuum pump was shut down, and the mixture was heated to 320°C under N_2 protection. After 80 min, black precipitates were formed, indicating the formation of Fe_3O_4 nanocubes. The synthesized ferric oxide nanocube was washed and dispersed in chloroform with 10 mg mL^{-1}.

Pt precursor solution. We chose precursor solutions commonly used for in-situ transmission electron microscopy (TEM) imaging of Pt nanoparticles, in which the nucleation dynamics have been observed through SiN cells(3) and graphene liquid cells (GLCs) (4). Pt(acetylacetonate)$_2$ was dissolved in a mixture of oleylamine/*o*DCB (1:9 v/v) at 3.33 mg/mL.

Gold nanoparticle solution. Negatively charged Au nanoparticles (10 nm in diameter) modified with citrate ligand were diluted ten times with DI water and used without further preparation.

Preparation of micelles. LPA-OEG$_7$ was dissolved in DI water to get a concentration of 100 mM. The solution is then vortexed for 30 s.

Preparation of carboxylate-modified polystyrene latex beads solution. 0.65 μL 1.25% Latex beads solution was dissolved in 400 μL glycerol and 400 μL deionized water. The solution is then vortexed for 10 s.

Fabrication of circular quartz nanopipette liquid cells. Quartz nanopipettes were fabricated as previously reported, with slight modifications to the standard protocol(5). Specifically, quartz capillaries were loaded on a CO_2-laser-assisted puller instrument (P-2000, Sutter Instrument Co.). The pulling parameters used are listed in table S1. We used a syringe to inject an appropriate amount of sample solution from the wide-open end of the pipette to load the sample solution. The solution flew to fill up the nanopipette with the help of filament by capillary action. Let it sit briefly and pop any air bubbles in the tube with fingers. The nanopipette was cut by micro scissors from the original capillary at the tip, typically ~0.5–1 mm, with a tiny amount of vacuum grease so its wide-open end could be sealed and lay flatly on TEM grids for imaging. We used another capillary to facilitate the soft landing of nanopipettes on the TEM grid to avoid hard crashing. A schematic illustration of the loading sample into the quartz nanopipette process is shown in fig. S1.

Fabrication of custom-made quartz square nanopipette liquid cells. Square quartz capillaries whose inner surface is flat were loaded on P-2000 with parameters of the last row of table S1. Other fabrication processes are the same as those of ordinary quartz capillaries. In scanning electron microscopy (SEM) imaging, the shaft of the square nanopipette was facing the camera, showing a near square-shaped tip (fig. S40a). TEM images of the quartz square nanopipette show the positions of the four edges of the square (black lines in the nanopipette) rotate as the sample holder rotates (fig. S40b–c).

Surface modification of quartz nanopipettes.
Alkylation modification. The quartz nanopipettes were first cleaned with acid piranha (concentrated H_2SO_4/30% H_2O_2, v/v, 7/3) and then silanized with 46.5 mM trimethoxy(octadecyl)silane in ethanol for 50 min at room temperature. After silanization, the quartz nanopipettes were rinsed with ethanol to remove unreacted silanes. Finally, they were dried at 80°C for 1 h.
Positive charge modification. The quartz nanopipettes were cleaned with acid piranha (concentrated H_2SO_4/30% H_2O_2, v/v, 7/3) and silanized with 0.2% APTES in ethanol for 0.5–1 h at room temperature. After silanization, the quartz nanopipettes were rinsed with ethanol to remove unreacted silanes. Finally, leave them overnight and dry at 80°C for 1 h.

Fabrication of GLCs. We followed protocols as previously reported(1). About 0.10 μL sample solution was dropped onto a graphene-covered TEM grid. The grid containing the sample solution was flipped to place onto the 3–5 layers of graphene floating on a 0.100 M ammonium persulfate aqueous solution. GLCs form after several minutes and are ready for TEM imaging.

Liquid-phase TEM imaging. The reported TEM images were collected with different TEMs for different purposes. Nano coffee ring experiments in quartz nanopipette liquid cells were imaged with FEI Tecnai F20 (Gatan One View IS camera) and T20 (Orius SC1000A camera) with a standard sample holder operating at an acceleration voltage of 200 kV, located at the Electron Microscopy Laboratory of Peking University. Nanoparticle and soft material dynamics and repeats of nano coffee ring experiments in quartz nanopipette were imaged with JEM-2100 Plus HC of our own Lab (Gatan One View IS camera). High-resolution images were imaged with JEM-F200 from Xiao Xu Zhao's Lab. All nano coffee ring data were collected by F20 unless otherwise stated; a 150 μm condenser aperture size was used (F20, Thermofischer). Energy Dispersive X-ray spectroscopy (EDXS) images were taken on Tecnai F20 with an X-Max^{N}TSR detector. The in-situ data were recorded at 2 k × 2 k size. GLC experiments were conducted with JEM-2100 from Analytical Instrumentation Center of Peking University (Gatan One View IS camera) with a standard single sample holder operating at 80 kV and with T20.

Imaging conditions and statistics.

Fig. 1B. Sample solution: 0.24 nM Au nanorods and 1.0 mM CTAB in aqueous solution. Data were acquired at 200 kV, dose rate 1–10 $e^-/(Å^2·s)$.
Fig. 1C. Data from 272 nanopipettes on 272 EM grids, 185/272 containing liquid; 454 EM grids for GLC experiments, among which 283/454 grids have 909 liquid cells). Imaging conditions: 200 kV, dose rate 0–900 $e^-/(Å^2·s)$ for quartz nanopipettes; 80 kV, dose rate 1–107 $e^-/(Å^2·s)$ for GLC.
Fig. 1D: Sample solution: 1 μM ssDNA (left, bottom panel adapted from ref. (6)), 100 mM LP-OEG_7 in H_2O (middle, bottom panel adapted from ref. (1)), Fe_3O_4 nanocube in *o*DCB (right). Data were acquired at 200 kV for nanopipettes, 80 kV for GLCs, and a dose rate of 2–53 $e^-/(Å^2·s)$. See fig. S5 for details.
Fig. 2A and 2B: Data were acquired at 200 kV, dose rate 150 $e^-/(Å^2·s)$.
Fig. 2C, 3A, and 3B. Data were acquired at 200 kV, dose rate 900 $e^-/(Å^2·s)$.

Image processing and data analysis. All electron images were processed and analyzed with Image-J and custom-written Python code (6). The wall of nanopipette or anchored particles is selected as the reference to correct the drift of the image by the "template-matching" function of Image-J. Gaussian blur was applied onto images for denoising purposes, typically 0.8–2.0. The center-of-mass changes of sample objects were tracked using the Image-J plugin Manual Tracking or open-source code Trackpy(7). The intensity, area, and Feret's diameter of sample objects were obtained using the Image-J measurement.
The data processing involved in Fig. 4, including calculations of tortuosity, various angle parameters, and simulation of Brownian motion particles, were all done using custom-written code.

Confocal imaging. Confocal images were taken from our laboratory on Leica Stellaris 5 with resonance scanning mode (256 × 256 pixels, exposure time 20 ms).

Calculation of the Signal-to-Noise-Ratio (SNR). We compared the SNR of the same sample in the nanopipette and the GLC at their optimal imaging conditions, allowing us to obtain images of sufficient contrast without damaging the liquid cells or causing excessive radiolysis products. The SNR of the peaks in the radially averaged TEM images of samples (fig. S5–S6) were calculated using equation 1(8):

$$SNR = \frac{|I_{signal} - I_{noise}|}{\sqrt{I_{noise}}} \tag{1}$$

where I_{signal} is the pixel intensity of liposome, and I_{noise} is the pixel-averaged intensity of three randomly selected background areas (liquid areas without samples).

Estimating liquid retention time in the nanopipette by Kelvin Equation and Hertz-Knudsen equation. The lasting time *t* can be calculated by equation 2:

$$t = \frac{\rho V}{u} = \frac{\rho V}{JSM} \tag{2}$$

Where *V* is the total volume of liquid, ρ is the density of the liquid, *u* is the mass evaporating per second, *J* is the flux of the gas molecules, *S* is surface area, and *M* is the molar mass of the liquid molecules. We assume that 300 μm long from the tip of the nanopipette is filled with liquid so that the range of *V* is counted and calculated $(8.67 \pm 3.90) \times 10^{-15}$ m^3 ($n = 11$, ± SD).
To estimate *S*, we utilized the formula for the surface area of a spherical cap by equation 3:

$$S = 2\pi r^2[1 - \sin(\alpha - \beta)] \tag{3}$$

Where *r* is the radius of curvature along the curved interface, α is the angle between the tangent of the curved liquid surface and the inner wall of the tip, and 2β is the angle between the two inner walls of the tip, as shown in Fig. 1E. The range of 2β is counted and calculated $(7.29 \pm 1.68)°$ ($n = 32$, ± SD).
A simple geometric derivation yields *r* by equation 4:

$$r = \frac{d}{2\cos(\alpha - \beta)} \quad (4)$$

where d is the tip diameter of the nanopipette, which can be determined from EM images.
To estimate J, the Hertz-Knudsen equation can be given as equation 5:

$$J = \frac{p}{\sqrt{2\pi MRT}} \quad (5)$$

where p is saturated vapor pressure at a curved interface, R is the universal gas constant, and T is temperature.
Kelvin equation (equation 6) describes the change in vapor pressure due to a curved liquid-vapor interface, as shown in Fig. 1E:

$$\ln\frac{p}{p_0} = -\frac{2\gamma M}{r\rho RT} \quad (6)$$

Where p_0 is saturated vapor pressure at the flat interface is the surface tension of the liquid.
According to equations 2 to 6, the lasting time t can be estimated by equation 7:

$$t = \frac{2\rho V\sqrt{2\pi MRT}\cos^2(\alpha - \beta)}{\pi d^2 M p_0[1 - \sin(\alpha - \beta)]e^{\frac{-4\gamma M\cos(\alpha-\beta)}{d\rho RT}}} \quad (7)$$

Calculation of tortuosity. Imagine a particle moving from point A (x_a, y_a) to point B (x_b, y_b) along a specific path on a two-dimensional plane. According to the particle's position coordinates at a particular moment, and the previous moment ((x_t, y_t) and ($x_{t-\Delta t}$, $y_{t-\Delta t}$), respectively), we calculate the step size Δs at this moment by equation 8:

$$\Delta s = \sqrt{(x_t - x_{t-\Delta t})^2 + (y_t - y_{t-\Delta t})^2} \quad (8)$$

Then we calculate the tortuosity of the particle trajectory by equation 9:

$$Tortuosity = \frac{\sum \Delta s}{\sqrt{(x_b - x_a)^2 + (y_b - y_a)^2}} \quad (9)$$

For several particles in the same view, we quantify the distributions of tortuosity of Fig. 4 to show the difference between micro, nano, and simulated Brownian motion. Note that the motion of all particles should be divided into segments of an equal number of steps to ensure the comparability of different trajectories.

Definition of θ and φ. Imagine a particle moving from point A to point B along a specific path on a two-dimensional plane. Taking vector **AB** as the reference vector ($x_b - x_a$, $y_b - y_a$), subtract the particle's position coordinates at a particular moment from those at the previous moment to obtain the instantaneous directional vector ($x_t - x_{t-\Delta t}$, $y_t - y_{t-\Delta t}$) of the particle at that moment. The angle between this instantaneous directional vector and vector **AB** is defined as θ_t. We calculate cosθ_t and sinθ_t by equations 10 and 11:

$$\cos\theta_t = \frac{(x_t - x_{t-\Delta t})(x_b - x_a) + (y_t - y_{t-\Delta t})(y_b - y_a)}{\sqrt{(x_t - x_{t-\Delta t})^2 + (y_t - y_{t-\Delta t})^2} \times \sqrt{(x_b - x_a)^2 + (y_b - y_a)^2}} \quad (10)$$

$$\sin\theta_t = \frac{(x_t - x_{t-\Delta t})(y_b - y_a) - (x_b - x_a)(y_t - y_{t-\Delta t})}{\sqrt{(x_t - x_{t-\Delta t})^2 + (y_t - y_{t-\Delta t})^2} \times \sqrt{(x_b - x_a)^2 + (y_b - y_a)^2}} \quad (11)$$

According to cosθ_t and sinθ_t, we can get θ_t in the range of [0, 2π). Cosθ_t can represent the particle's longitudinal motion, while sinθ_t can represent the particle's lateral motion. In the case of $x_t = x_{t-\Delta t}$ and $y_t = y_{t-\Delta t}$, we specially set cosθ_t = 0 and sinθ_t = 0, representing that the particle has no longitudinal or lateral motion. To define the set of all θ_t, we calculate the variance of one particle's cosθ and sinθ to reflect fluctuation in longitudinal and lateral directions, respectively. For several particles in the same view, we quantify the distributions of variance of cosθ or sinθ as Fig. 4 to show the difference between micro, nano, and simulated Brownian motion of fluctuation in longitudinal or lateral directions.
The angle between vector ($x_t - x_{t-\Delta t}$, $y_t - y_{t-\Delta t}$) and vector ($x_{t-\Delta t} - x_{t-2\Delta t}$, $y_{t-\Delta t} - y_{t-2\Delta t}$) is defined as φ_t. To simplify the calculation, we calculate φ_t by equation 12:

$$\varphi_t = \theta_t - \theta_{t-\Delta t} + 2m\pi, m \in \boldsymbol{Z} \tag{12}$$

We add $2m\pi$ to ensure the t range is [0, 2π). Likewise, to represent the set of all t, we calculate the distribution in Fig. 4 to show the difference between micro, nano, and simulated Brownian motion of their directionality. Note that we ignore all $\varphi_t = 0$ when calculating the distribution of φ, for particles without motion in two adjacent periods can neither be counted as directional nor steering motion, and the probability of a particle being perfectly isotropic in two adjacent periods is extremely low.

Calculation of cross-correlation function. The cross-correlation function of speed-speed, $\cos\theta$-$\cos\theta$, $\sin\theta$-$\sin\theta$, or velocity-velocity (see as two signal sequences $f_1(t)$ and $f_2(t)$) for every two trajectories were calculated by equation 13:

$$C_{f_1,f_2}(\tau) = \frac{\sum_{t=0} f_1(t) f_2(t+\tau)}{\sqrt{\sum_{t=0}(f_1(t) - \overline{f_1(t)})^2 \sum_{t=0}(f_2(t) - \overline{f_2(t)})^2}} \tag{13}$$

The calculating of $C_{f_1,f_2}(\tau)$ was based on trajectories lasting for at least 53 frames.

Supporting Information Text

Sample search time. Specifically, in the GLC experiment, counting from the electron beam, we search liquid cells upon moving the TEM grid position and observing from the phosphor viewing screen; it usually takes about 20 min to find an appropriate liquid pocket (typically a long strip of 100–350 nm width filled with liquid) as we describe in details in our previous paper(9): Although the chances are substantial to find smaller pockets, ~80% counting from 118 pockets from 43 grids, these pockets usually were empty or contained a few nanoobjects that were visually different from sample molecules; high-quality large pockets that contain sample molecules are usually ~20% among all pockets that we find. For the nanopipette experiment, as there is only one liquid cell, we easily locate the position of the nanopipette due to its recognizable conical shape and much larger other part (0.5–1 mm) (fig. S1c), which takes only around 1 min. To summarize, the size and the location of the GLCs are random due to their inherent formation mechanism; therefore, it requires a longer time to find the sample. In contrast, the nanopipette does not have the problem of random sample location; thus, the search time is significantly reduced.

Nanopipette production time. The GLC experiment takes 4–6 hours to obtain free-floating graphene by etching away the copper substrate. It takes about 30 minutes to prepare the graphene liquid pocket before loading it into the electron microscope(9). (The detailed fabrication procedures are as follows: Step 1, a graphene copper foil is cut into several small squares and flatted with clean glass slides, ~2 min. Step 2, an EM grid is loaded onto each piece of copper foil, ~1 min. Step 3, a small droplet of isopropanol is added onto the grid/foil surface and allowed to dry upon natural evaporation for up to 30 min. Step 4, the foil is turned 90° to check whether the grid is firmly attached, then placed into 0.1 M ammonium persulfate solution, allowing at least 4–6 h for copper to be etched away. Step 5, the excess graphene around the grid is removed by a tweezer, and then the grid is gently placed onto clean filter paper with the graphene side facing up for drying, ~5 min. Step 6, ~0.5 μL of the sample solution is added to the center of the graphene side of the grid, ~1 min. Step 7, the grid is placed gently on a pre-etched, freely floating 3–5 layer graphene with the droplet side facing down, ~1 min. Step 8: after ~5 min, remove the grid and rinse it if necessary. The grids are left for 20 min to allow liquid pockets to form.). In the nanopipette experiment, the Laser-Based Micropipette Puller P-2000 is turned on and warmed up for 15 min before pulling (https://www.sutter.com/manuals/P-2000_OpMan.pdf, Page 13), and then 6 quartz capillaries can be stretched and broken into 12 almost identical nanopipettes within 15 min. Each nanopipette takes 5 min to inject the solution, cut off the tip, and transfer it to the grid.

GNR assembly. The motion and assembly of GNRs suspended in CTAB aqueous solution in a nanopipette were imaged (fig. S2, Movie S2). The GNRs moved fast before 7 s, during which NP1 was assembled with NP2 and NP3 tip to tip (NP2 and NP3 were pre-assembled face to face), consistent with previous reports(10). After 7 s, the particles were trapped and stopped moving. By calculating the MSD of particles, we got the diffusion coefficient of 10^4 nm^2/s, which is among the highest attained with LP-EM, only 2 orders, not 6 to 8 orders, of magnitude smaller than that predicted by the Stokes-Einstein relation.

Au Nanoparticle etching. We observed that at the initial stage (0–50 s), Au NP retained their sizes ~10 nm; as the electron irradiated, Au NP started to shrink and decreased to ~5 nm at 64 s (fig. S13). NP3 disappeared at 74 s; NP1 and NP2 took additional few seconds to fully dissolve at 87 s. Despite the etching rate as their time-lapsed contour indicates (fig. S13b), when projected areas of the particles are plotted as a function of imaging time (fig. S13c), a common transition point emerged at 50 s, after which nanoparticle size started to reduce, indicating a threshold of accumulated dose existed for initiating etching process, ~2.20×10^4 $e^-/Å^2$. A general pattern was observed that after etching started, time-dependent etching rates for each particle (fig. S13d) fluctuated around 0.05–0.2 nm/s for 15–20 s, followed by a large leap to 0.6–1.2 nm/s in few seconds, after which particle vanished concurrently—the time the etching rate leaped slightly depending on the original particle size. However, the particle size at this critical point was similar, 4–5 nm in diameter, ~20 nm^2 in projected area.

Pt Nanoparticle growth and coalescence. Nanoparticle nucleation and growth processes have been widely studied in different types of liquid cells. The platinum precursor solution was encapsulated with nanopipette (EDXS in fig. S14) and imaged at a dose rate of 167 $e^-/(Å^2 \cdot s)$, comparable to previous studies(3, 4). The dose rate is critical, as when it is low, oxidative radical species dominate in the solution (11). As expected, we observed two classical pathways of Pt NP growth: monomer attachment and via coalescence, consistent with earlier reports, as representative time-lapsed EM images shown in fig. S15. In fig. S15a, starting as a homogeneous solution at 0 s, a dozen visually discernible NPs appeared at 76 s; they kept increasing from 1 nm to 1–2 nm at 50 s and to 2 nm at 100 s and 2–3 nm at 150 s, via the well-documented monomer attachment process. Cross-section analysis of NP1 in fig. S15b highlighted the time-dependent increase in diameter. In contrast, in fig. S16, for a coalescence process, we observed that two adjacent particles, both ~1 nm, encountered and formed a visible "neck" (414 s) before they fully merged into the circular shape particle of larger size, ~3 nm (429 s). The curved shape readily discriminates the two pathways from plotting particle area changes as a time function. For the monomer attachment pathway (fig. S15), particle area continuously increases, albeit with a small fluctuation, likely due to the motion blurring. In contrast, particle area fluctuates around a constant value for the coalescence pathway, followed by a steep increase within a second, indicating the completion of growth via coalescence (fig. S16). During the growth process, these particles were mobile as they should be, and the time-lapsed center-of-mass positions are shown. Typically, each particle moved ~10 nm during 70–80 s. Because they are mobile, dynamic blurring causes fluctuation in the projected size: smaller particles diffuse faster and execute larger steps, creating a more significant blurring effect that is harder to resolve. Noticing that these movies were taken at the position where the cell width is ~200 nm, and resolution can be better further towards the tip.

GNR growth. The growth of a GNR in the presence of CTAB in a nanopipette was imaged (fig. S17, Movie S6). Under continuous electron irradiation for more than 100 s, GNR grew from rod-like to arrowhead-like gold nano arrow (GRA) due to the presence of CTAB(2, 12, 13). This growth process is consistent with previous reports(14).

ssDNA dynamics. The dynamics of ssDNA were captured in nanopipette at a dose rate of 8.4 $e^-/(Å^2 \cdot s)$ (fig. S18 and Movie S7), lower than 110 $e^-/(Å^2 \cdot s)$, a dose rate which is believed not to affect the ssDNA dynamics (15, 16). The dynamics were captured at a wall thickness of 10 nm,

similar to the reported 10-nm SiN liquid cell(15). We identified the contour of ssDNA in the images, but due to its low SNR, background speckles are interfering with our identification. The ssDNA molecule may be degraded at 57.07 s with an accumulated dose of 477.6 $e^{-}/Å^{2}$ (fig. S18b).

Electron-beam-induced charging effects. The higher electron dose rate did not lead to more pinning, indicating the pinning does not originate from electrostatic attraction: (i) the stick-slip transitions of the contact line were triggered using a higher electron dose rate (Movies S9–S12); (ii) a higher evaporation rate and a greater ratio of stick-slip to pinning were observed at higher electron dose rate (fig. S28, Movies S14, S15); (iii) assuming an electric field presents within the nanopipette due to beam-induced positive charging like SiN liquid cell(17), droplets would have pinned at one side; however, overlapping of two-dimensional projections of pinned droplets indicate a uniform distribution (Movies S9–S12, S15). Pure quartz has much higher conductivity under electron beam irradiation than that under direct current electric field, with no charge accumulated(18). Meanwhile, the electrostatic repulsion is also insufficiently explanatory to the ring pattern, for (i) the velocity of nanoparticles is essentially uniform (Fig. 2E and 3D) (the magnitude of the electrostatic force is inversely proportional to the square of the distance between the charges), (ii) the droplets did not deform like the previous report(19), (iii) particle motion is directed outward or inward (Fig. 2C and 3B), rather than in a random direction caused by charging(20). It is simulated that increasing the charge of particles can enhance their tendency to form regular packing on the rim. Still, it also increases the distance between neighboring particles, reaching approximately twice the particle diameter(21). In the nano coffee ring in our experiments, the distance between neighboring particles is nearly equal to one particle diameter, similar to the simulation results without charge. To summarize, electron-beam-induced charging has little effect on droplet pinning or nanoparticle concentric ring formation.

Electron-beam-induced heating effects. For the SiN window, it was calculated to be ~4°C, arising at a similar electron dose(22). As the thermal conductivity of quartz (7.7–8.4 W/(m·K)) is 2.5 times smaller than SiN (20 W/(m·K)), there could be a 10°C temperature rise in quartz nanopipette. However, as liquid thermal conductivity is small (0.10–0.20 W/(m·K)) and given our liquid cell has a circular cross-section such that the surface-to-volume ratio is 10^{2} to 10^{3} higher, for example, a typical SiN dimension is 10 μm × 10 μm × 100 nm and a typical dimension for quartz is 1000 nm × 100 nm × 100 nm, the temperature arises in quartz nanopipette can be much smaller than the estimated 10°C (Imaging condition: 200 kV, 150–900 $e^{-}/(Å^{2}·s)$).

Conditions for successful oleylamine droplet evaporation and nano coffee ring experiments. We have conducted a large number of repeated experiments (117 times) (table S5) under different experimental conditions (model numbers of TEM, solvent) to investigate in detail the formation conditions of nanodroplet evaporation, as well as the critical role of electron beam effects in them.

We repeated droplet evaporation experiments on different TEM models, including JEM-2100 Plus HC (86 times), FEI Tecnai T20 (9 times), and Field Emission FEI Tecnai F20 (22 times). Almost all the experiments of droplet evaporation in JEM-2100 Plus HC with Pt precursor solution (66 times) failed: the solutions in these nanopipettes, although with the tips continually open (45 times within 66 times, 45/66, tip diameter = 20–300 nm), whether initially liquid-filled (30/45) or containing bubbles (14/45), remained virtually unchanged under prolonged electron beam irradiation (0–1256 $e^{-}/(Å^{2}·s)$). In the only case close to success (tip diameter = 20 nm) (fig. S20), we continuously imaged the nanopipette at a diameter of 200–420 nm with an electron dose rate of 38–175 $e^{-}/(Å^{2}·s)$, where *o*DCB took 618 s to fade away gradually (fig. S20a–d), leaving the remaining oleylamine film gradually unstable and forming droplets due to Plateau-Rayleigh instability(23, 24); however, after 780 s, the movement of these droplets suddenly weakened (fig. S20e) and changed minimally afterward (780–829 s) (fig. S20e–f). After we finished imaging, we found that the tip had nearly closed (fig. S20g), resulting in an extremely low evaporation rate (fig.

S20h). The phenomenon of the tip closure (small diameter of 20 nm) when imaging at a large diameter (100–450 nm) is common on the JEM-2100 Plus HC (12/86, but only 1/22 for F20). This phenomenon indicates that the irradiation range of JEM-2100 Plus HC on samples is larger than that of high-resolution HR TEM.

JEM-2100 Plus HC has objective lenses with a larger separation between the upper and lower pole pieces, with a distance of up to 10 mm, much larger than the HR TEM, for example, F20 (5.6 mm), leading to a less focused electron beam spot (fig. S19), and thus a larger irradiation area on samples. A larger irradiation area results in lower electron beam energy density. We calculate the energy density (W/m^2) of the electron beam by the following equation:

$$Energy\ density\ = \frac{UN_{\mathrm{e}^-}}{N_{\mathrm{A}}St_{\mathrm{expo}}} \tag{14}$$

where U is acceleration voltage, N_{e^-} is the number of electrons in the irradiation area, which is directly related to the dose rate, N_A is Avogadro's constant, S is the area of the electron beam irradiation region for samples through the pole piece, t_{expo} is the exposure time.

From the equation, it is evident that at the same accelerating voltage and the same electron dose rate, the electron beam in the JEM-2100 Plus HC has a greater irradiation range for the sample, resulting in an energy density that is not as high as that in the F20 or T20. Repeating droplet formation and nano coffee rings in JEM-2100 Plus HC is difficult. This favors imaging of biological samples more sensitive to the electron beam. However, it is detrimental to high-resolution imaging and liquid evaporation, which requires a high electron energy.

In the experiments using T20, we captured images of nanopipette containing oleylamine/*o*DCB, as well as the process of liquid loss (1/3 of total experiments) (fig. S21a–b). Its electron beam is more focused than that of JEM-2100 Plus HC, we can directly irradiated the tip (fig. S21c), to cause it to deform. These results suggest that nanopipette can be used as a standard liquid cell, as well as the liquid cell for studying liquid evaporation process, when we use T20, a standard entry level TEM.

On a high resolution F20, there is a threshold of electron dose rate above which the liquid in the nanopipette can generate enough volume loss to form a film, resulting in liquid droplets due to film instability(23, 24). This threshold is approximately 100–150 $e^-/(Å^2·s)$. At an electron dose rate of 90–100 $e^-/(Å^2·s)$, the liquid loss is very slow (fig. S23), while at a dose rate of 150 $e^-/(Å^2·s)$, the loss of liquid and the generation of droplets were successfully observed (Fig. 2A–B). In addition, a higher electron dose rate is also crucial for the generation of Pt particles, as when it is low, oxidative radical species dominate(11).

Heating contributes little to liquid loss. Our estimation of liquid retention time in nanopipette in a vacuum environment by Equation 7 is the lower limit, for the electron beam effect was not considered, and strong surface interactions between liquid and window material at the nanoscale exist. We have theoretically estimated the electron-beam-induced heating effect: the temperature in quartz nanopipette can be much smaller than 10°C (Imaging condition: 200 kV, 150–900 $e^-/(Å^2·s)$).

We tried to use dichloromethane (DCM) instead of *o*DCB as the main solvent when repeating droplet evaporation using JEM-2100 Plus HC (17 times) (tip diameter = 20–150 nm). DCM has a low boiling point (40°C), so the liquid loss can be easily realized on this HC (high-contrast) TEM. We did observe the loss of liquid and the generation of particles, but what finally formed was not like droplets but a gel-like substance (fig. S24), which may be caused by the oxidation of oleylamine. We also observed that the Pt particles generated in advance disappeared under continuous electron beam irradiation (187 $e^-/(Å^2·s)$), proving the presence of an oxidizing environment in the nanopipette. These observations further indicate that the JEM-2100 Plus HC, due to its lower electron beam energy density, creates an oxidizing environment at low electron dose rates(11), which is not conducive to liquid evaporation experiments that require high electron energy density and the reduction of Pt particles. At least 100–150 $e^-/(Å^2·s)$ on F20 (or any other TEM with focused electron beam spot) is needed to replicate oleylamine droplet evaporation and nano coffee ring. This also suggests that droplet formation cannot be replicated

on the JEM-2100 Plus HC because the oleylamine is oxidized into a gel-like substance, which hinders evaporation.
In summary, suitable experimental conditions are needed to achieve liquid loss and nanodroplet formation: A TEM with high electron beam energy density, a nanopipette with an open tip, and an electron dose rate of ≥ 150 $e^-/(Å^2 \cdot s)$ are favorable for *o*DCB evaporation, Pt particle generation, and fluidity of oleylamine droplets.

Influence of the nanopipette curved surface. The droplets analyzed in the manuscript (Feret's Diameter: 30–50 nm) were positioned at the nanopipette diameter of about 200 nm. Although the contact angle of a droplet on a surface varies with curvature(25), the droplet projects an angle of $\gamma = 9–14°$ to the center of the cross-section of the nanopipette (fig. S38a). With such a small angle, the curvature effect on the contact angle is usually negligible ($\sin\gamma \approx \tan\gamma$). The conical shape leads to an elliptical two-dimensional projection of the droplets (fig. S38b–c). Alternatively, we can observe the contact angle of the droplet in the side view (Fig. 2B); from the droplet's shape, one can estimate the maximum curvature, assuming the elongation in the ellipse droplet (fig. S38b–c) results from distortion to the original circular droplet due to the conical shape of the nanopipette. As the data we captured is a two-dimensional projection image, we underestimated the distance traveled by the particles moving along the short axis and, therefore, slightly underestimated the velocity of the capillary flow experimentally (~10% for particles along the short axis), which has little effect on our conclusions. In fact, due to the surface effects, droplets were found to be inherently distorted from their circular shape at the nanoscale(19); curvature is not the only factor that can result in an ellipsoidal shape; therefore, this estimation of difference defines the upper limit.

Influence of nanoparticle concentration. We simply used Pt nanoparticles as tracers to study the nanoscale coffee ring. When the particle concentration is too low, or there are no particles (Fig. 2A and 3A), capillary flow still dominates in the droplet, which we cannot quantify using nanoparticles. When the particle concentration is too high, repulsive forces between particles may dominate, making it challenging to track capillary flow (fig. S37).

Stability of the nanopipette. Considering mechanical stability, graphene has a Young's modulus as high as 1 TPa(26), and SiN has a Young's modulus higher than 300–400 GPa(27-29). However, GLC is more fragile than SiN because it has only a single atomic layer. The thickness of the quartz nanopipette is comparable to that of the SiN cell, and Young's modulus of the nanopipette is about 70–100 GPa(30-32), which is slightly lower. Considering the stability under electron beam irradiation of the liquid cell, the tip (diameter = 20 nm) was locally irradiated to deform to closed (fig. S11, S20–21). The dose rate was 22 $e^-/(Å^2 \cdot s)$, the accumulated dose in 234 s was 5148 e/Å2, and the nanopipette showed tip closure and deformation. However, the thicker part of the nanopipette is still usable. In Movie S9, we studied droplet evaporation at 150 $e^-/(Å^2 \cdot s)$ at a thick region (diameter = 100–200 nm, wall thickness = 20–50 nm, comparable to SiN cells) for 792 s, resulting in an accumulated dose of 118800 $e^-/Å^2$. Still, the nanopipette was only slightly deformed at a narrow position. For another example, Movie S11 was taken right after Movie S9 in time sequence in the same nanopipette (diameter ≈ 200 nm), with a dose rate of 900 $e^-/(Å^2 \cdot s)$ for 534 s, resulting in an accumulated dose of 480600 $e^-/Å^2$. Still, the nanopipette was only slightly deformed with a decrease in diameter (from 210 nm to 184 nm, only 12%) with such an incredibly high accumulated dose. SiN cell is highly stable; for example, it can tolerate an accumulated dose of over 84200 $e^-/Å^2$(33). However, even SiN liquid cells with good mechanical and chemical stability can experience pressure differences inside and outside the liquid cell due to gas generation, bulging to bulge out, and reduced resolution(34). To solve this problem, increasing the film thickness can decrease resolution while adding micrometer-scale pillars can further increase costs(35). Overall, slight deformation of nanopipettes is foreseeable at such high electron dose rates. The stability of nanopipette under electron beam irradiation is comparable to that of SiN cells. In the presence of an electron beam, SiO_2 can form defects due to electronic

excitation and surface damage due to desorption of its components caused by electron transitions(36-39). Therefore, the tip of the nanopipette, with a thinner wall, is more sensitive to electron beam irradiation than the wider part (fig. S11, S20–21, and Movie S9).

Estimation of flow velocity. The typical evaporation time for a droplet (50 nm in diameter) was 200 s. As the contact angle of oleylamine on quartz is ~140° and it pins, we assume it is a spherical crown to estimate the droplet volume, 4.4×10^4 nm^3, and thus the evaporation flux 2.2×10^2 nm^3/s. As the cross-section is a circle, 2.0×10^3 nm^2, the capillary flow-induced velocity is 0.11 nm/s. This value is within the same order of magnitude and slightly smaller than the measured mean velocity, 0.16 nm/s, as it took 200 s for the nanoparticle to travel from the center to the droplet periphery, 25 nm (Fig. 2C). This slight discrepancy is reasonable, especially considering the presence of surface effect that hindered particle motion. It is thus curious why particles can execute larger velocities up to 1.2 nm/s, exceeding the force provided by capillary flow.

Estimation of forces. We estimated the forces, as viscous force dominates, we use Stoke's law to calculate the forces, $F = 6\pi\mu R v$, where μ is the dynamic viscosity (5.0–20 Pa·s for oleylamine), R is the radius of particle (~2.0 nm), v is the particle velocity that we determined from experiment, ~1.2 nm/s; this estimated the force $F \approx 2.3\text{–}9.0 \times 10^{-4}$ pN; to move 25 nm, then the work is roughly $1.4\text{–}5.5 \times 10^{-3}$ $k_B T$. The number is much smaller than thermal energy, yet we see the directional motion of particles and the absence of jiggling, suggesting that Brownian motion is insignificant. It is then curious to compare the mean velocity of the particle to Brownian diffusion of nanoparticles of this size, ~2 nm, ~$5.5\text{–}22 \times 10^{-15}$ m^2/s according to the Stokes-Einstein equation, the characteristic diffusion time is 0.1 s, indicating diffusion should be must faster than the directional velocity we measured. This inconsistency suggested that diffusion in the liquid cell must be at least four orders of magnitude smaller than the theoretical values. The finding is consistent with the measured nanoparticle diffusion in silicon nitride liquid cell(40), 5–6 orders of magnitude smaller than the bulk. Surface must play a critical role not only for the increased viscosity but also echoes the observed intermittently directional trajectory, unlike at macroscale particle motion driven in flow is continuous.

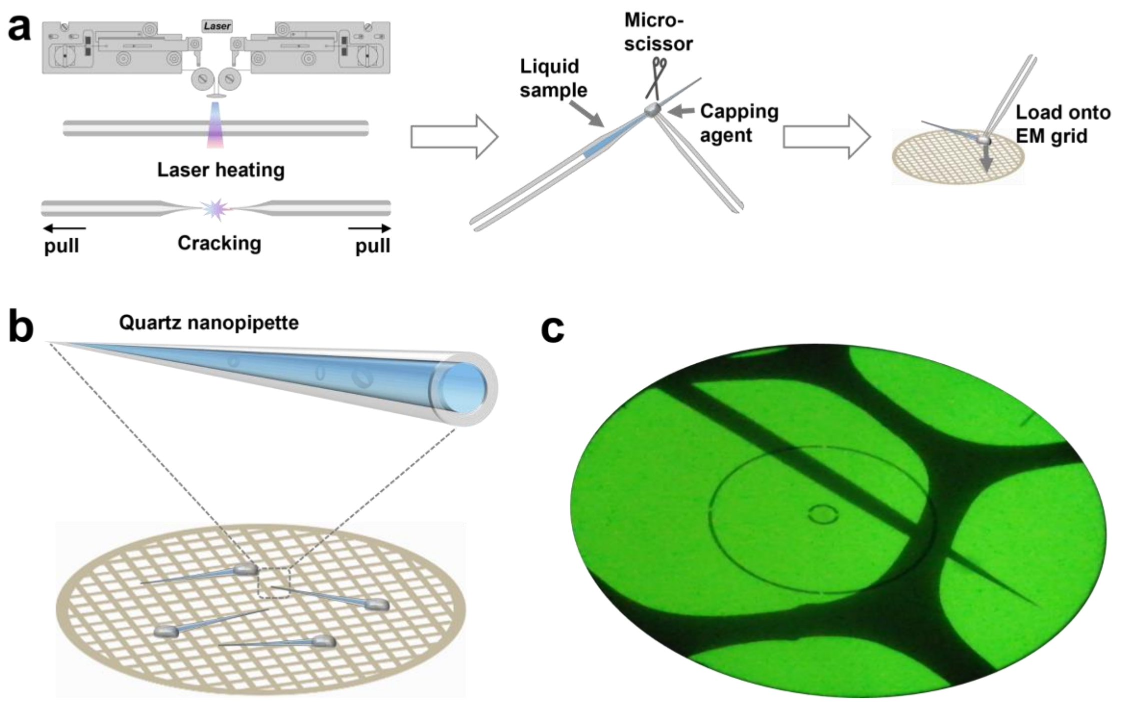

Fig. S1. Fabrication of quartz nanopipettes by laser heating with a puller instrument. (a) Workflow, (b) Schematic depiction of quartz nanopipette on a standard TEM grid, (c) the corresponding zoom-in image on a phosphor screen of EM.

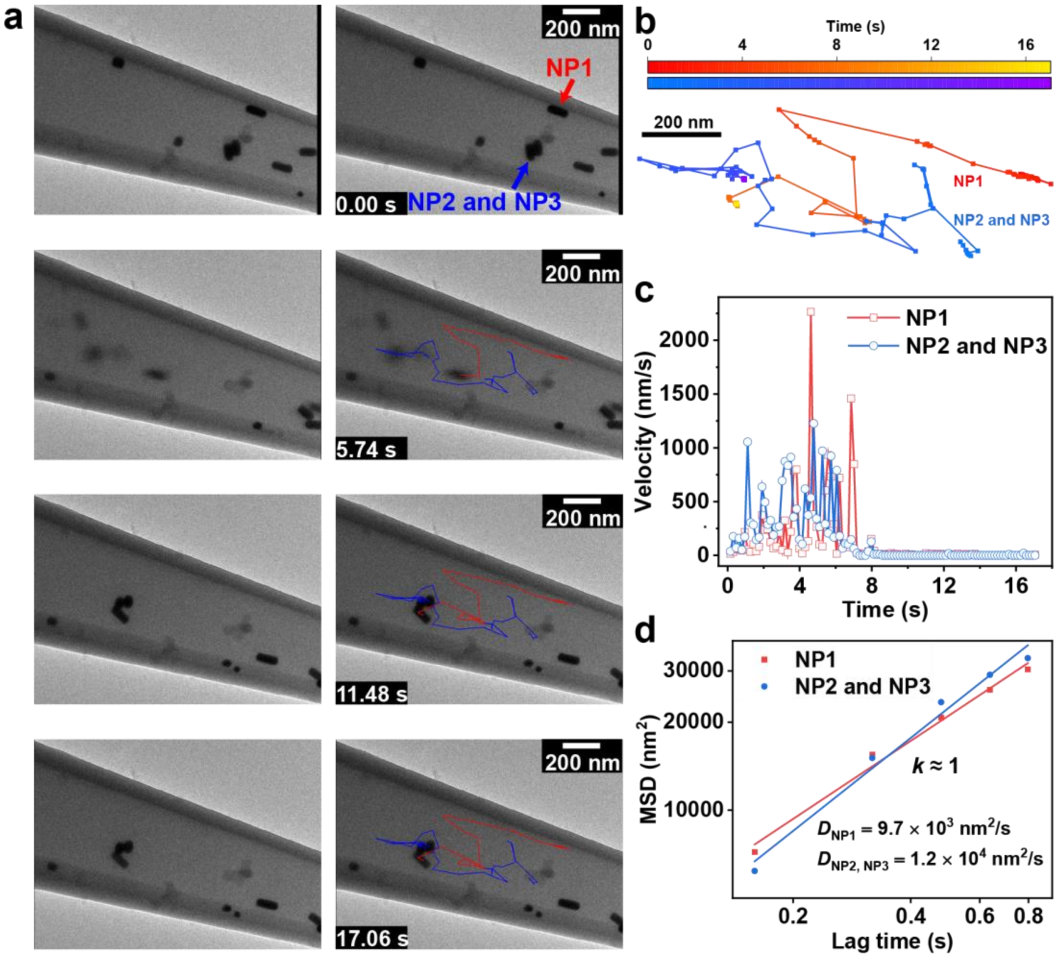

Fig. S2. (a) (Left) LP-EM images show GNRs assemble in nanopipette. (Right) Red line shows NP1 trajectory and blue line shows NP2 and NP3 trajectory. The GNRs diffuse fast before 7 s, during which NP1 assembled with NP2 and NP3 as tip-to-tip interaction (NP2 and NP3 were pre-assembled as face-to-face interaction). After 7 s, the particles were strongly trapped by the surface and appeared frozen in space. Therefore, the four images in the bottom two panels (11.48 and 17.06 s) appeared identical, especially as the images were also applied with Gaussian blur to denoise. But quantitative analysis can reveal difference in their respective positions as shown in S2b–c. See Movie S2 for more information, zoom in of the images and effect of vision persistence help to see the subtle movements. Solution: 0.24 nM GNRs in 1 mM CTAB aqueous solution. Scale bar: 200 nm. Imaging condition: 200 kV, 5.6 $e^{-}/(\text{Å}^2{\cdot}s)$. Images were taken on JEM-2100 Plus HC. (b) Trajectories of NP1 to NP3 quantified. Color bars represent time. The *x* and *y* coordinates of NPs center-of-mass changed rapidly before 7 s, the average change of distance per frame was (55 ± 61) nm, but after 7 s the average change of distance per frame was (1.1 ± 3.0) nm. (c) Dependence of particle velocities on time. The absolute value of velocities of NPs were high before 7 s ((345 ± 383) nm/s) but decreased drastically after 7 s ((6.82 ± 18.9) nm/s). These particles change their direction during movement therefore summating to minimal change of center-of-mass position when comparing different times that several frames apart. consistent with images in a and trajectories in b. (d) MSD of NPs motion before 7 s and calculated diffusion coefficients.

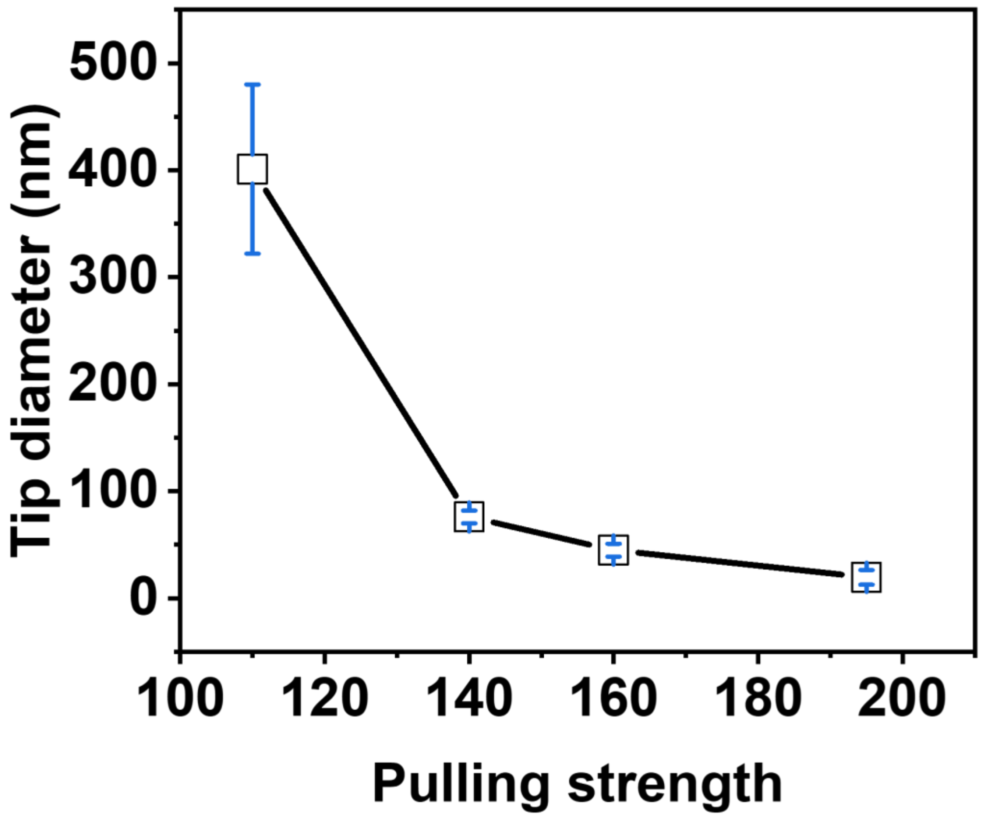

Fig. S3. The dependence of tip diameter on the pulling strength of the puller instrument. The pulling strength ranging from 0 to 255 is a parameter of the Laser-Based Micropipette Puller P-2000, and a change of one unit represents a change of 4 mA of current through the pull solenoid.

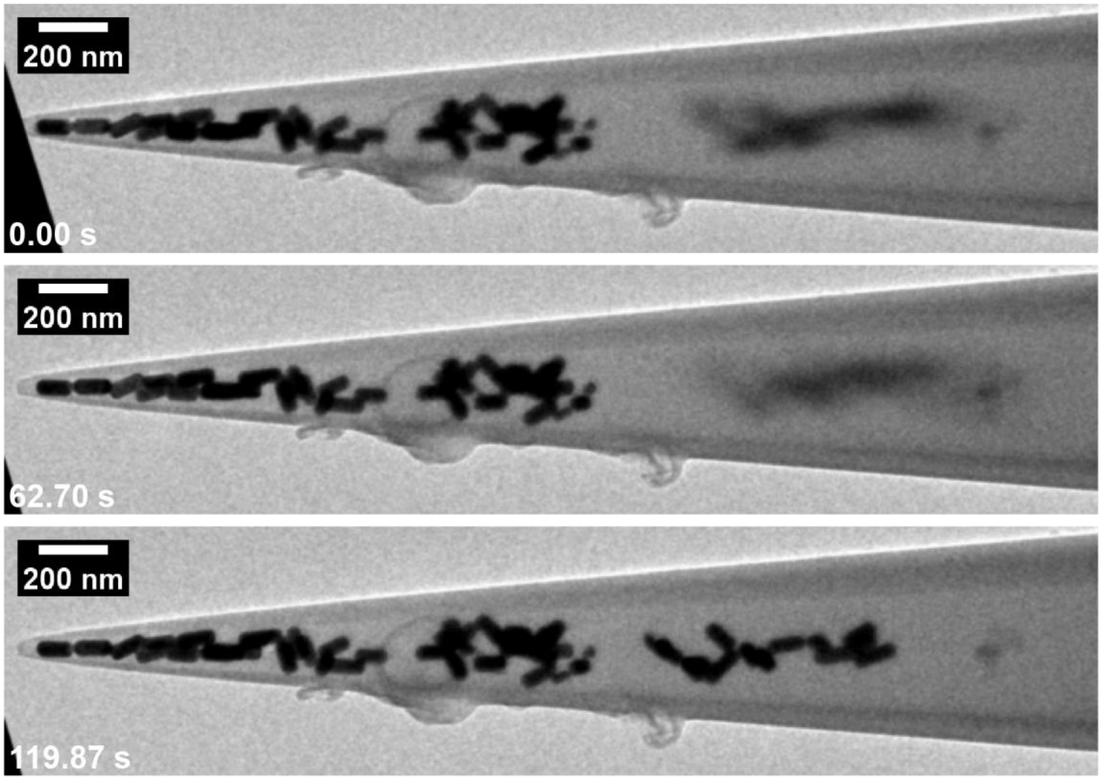

Fig. S4. Images show GNRs stuck in a narrow region but moving in the wide region of a nanopipette. Imaging condition: 200 kV, 2.4 $e^{-}/(Å^2·s)$.

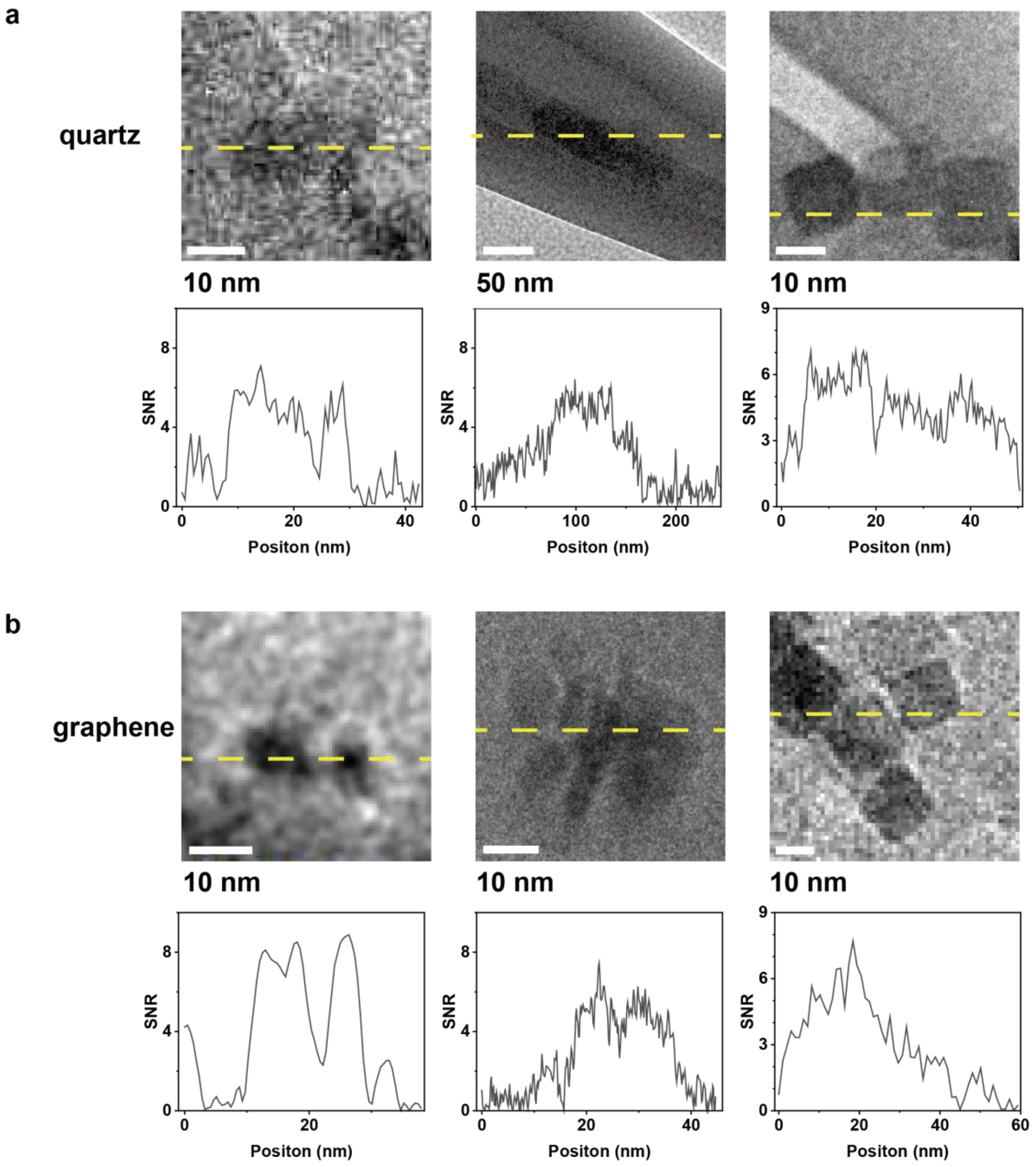

Fig. S5. Contrasts and SNR of samples obtained from (a) quartz nanopipette and (b) GLC liquid cells by electron microscopy. Sample solution (left to right): (a) 1 μM ssDNA; 100 mM LPA-OEG$_7$ in H_2O; Fe_3O_4 nanocube in *o*DCB; (b) ssDNA from reference 26; 100 mM LPA-OEG$_7$ in H_2O; Fe_3O_4 nanocube in chloroform. Imaging condition (left to right): (a) 200 kV, 10 $e^-/(Å^2{\cdot}s)$; 200 kV, 7 $e^-/(Å^2{\cdot}s)$; 200 kV, 53 $e^-/(Å^2{\cdot}s)$; (b) 80 kV, 2–5 $e^-/(Å^2{\cdot}s)$; 80 kV, 4 $e^-/(Å^2{\cdot}s)$; 80 kV, 3 $e^-/(Å^2{\cdot}s)$.

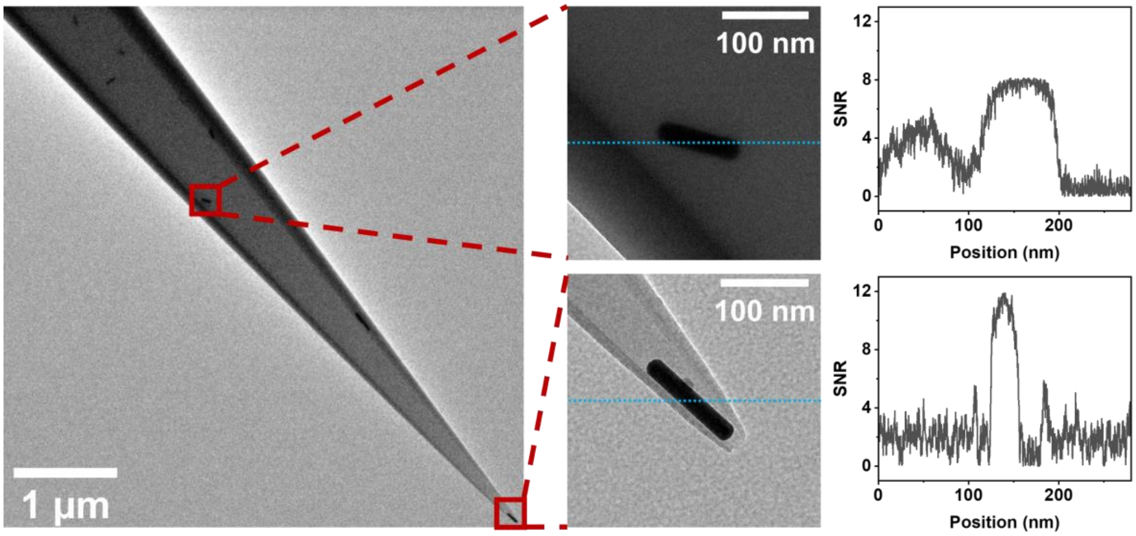

Fig. S6. Images of GNRs in the nanopipette. Solution: 0.24 nM GNRs and 1.0 mM CTAB in aqueous solution. The SNR of the nanopipette increases as its diameter decreases. Diameter: 800 nm for the top image and 20 nm for the bottom image. Imaging condition: 200 kV, < 1 $e^{-}/(Å^{2} \cdot s)$.

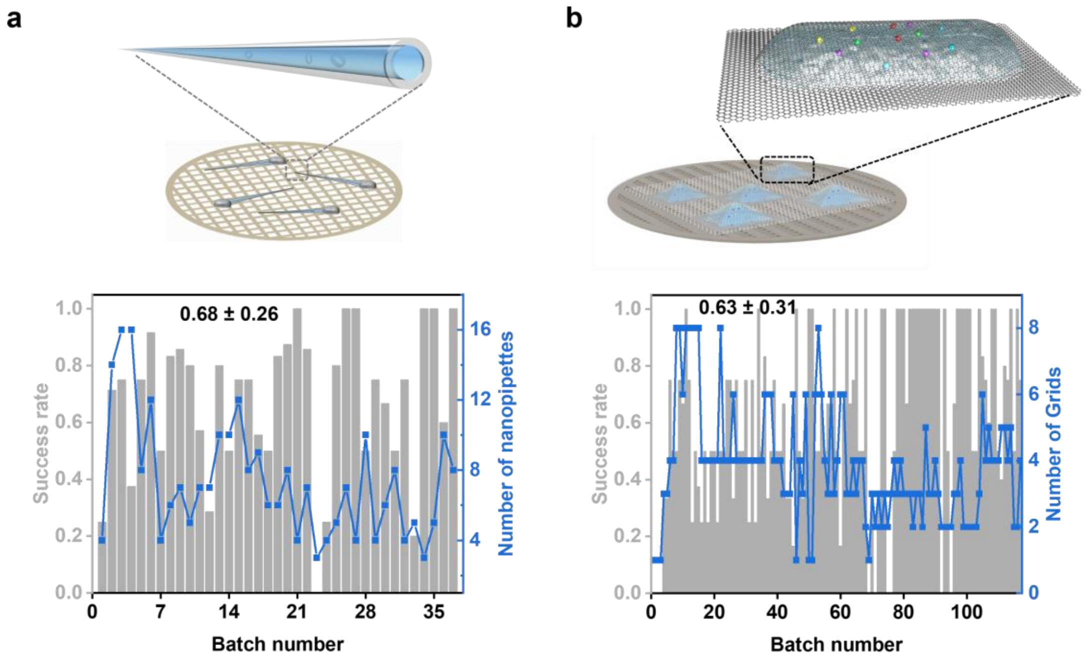

Fig. S7. Comparisons of success rate in finding viable liquids in quartz nanopipette (a) and GLCs (b). Imaging condition for quartz nanopipette: 200 kV, 0.1–450 $e^{-}/(Å^{2}\cdot s)$; for GLC: 80 kV, 1–100 $e^{-}/(Å^{2}\cdot s)$. The successful fabrication of GLCs (117 independent experiments, 454 grids, in which 283 grids contain 909 liquid cells) is defined as a cell with a diameter of 50 nm containing liquid. The successful rate of nanopipettes is defined as the number of nanopipettes that contain liquids divided by the total number of nanopipettes (37 independent experiments, 272 grids, in which 185 nanopipettes on 185 grids contained liquid). The lifetime of a liquid cell is defined as the duration from when the electron beam is turned on to when the liquid dries up.

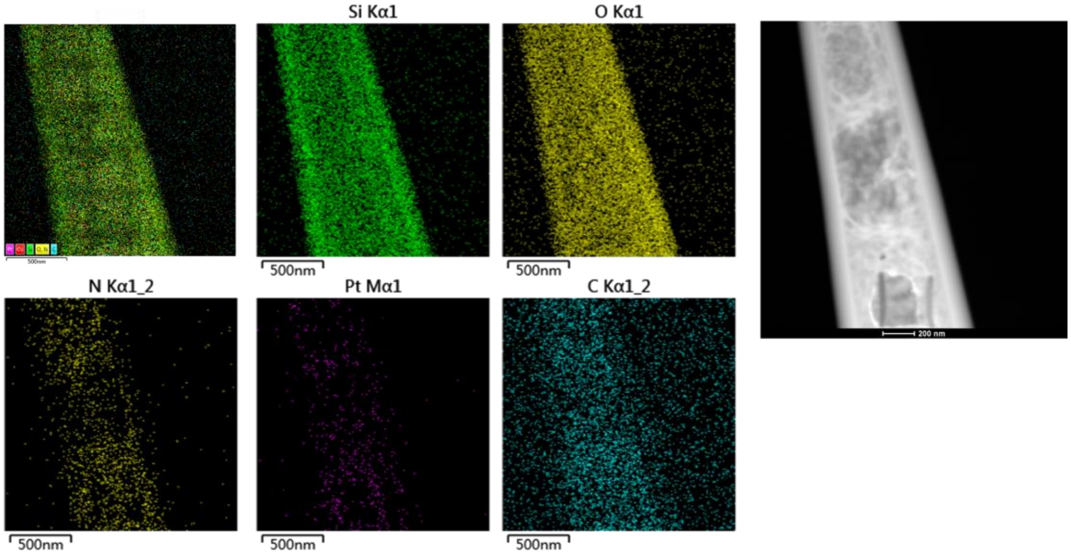

Fig. S8. Dissipation of gas confirmed by EDXS mapping of quartz nanopipette liquid cell containing platinum precursor solution (solvent: oleylamine/*o*DCB = 1:9) after the formation of droplets. As the oDCB evaporated, the chlorine signal was too low to be detected, while the nitrogen peak was still abundant, suggesting that the droplet was mainly oleylamine, and the oDCB had been dissipated, consistent with their boiling point and vapor pressure.

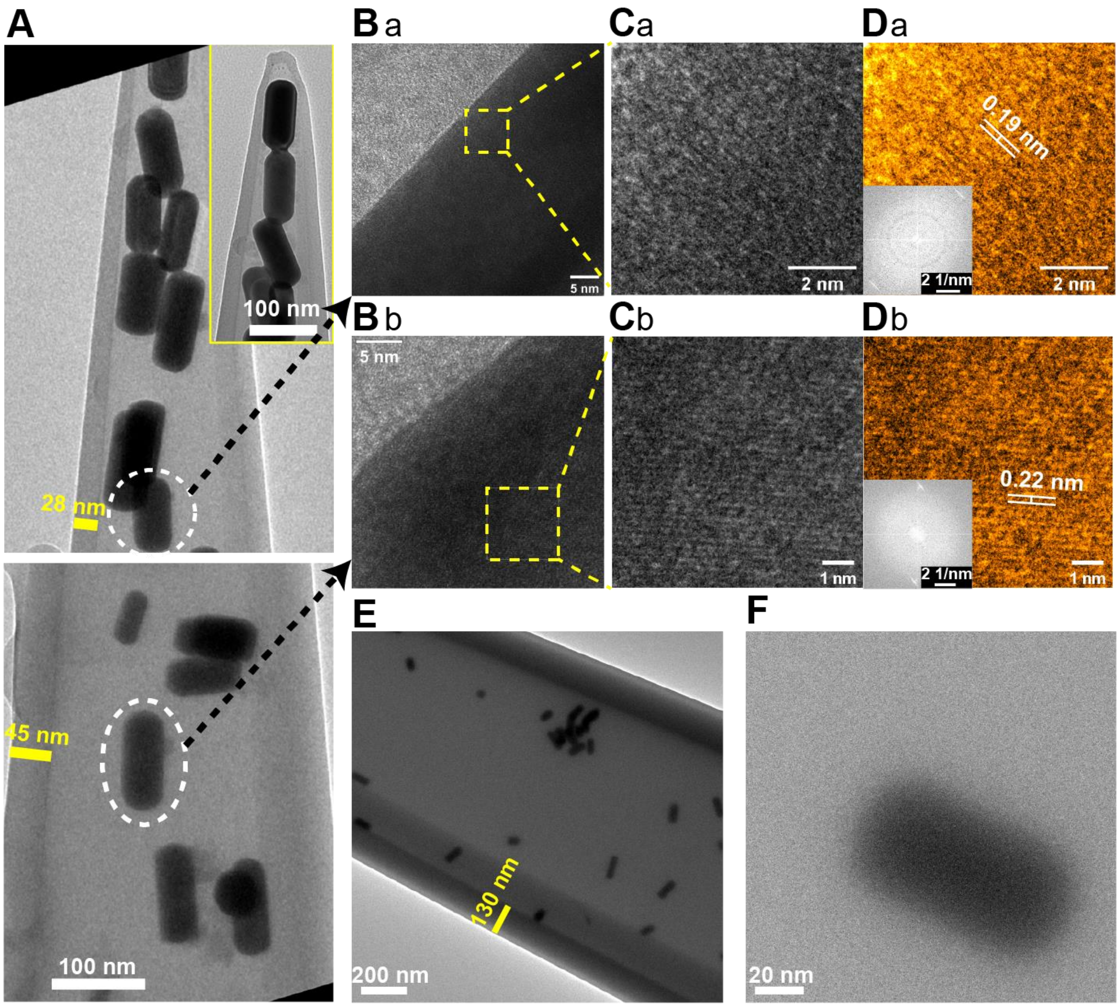

Fig. S9. LP-EM images of GNRs in a quartz nanopipette. Solution: 0.24 nM GNRs and 1.0 mM CTAB in aqueous solution. (A) Low-magnification (30kX) images were taken on JEM-2100 Plus HC. Imaging conditions: 200 kV, 5.8 $e^{-}/(Å^{2}\cdot s)$. Images show GNRs in nanopipette with wall thicknesses of (a) 28 nm and (b) 45 nm, respectively. Zoomed shows the liquid retained because the tip has been sealed. (B–D) High-resolution images taken on JEM-F200. Imaging conditions: 200 kV, 2693 $e^{-}/(Å^{2}\cdot s)$ for (a); 200 kV, 1594 $e^{-}/(Å^{2}\cdot s)$ for (b). (B) Zoom in on images in A. (C) Zoom in on images in B. (D) Corresponding pseudo-color images in C show lattice stripe spacing d = 0.19 nm for a and 0.22 nm for b. Zoomed shows Fast Fourier Transformation (FFT) analysis of images in C. (E, F) Images showing no lattice structure at a wall thickness of 130 nm with particle motion blur. Images taken on JEM-F200. (E) Low magnification (25kX) image. Imaging condition: 200 kV, 6.4 $e^{-}/(Å^{2}\cdot s)$. (F) High magnification (300kX) image. Imaging condition: 200 kV, 310 $e^{-}/(Å^{2}\cdot s)$.

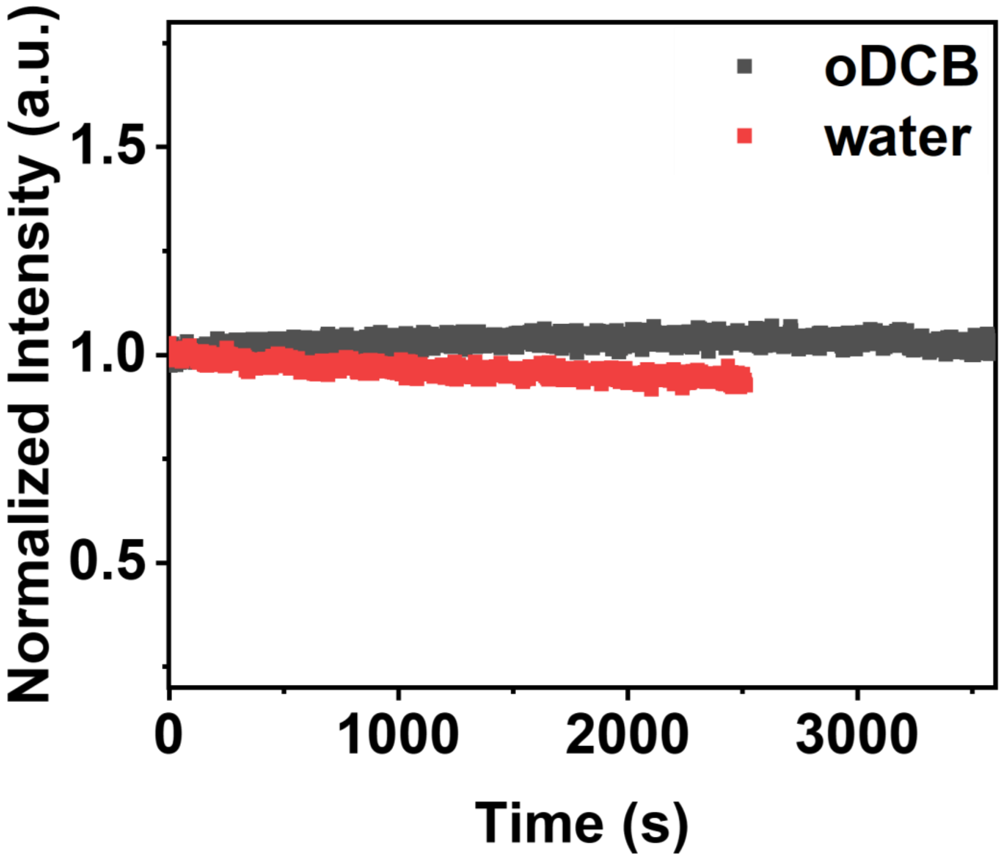

Fig. S10. Intensity fluctuations during imaging for quartz pipette liquid cell containing water (red), *o*DCB (black). We stopped imaging at 3600 s and 2500 s, and the liquid had not dried yet. Imaging condition: 200 kV, $< 1\ e^{-}/(Å^{2}\cdot s)$.

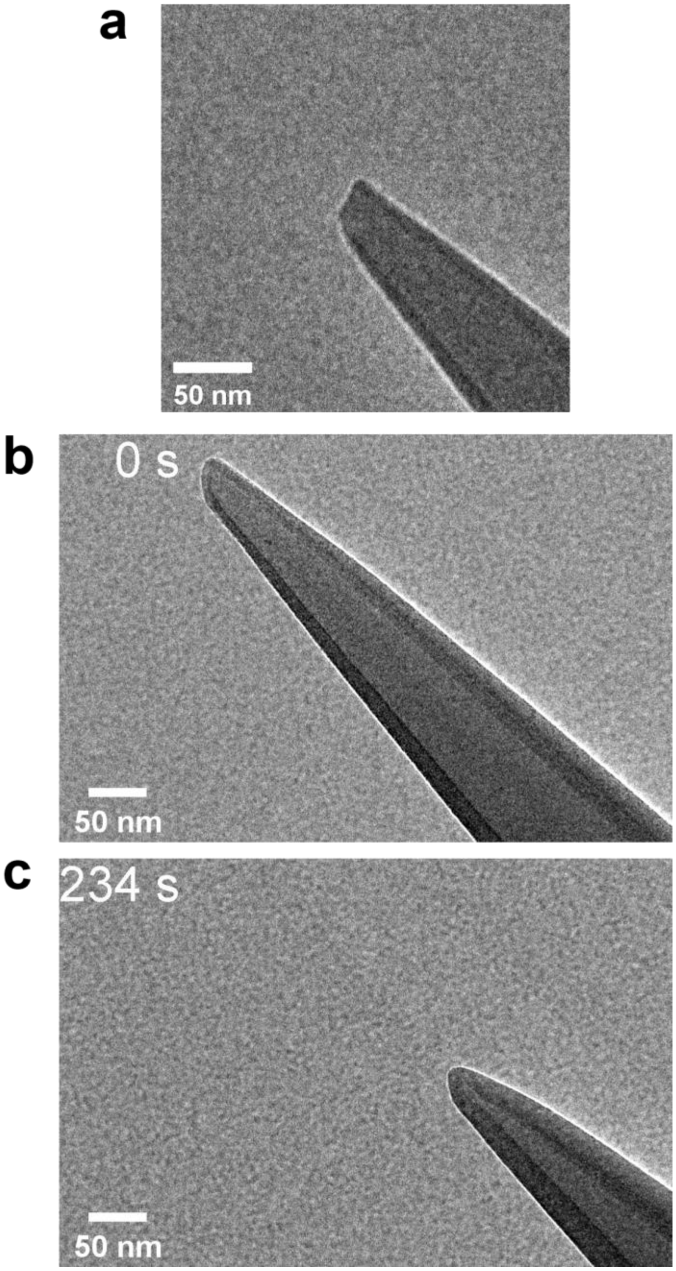

Fig. S11. (a) Initial state of a nanopipette. (b, c) Closure and deformation of the tip. Imaging condition: 200 kV, 22 $e^{-}/(Å^2 \cdot s)$.

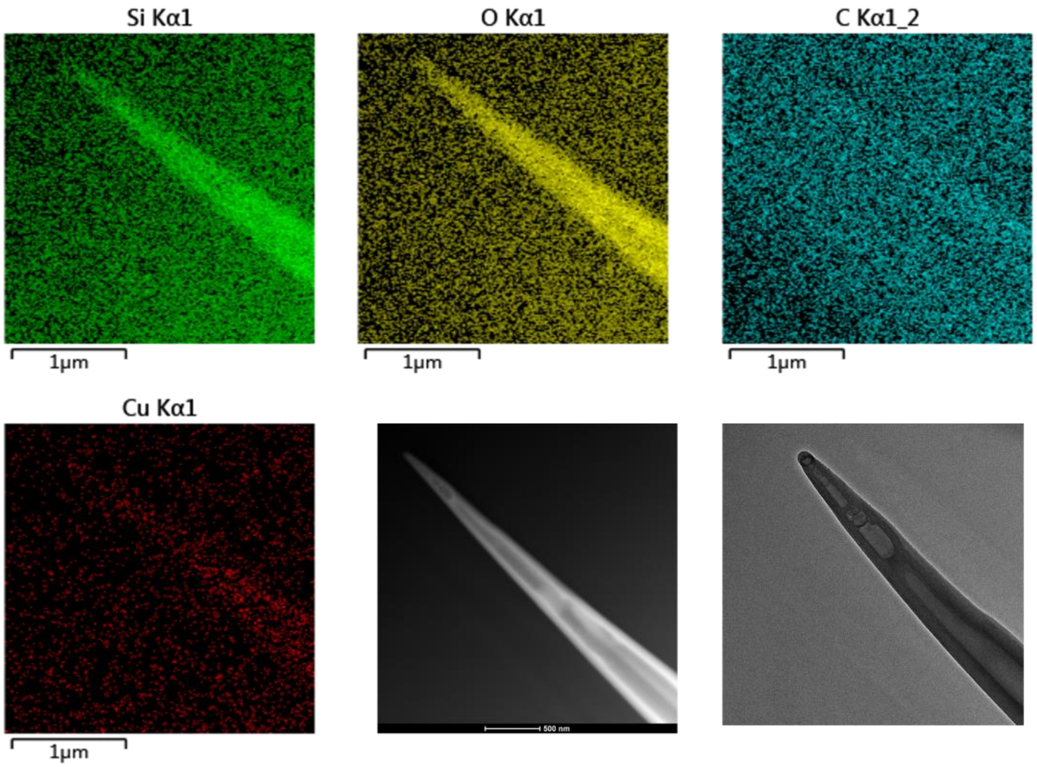

Fig. S12. EDXS mapping confirms the successful coating of a thin layer of conductive carbon film (~5 nm) on the quartz nanopipette.

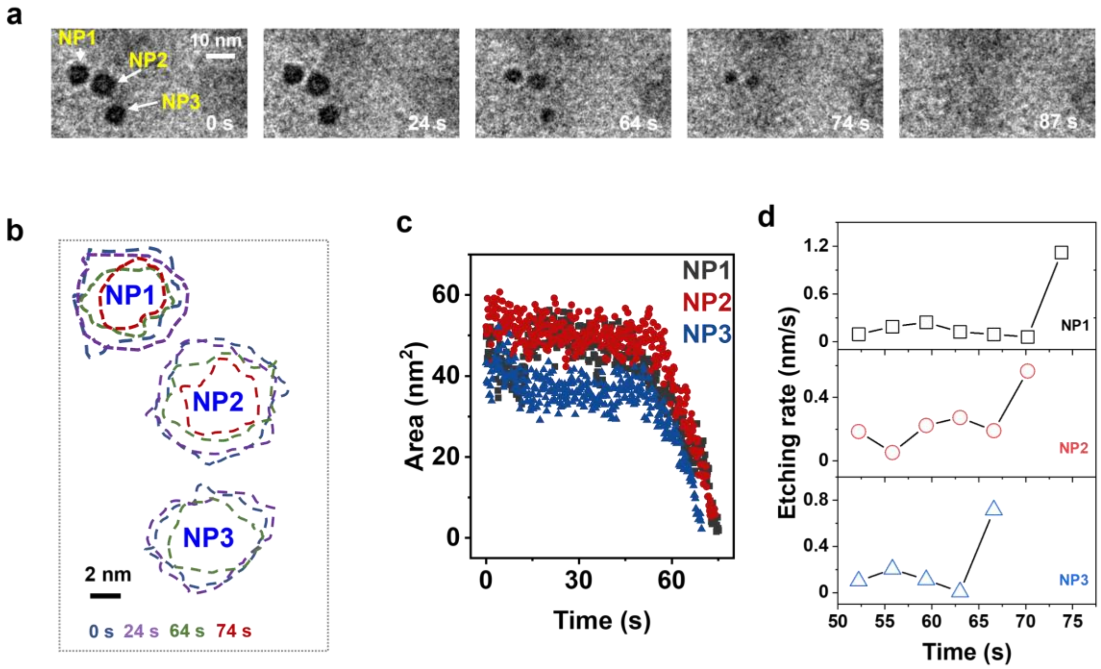

Fig. S13. Nanoparticle etching observed in a nanopipette. (a) The beam-induced etching process of Au nanoparticles. Dose rate: 230 $e^{-}/(Å^{2}\cdot s)$, scale bar: 10 nm. (b) Time-lapsed contour plots of individual particles. The scale bar is 2 nm. 0 s, 24 s, 64 s, and 74 s are blue, purple, green, and red. (c) Change of Au nanoparticle size plotted as a function of time during etching. (d) Time-dependent etching rate for individual nanoparticles.

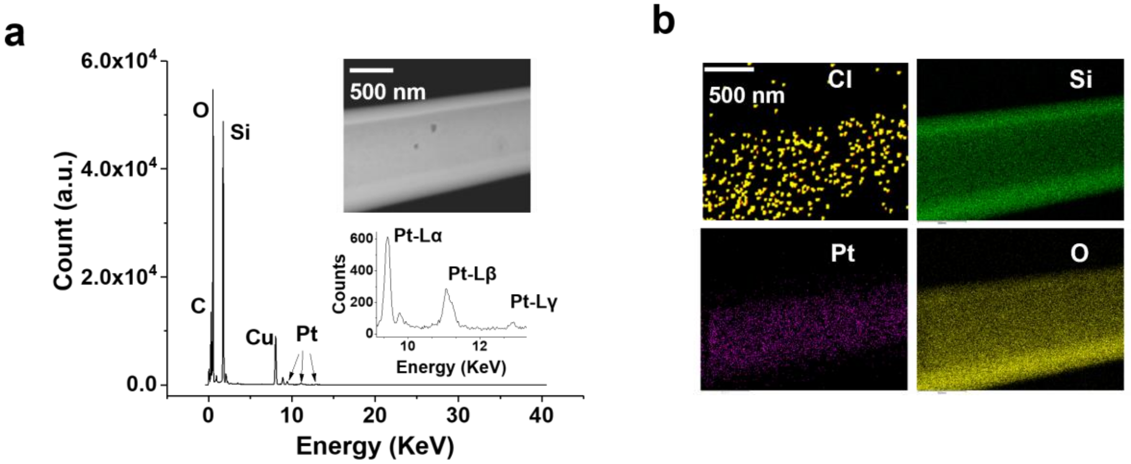

Fig. S14. EDXS experiments confirmed the successful encapsulation of platinum nanoparticle precursor solution in *o*DCB. (a) EDXS spectra. The inset is the image of the mapped area (top) and zoom-in spectrum for Pt peaks (bottom). Platinum, chloride signal from the solution, and the high abundance of oxygen and silicon signals came from quartz. The carbon and copper signals came from the EM grids, the supporting carbon film, and the copper mesh, respectively. (b) The corresponding EDXS images.

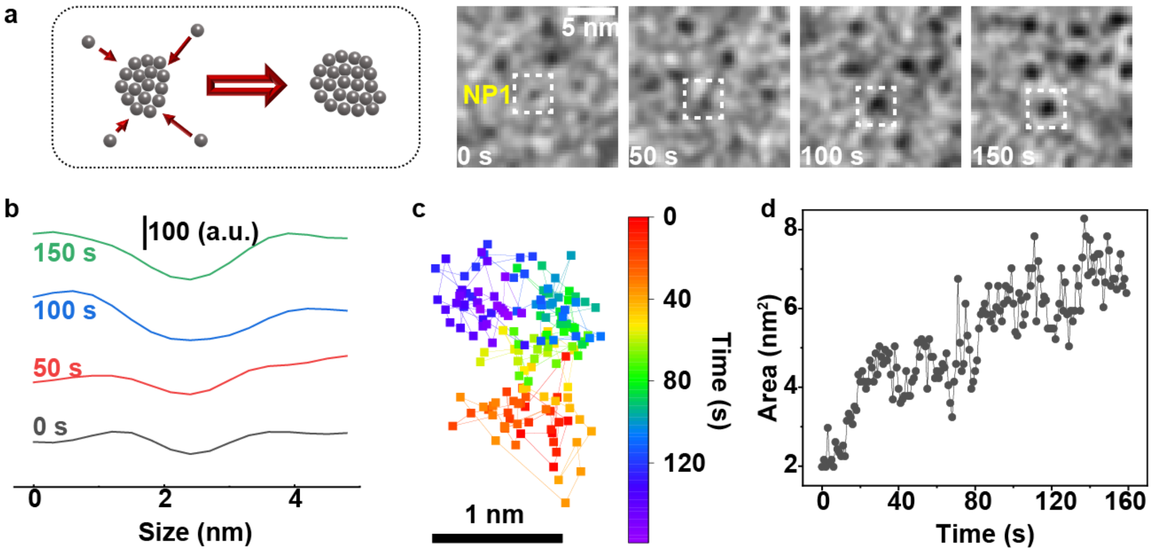

Fig. S15. Nanoparticle growth by monomer attachment mechanism observed in a quartz nanopipette. (a) Schematic diagram and time-lapsed electron micrographs of Pt nanoparticles in its precursor solution. Dose rate: 167 $e^{-}/(Å^{2}\cdot s)$. (b) Dependence of intensity on NP1 position of coordinate. (c) Particle trajectory of NP1. The color code indicates the time. (d) Change of NP1 sizes plotted as a function of time.

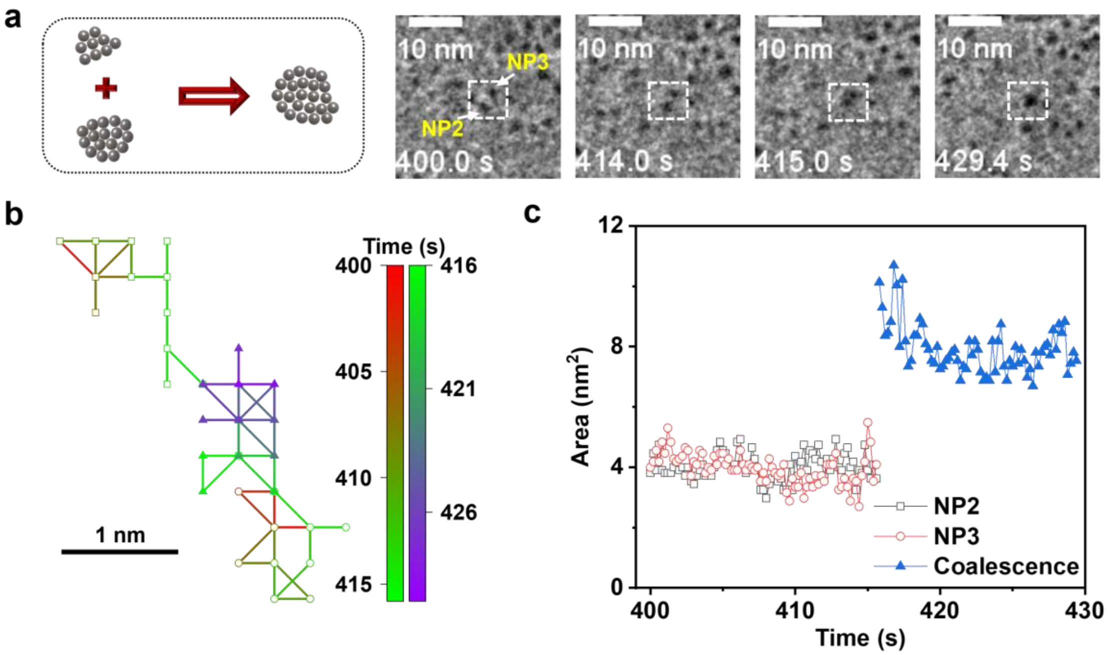

Fig. S16. Nanoparticle growth by coalescence mechanism observed in a quartz nanopipette. (a) Schematic diagram and time-lapsed electron micrographs of Pt nanoparticles. Dose rate: 160 $e^{-}/(Å^2 \cdot s)$. (b) Particle trajectories of NP2 and NP3 before and after coalescence. The color code indicates the time. The tracking precision is inherently limited by the pixel size and spatial resolution of our experimental condition. (c) Change of NP2 and NP3 sizes plotted as a function of time.

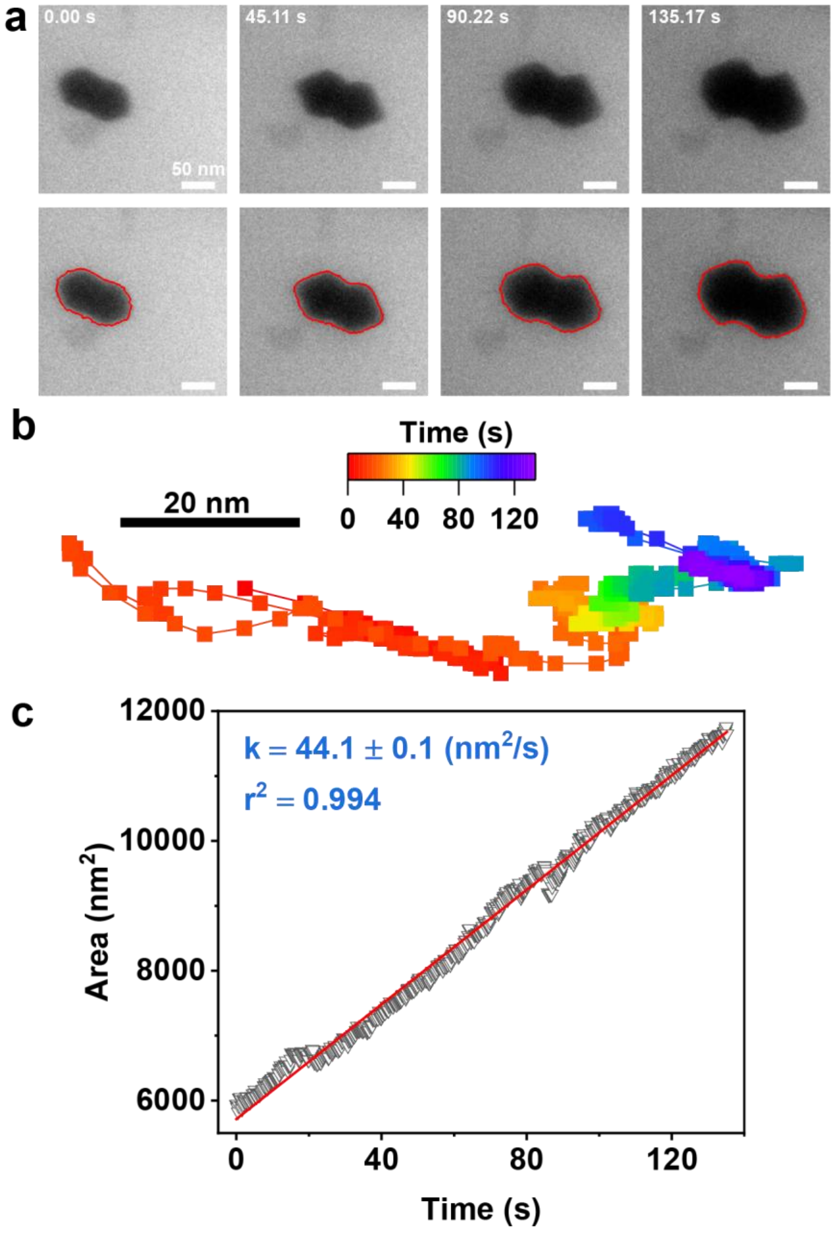

Fig. S17. (a) (Top) LP-EM images show a GNR that grew up to GNA. (Bottom) Corresponding images with highlights of the particle periphery. Scale bar: 50 nm. Imaging condition: 200 kV, 27 $e^{-}/(Å^{2}\cdot s)$. Images were taken on JEM-2100 Plus HC. (b) Time-lapsed particle trajectory. The color bar represents time. (c) Dependence of particle projected area as a function of time. The area increases linearly with time.

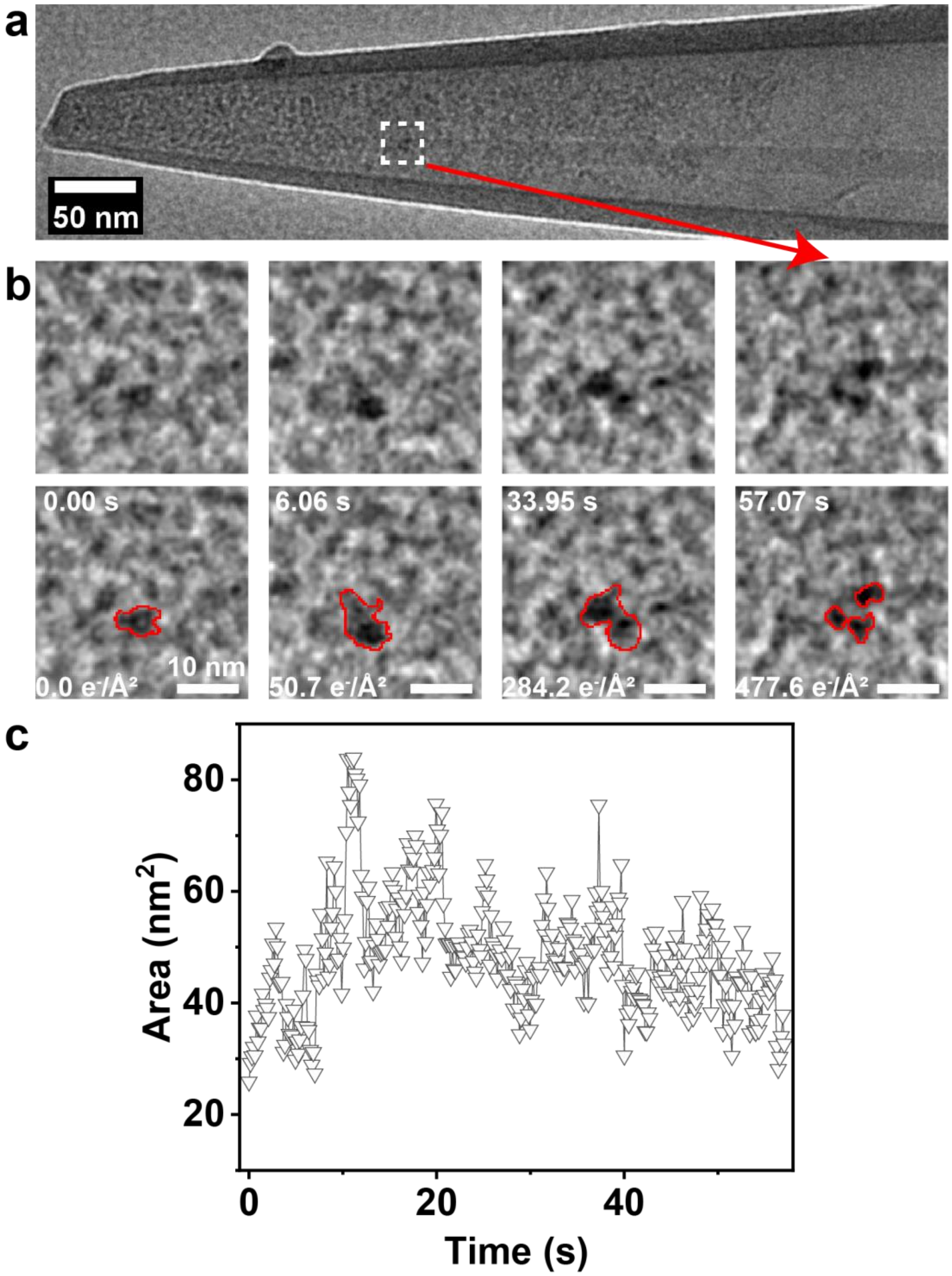

Fig. S18. ssDNA dynamics observed in nanopipettes. (a) LP-EM image shows ssDNA in a nanopipette with a thin wall. Solution: 10 μM ssDNA in D_2O. Imaging condition: 200 kV, 8.4 $e^{-}/(Å^2·s)$. Images were taken on JEM-2100 Plus HC. (b) Time-lapsed electron micrographs captured the conformational dynamics of the ssDNA molecule. (c) The projected area of the ssDNA molecule is plotted as a function of time.

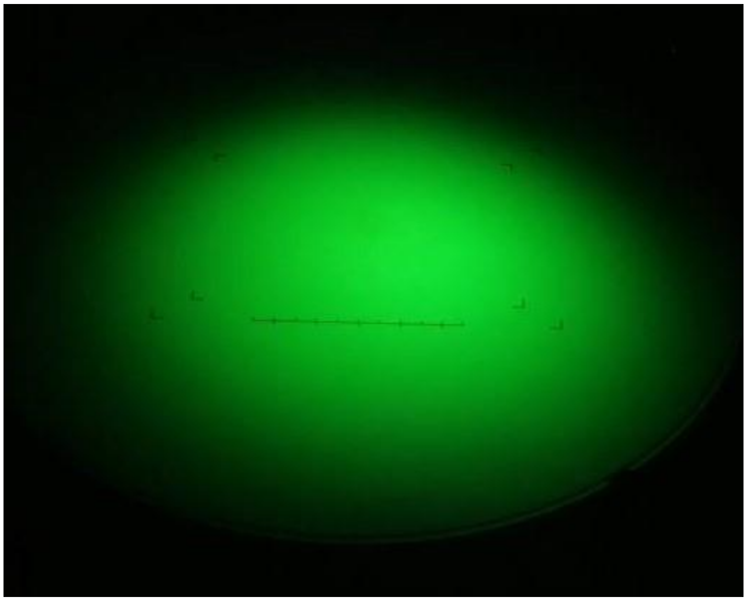

Fig. S19. Electron beam with blurred edges on the phosphor screen of JEM-2100 Plus HC.

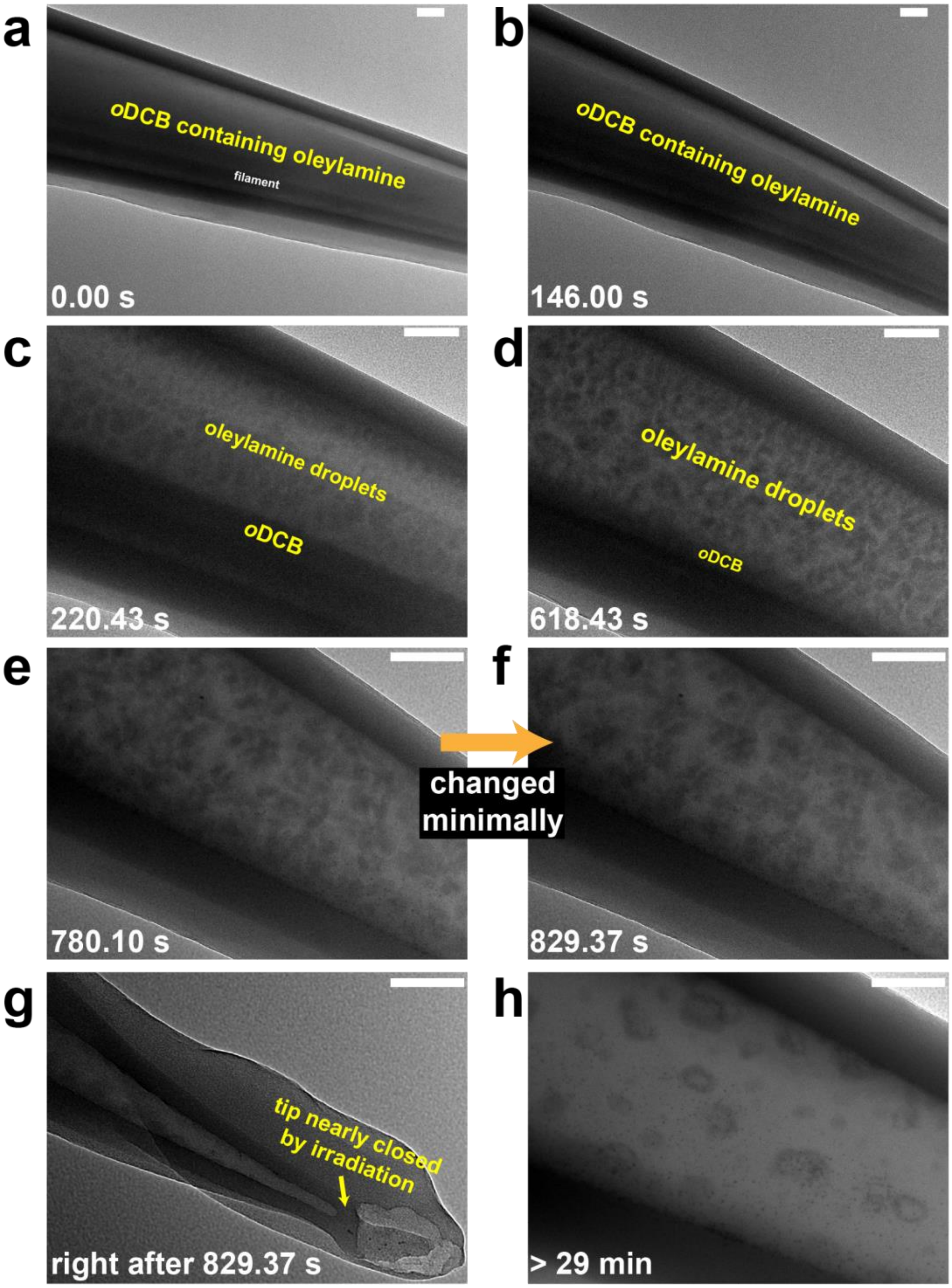

Fig. S20. A Longer time is needed to form droplets from evaporation under JEM-2100 Plus HC. Solution: Pt precursor solution. Scale bars: 100 nm. Imaging condition: 200 kV. (a–d) *o*DCB in the nanopipette (diameter of 200–420 nm) took 618 s to gradually fade away and form droplets. Dose rate: 38–175 $e^{-}/(Å^{2}\cdot s)$. (e–f) Moving droplets ceased motion (780–829 s). Dose rate: 480 $e^{-}/(Å^{2}\cdot s)$. (g) The tip was closed after 829 s. (h) Droplets took nearly 29 min to evaporate completely, forming ring-like composites.

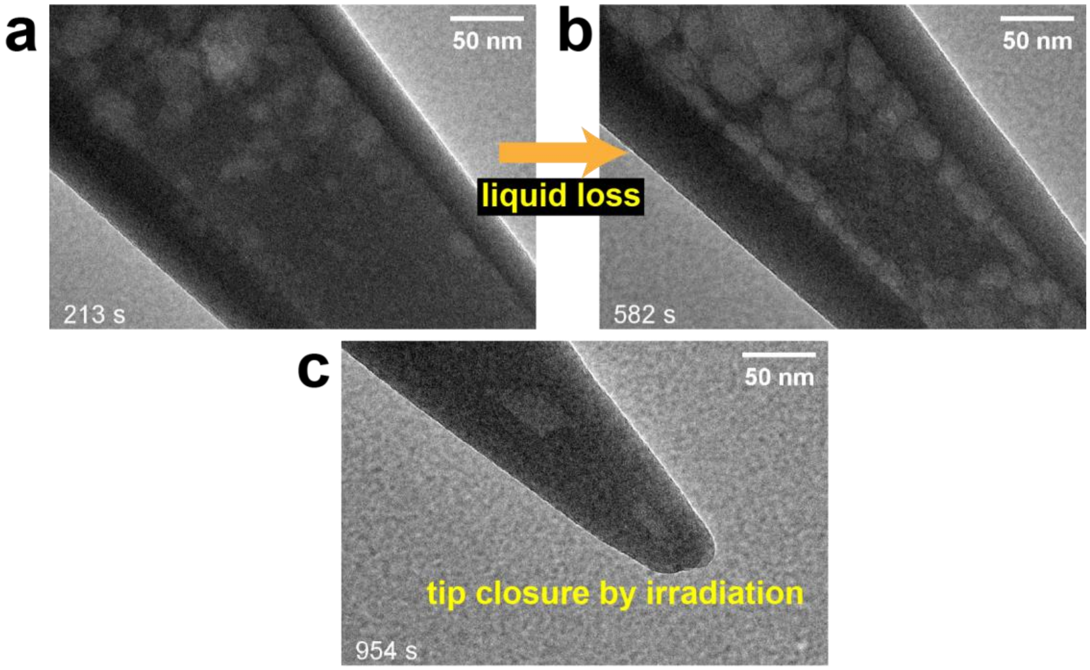

Fig. S21. Nanopipette with a clogged tip is unfavorable for observing droplet formation. Solution: oleylamine/*o*DCB. Imaging condition: T20, 200 kV, 46 $e^{-}/(Å^{2}\cdot s)$. Time-lapse images of nanopipette showing liquid loss (a and b), with tip sealed by electron-beam irradiation (c).

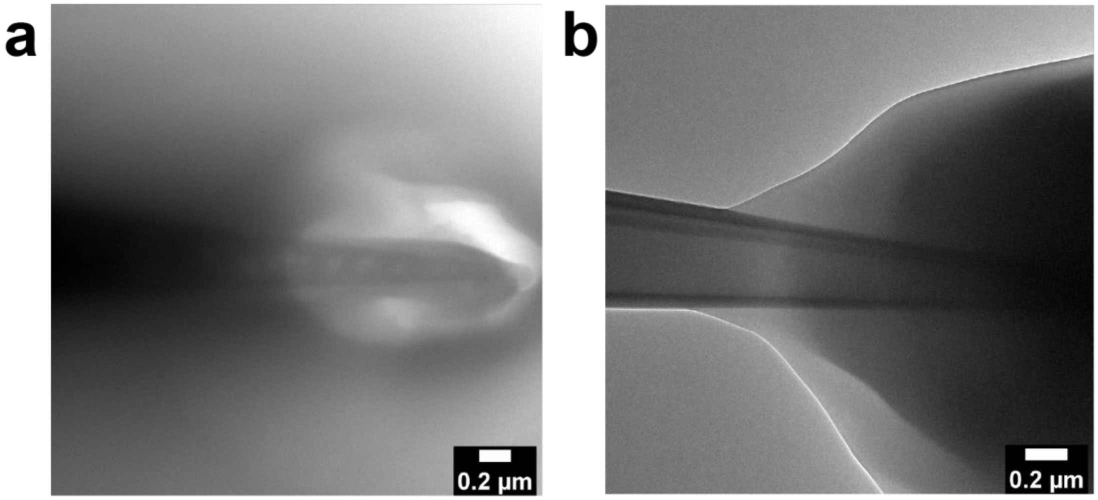

Fig. S22. Nanopipette with a clogged tip is unfavorable for observing droplet formation. (a) The tip of a nanopipette is covered by vacuum grease. (b) The tip of a nanopipette is covered by a big droplet. Imaging condition: JEM-2100 Plus HC, 200 kV, 13 $e^{-}/(\text{Å}^2 \cdot s)$ for (a) and 5 $e^{-}/(\text{Å}^2 \cdot s)$ for (b).

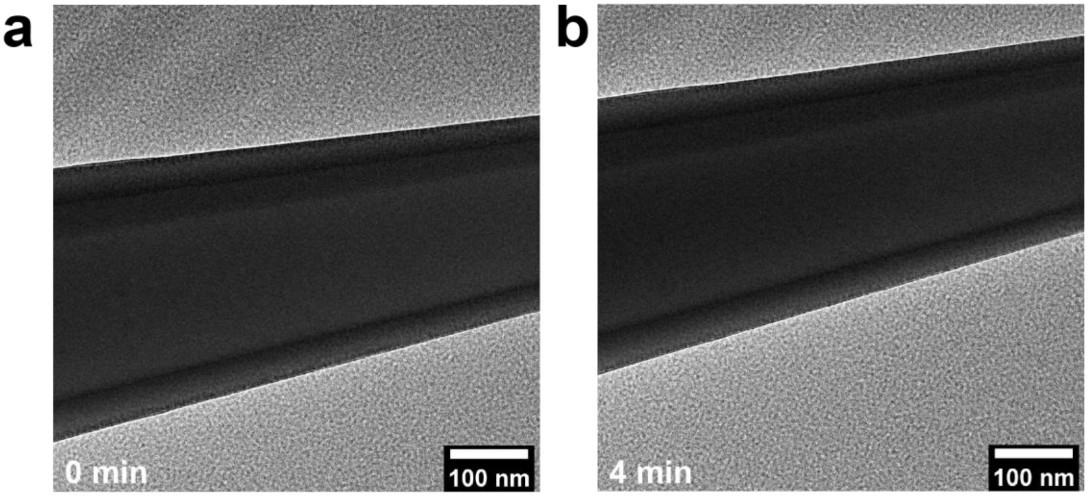

Fig. S23. Liquid remains filled for at least 4 min without forming thin liquid film and nanodroplets. Experimental condition: F20, 200 kV, 90–100 $e^{-}/(Å^{2}\cdot s)$. Solution: Pt precursor solution.

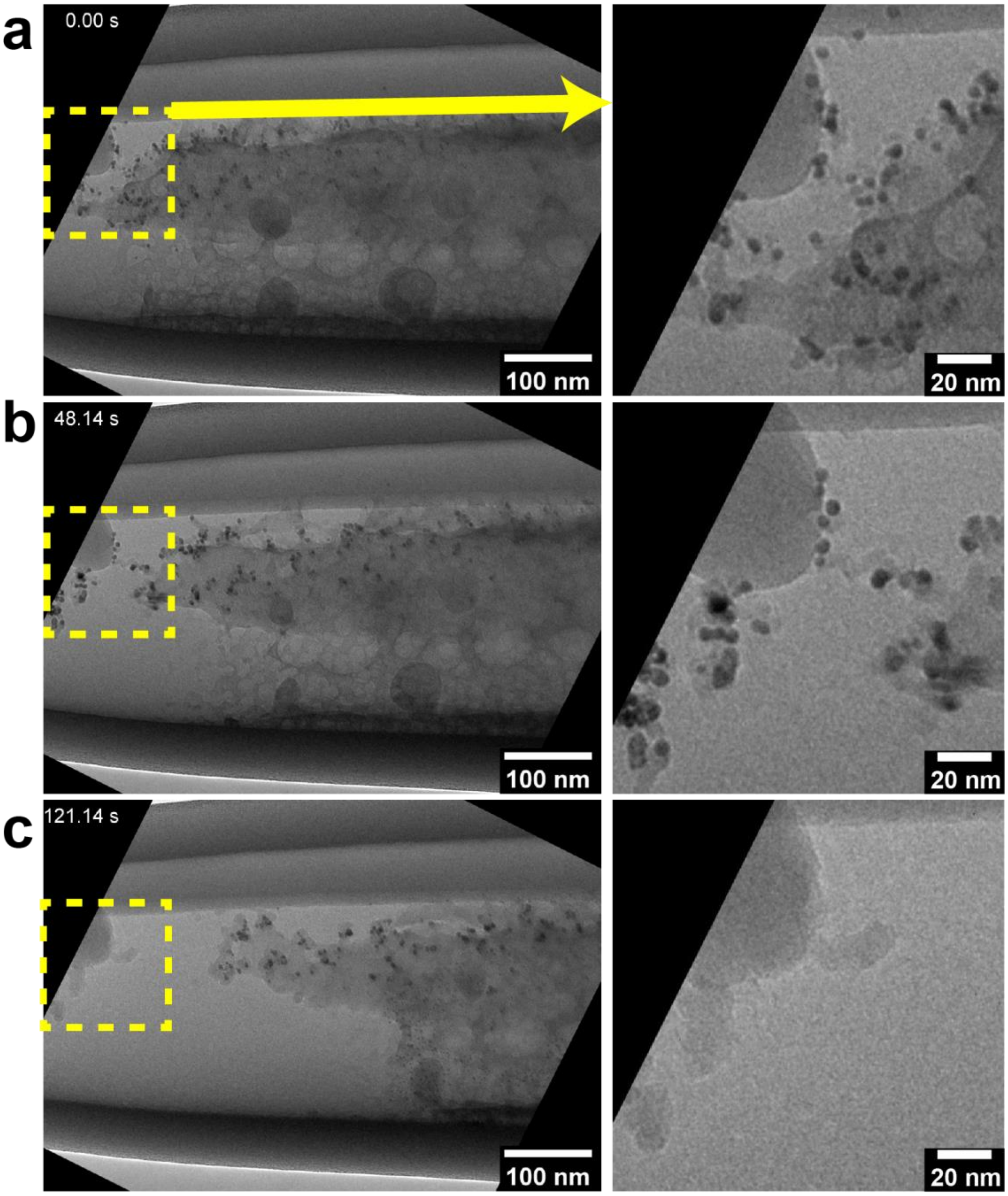

Fig. S24. Solvent evaporated but failed to form nanodroplets, leaving behind a gel-like substance. Time-lapse images in a, b, and c. The corresponding zoom-in images are on the right for each panel. Experimental condition: JEM-2100 Plus HC, 200 kV, 187 $e^{-}/(Å^{2}\cdot s)$. Solution: Pt(acetylacetonate)$_2$ in oleylamine/DCM.

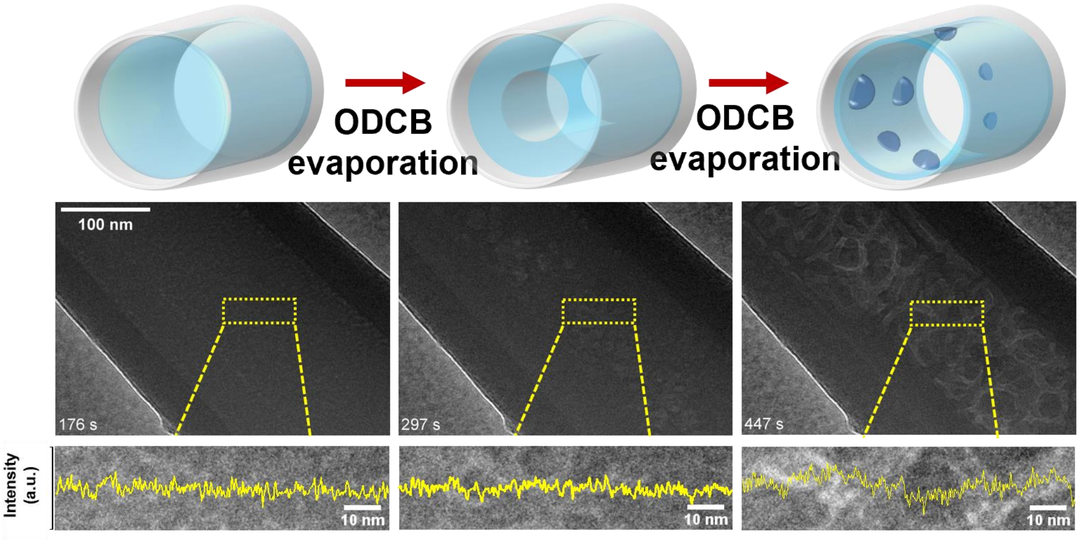

Fig. S25. The fate of liquid in the nanopipette and the formation of nanodroplets due to liquid dewetting upon evaporation. Sample solution: Pt precursor solution. Imaging condition: 200 kV, dose rate 180 $e^-/(Å^2 \cdot s)$. The scale bar is 100 nm. Time zero denotes when the electron beam is on. Schematic depiction (top) and time-lapsed liquid-phase electron microscopy images (middle) of how liquid evaporates and dewets to form droplets in a quartz nanopipette and the zoom-in images (bottom). Insets are the corresponding intensity analysis.

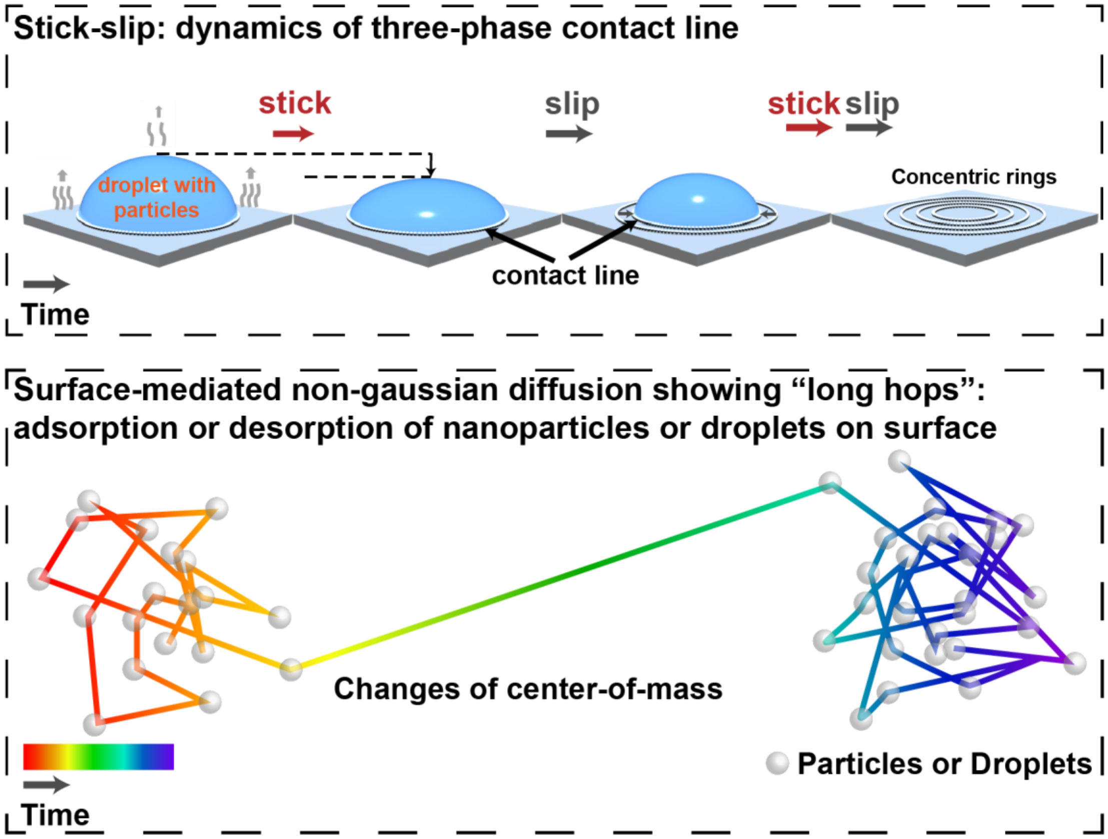

Fig. S26. Schematic illustrations of the "stick-slip" of the contact line of nanodroplets and surface-mediated "long hops" of the center-of-mass of nanoobjects, including molecules, particles, and nanodroplets.

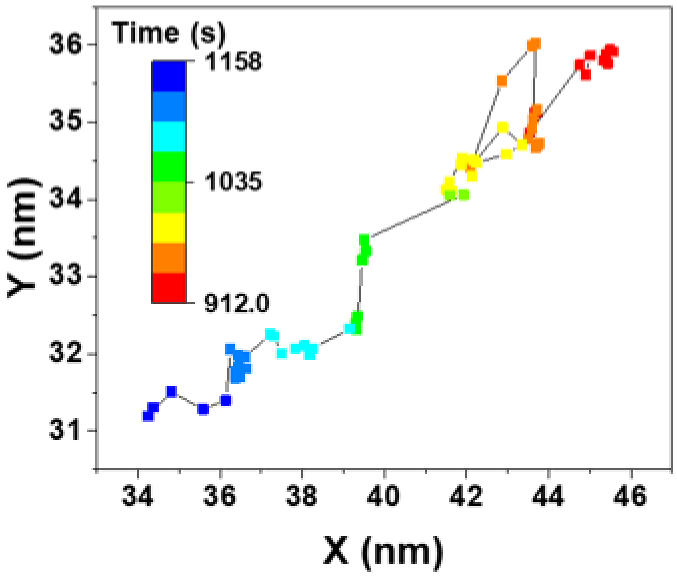

Fig. S27. A plot of time-lapsed changes of the center-of-mass of the nanodroplet. Color code indicates the time, from the beginning of the process at 912 s in red to the end at 1158 s in blue.

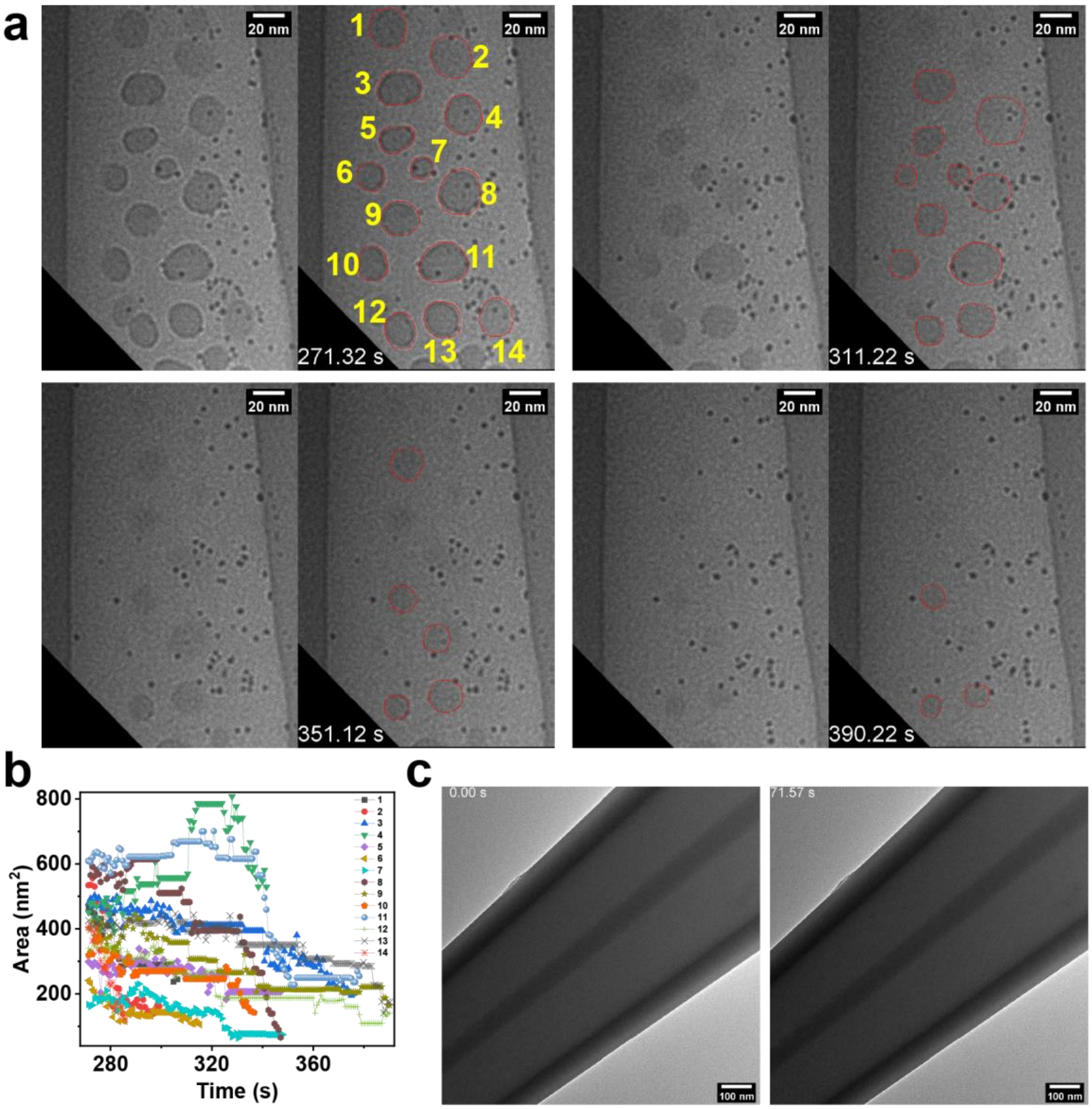

Fig. S28. The higher electron beam dose rate allows us to observe more "stick-slip" events of nanodroplet contact lines than the permanently pinning events. (a) LP-EM images capture droplet evaporation in a nanopipette (tip diameter: 20 nm). Solution: Pt precursor solution. Scale bar: 20 nm. Imaging condition: F20, 200 kV, 450–550 $e^{-}/(Å^{2}\cdot s)$. (b) Quantification of droplet area as a function of time. Almost all droplets are “stick-slip” type, showing both plateaus and decays. (c) No evaporation of liquid was observed for continuous imaging of 71.57 seconds at a low dose rate (tip diameter: 20 nm) with JEM-2100 Plus HC. Imaging condition: 200 kV, 21 $e^{-}/(Å^{2}\cdot s)$.

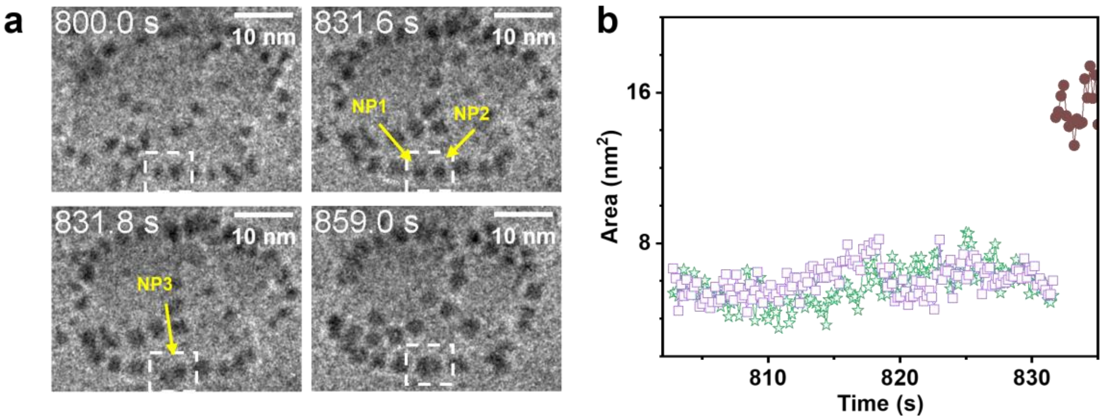

Fig. S29. Coalescence of nanoparticles in a nano coffee ring. (a) Time-lapsed electron micrographs of platinum nanoparticles. Imaging condition: F20, 200 kV, dose rate: 907 $e^{-}/(Å^{2}\cdot s)$. (b) The size of NP1 and NP2, as well as after their coalescence (NP3), are plotted as a function of time. NP1 in green stars, NP2 in purple squares, and NP3 in brown circles.

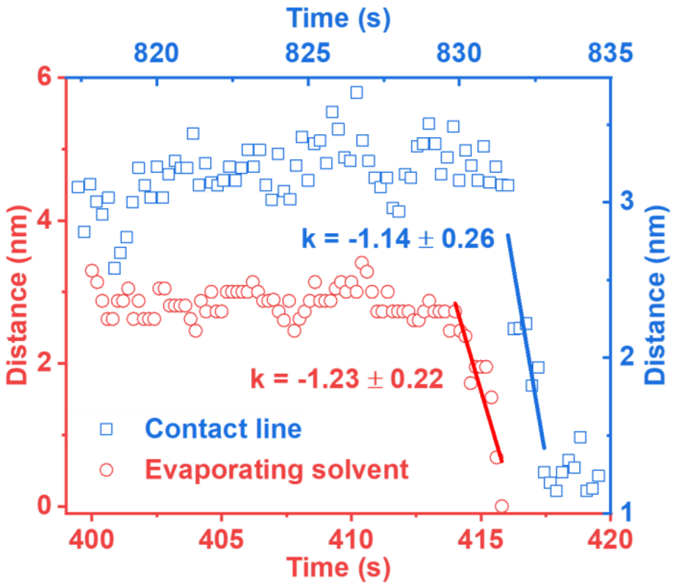

Fig. S30. Comparison of the coalescence event at the contact line of a nanodroplet to that outside of the droplet (evaporating solvent). The distance of two adjacent particles that undergo coalescence event was plotted as a function of time. The coalescence rates quantified by the slope k are similar.

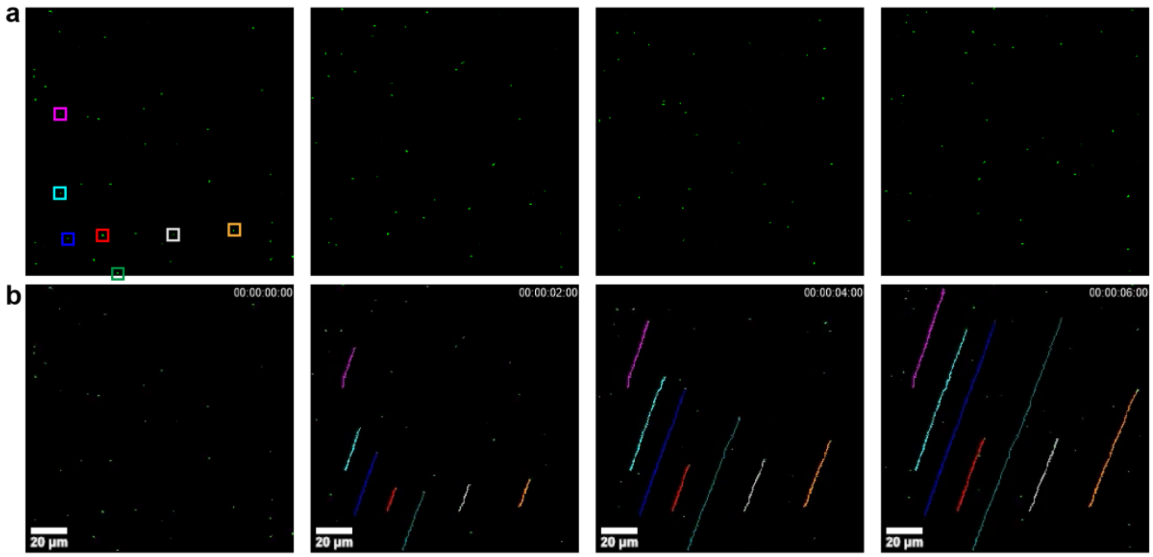

Fig. S31. Formation of micro coffee ring imaged by confocal microscopy. (a) Carboxylate-modified polystyrene fluorescent latex beads (green points, also highlighted with squares to show the representative images) in the small region (approximately 150 × 150 µm) of an evaporating macroscale droplet (Diameter: ~5 mm) moved with the capillary flow towards the upper right of the image. (b) Trajectories of the beads at different times.

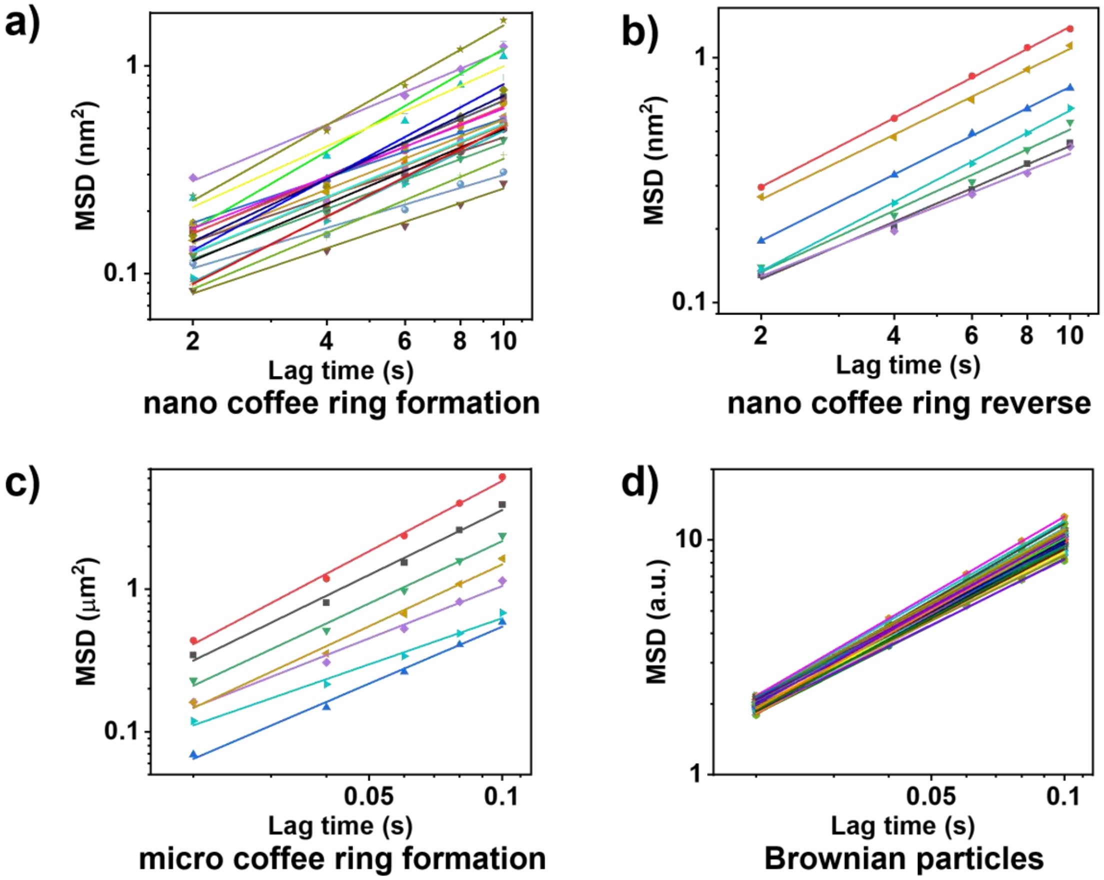

Fig. S32. Mean square displacement (MSD) of particle motion of (a) formation of a nano coffee ring, (b) reverse of nano coffee ring, (c) formation of a micro coffee ring, and (d) Brownian particles.

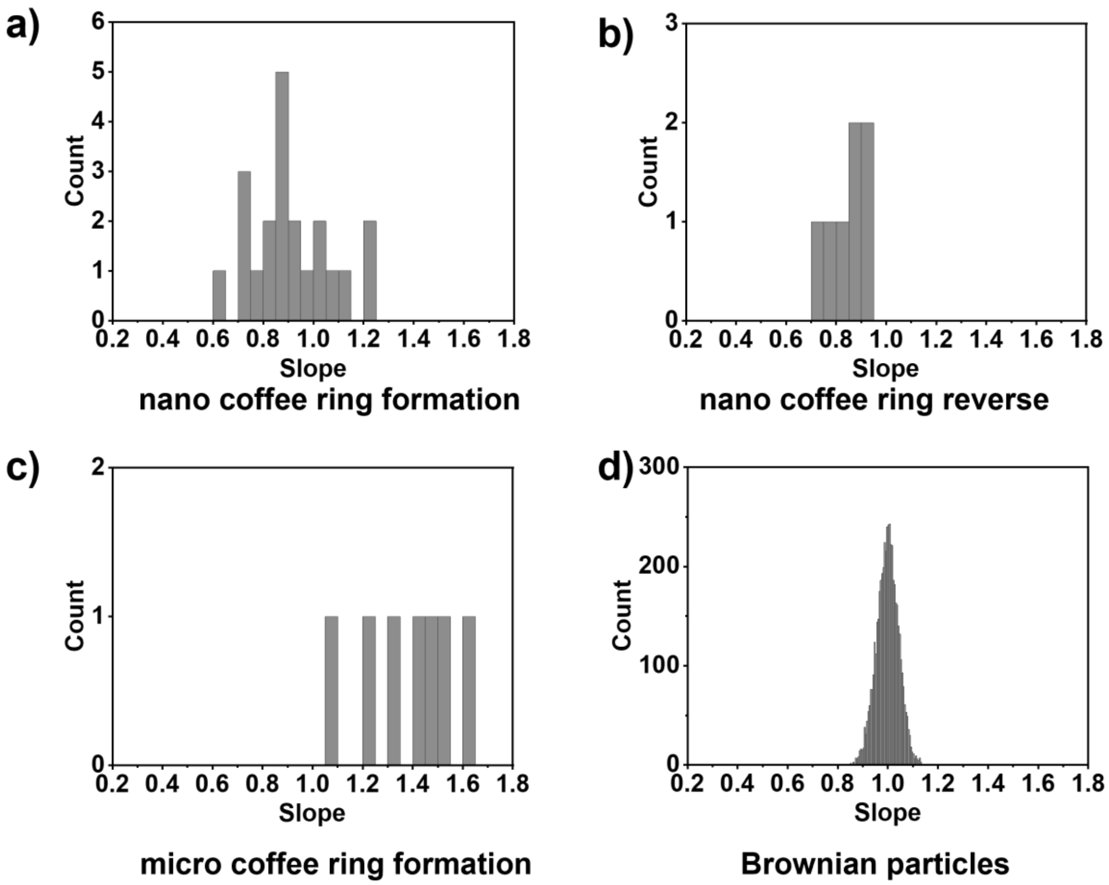

Fig. S33. Distribution of slopes from MSD calculation of (a) formation of a nano coffee ring, (b) reverse of nano coffee ring, (c) formation of a micro coffee ring, and (d) Brownian particles.

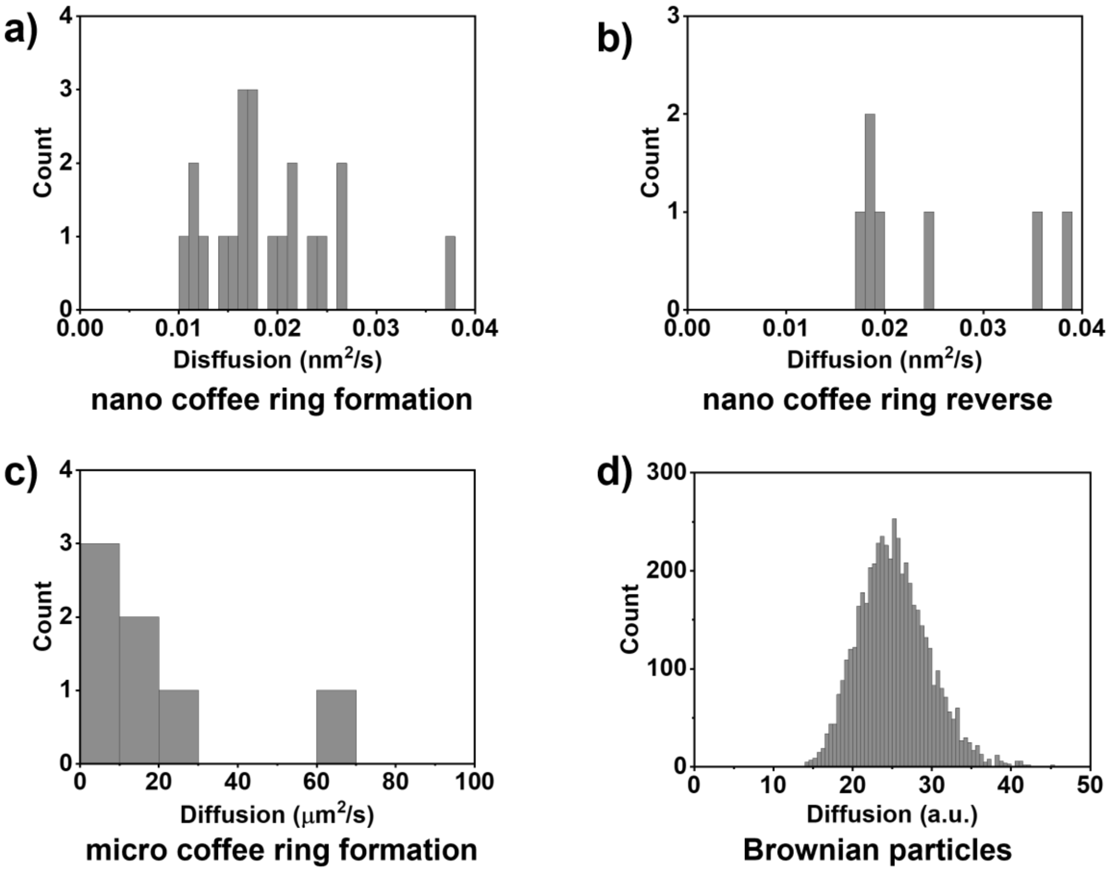

Fig. S34. Distribution of diffusion coefficients from MSD calculation of (a) formation of a nano coffee ring, (b) reverse of nano coffee ring, (c) formation of a micro coffee ring, and (d) Brownian particles.

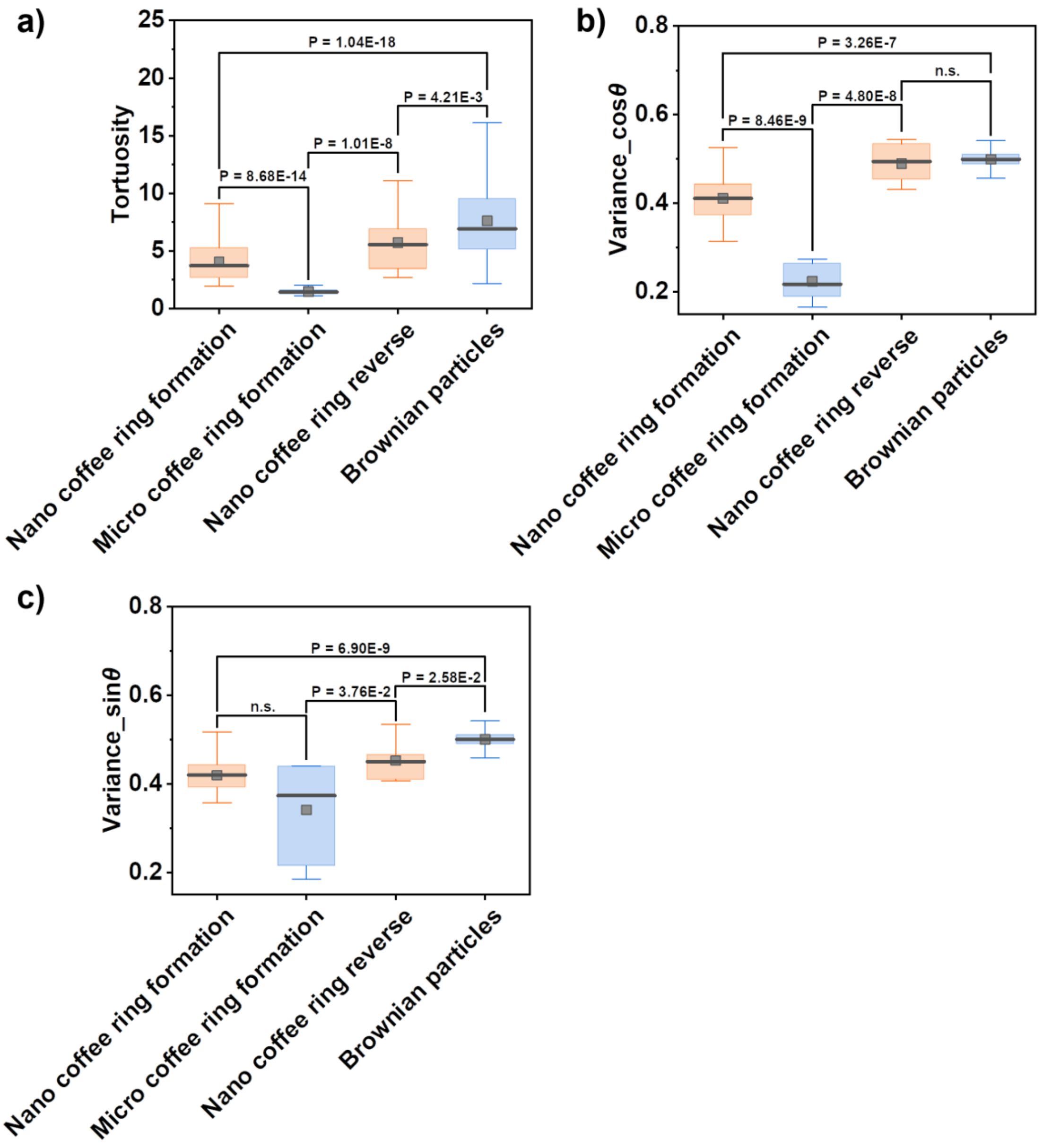

Fig. S35. (a) Tortuosity of nano coffee ring formation, macro coffee ring, nano coffee ring reverse, and Brownian particles; n = 48, 40, 23, 38524, respectively. (b–c) The variance of $\cos\theta$ (b), n = 21, 7, 7, 4950; and $\sin\theta$ (c), n = 20, 7, 7, 4949, respectively. Statistically significant differences were calculated using a two-sample test for variance, a two-sample t-test, and n.s. means no significant difference. Median ± 1.5 × interquartile rate (IQR).

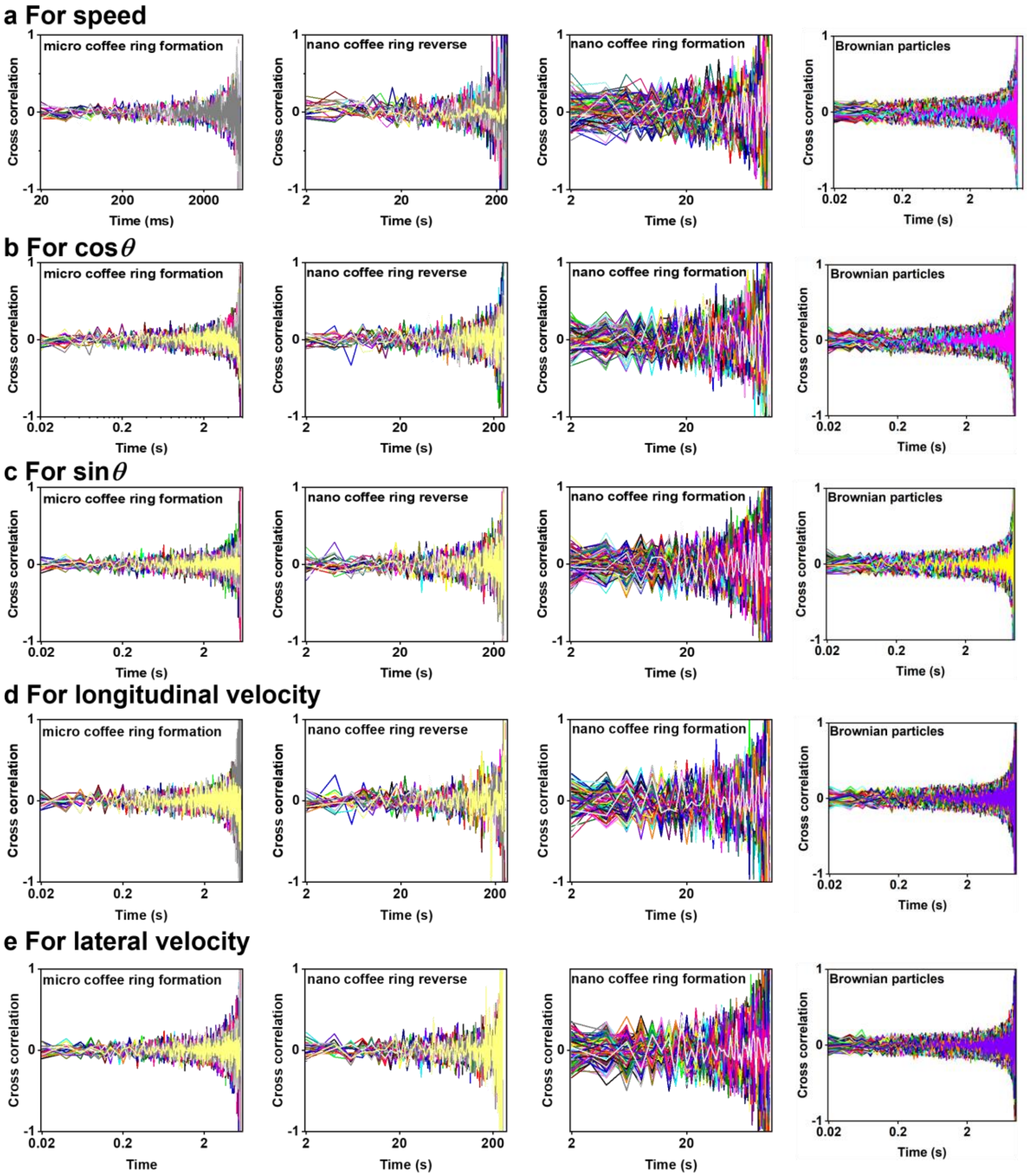

Fig. S36. Cross-correlation of (a) speed, (b) cosθ, (c) sinθ, (d) longitudinal velocity, and (e) lateral velocity for every two particles in micro coffee ring, reverse and formation of nano coffee ring, and Brownian particles, respectively. $n = 7, 7, 21, 100$ for each system.

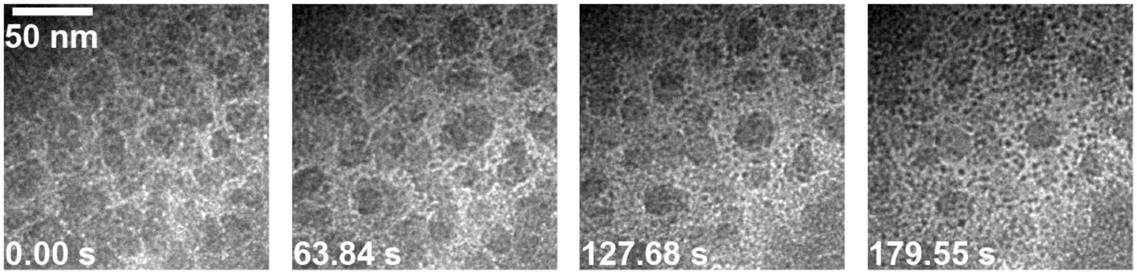

Fig. S37. When the particle concentration is too high, we fail to observe the nano coffee ring. Repulsive forces between nanoparticles may dominate over capillary flow. Experimental condition: F20, 200 kV, dose rate: 439 $e^{-}/(Å^2 \cdot s)$.

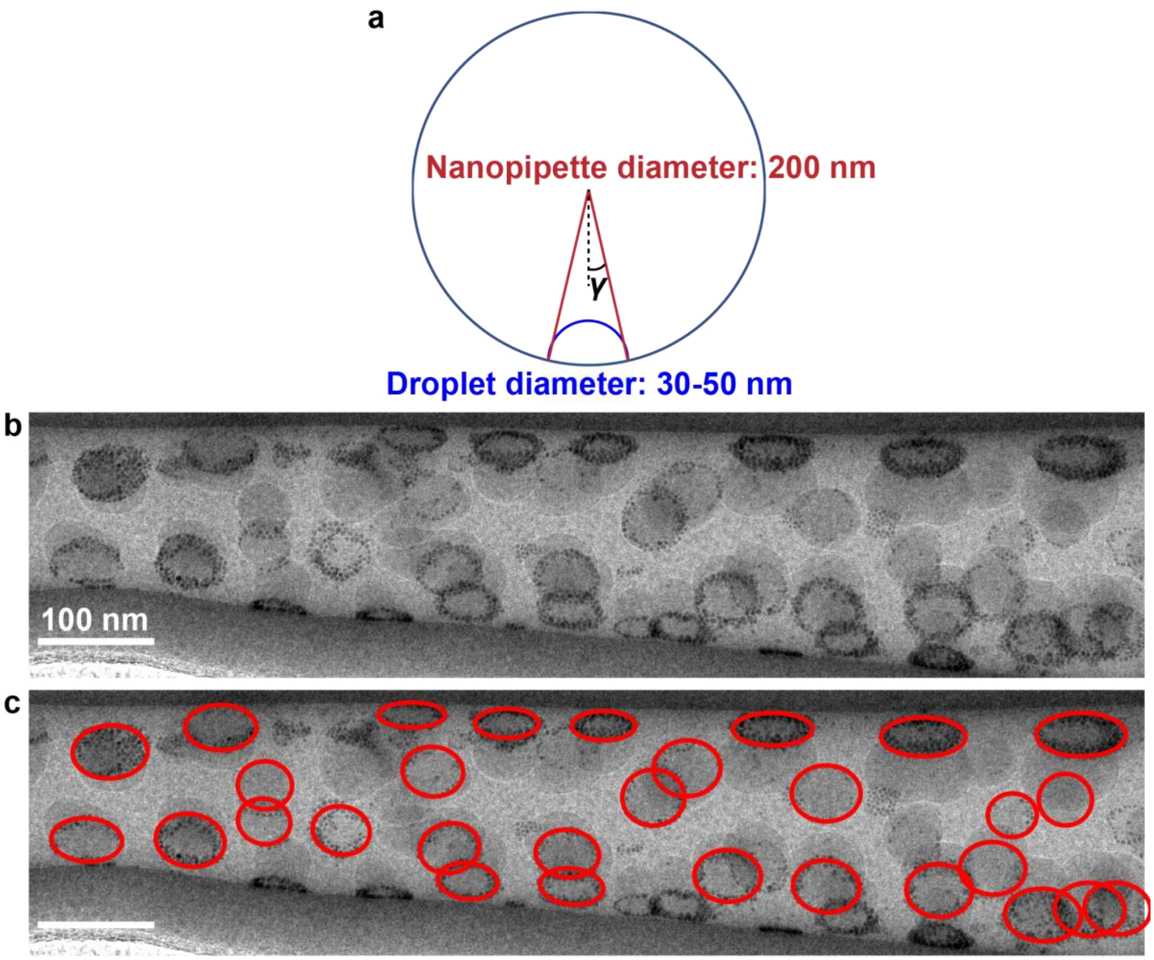

Fig. S38. Assessment of the effect of the nanopipette curvature (a) The droplet (blue) projects an angle of 9–14° to the center of the nanopipette's cross-section (navy). With such a small angle, the curvature effect on the contact angle is usually negligible. (b–c) A representative image capturing the 2D projections of nanodroplets. Shapes are slightly distorted at the edge but remain circular at the center of the nanopipette. Dose rate: 327 $e^{-}/(Å^{2}\cdot s)$.

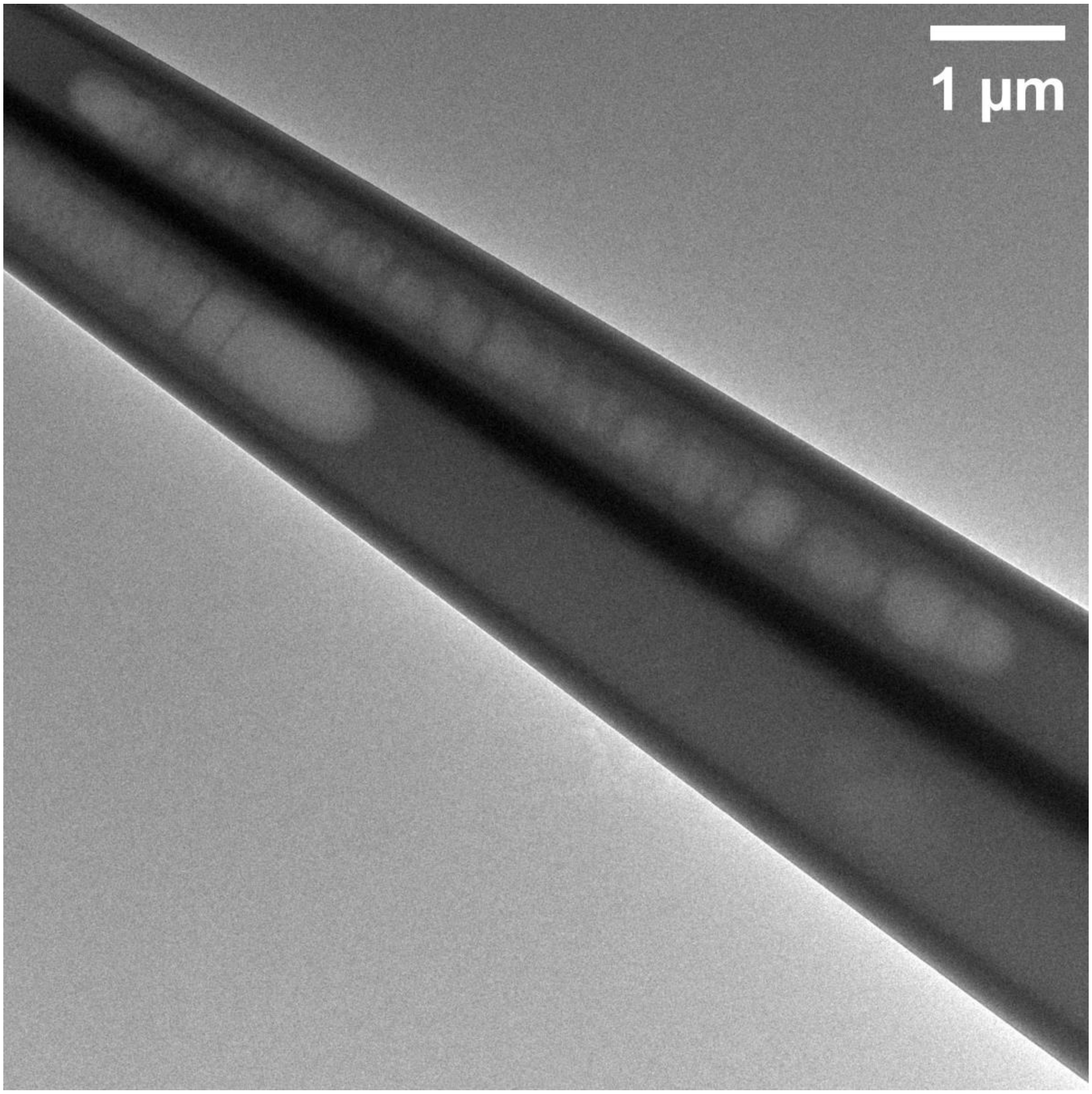

Fig. S39. Nanopipette with dual channel geometry. Dose rate: 1.3 $e^{-}/(Å^{2}\cdot s)$. To show the presence of liquids, we chose to show the bubbles.

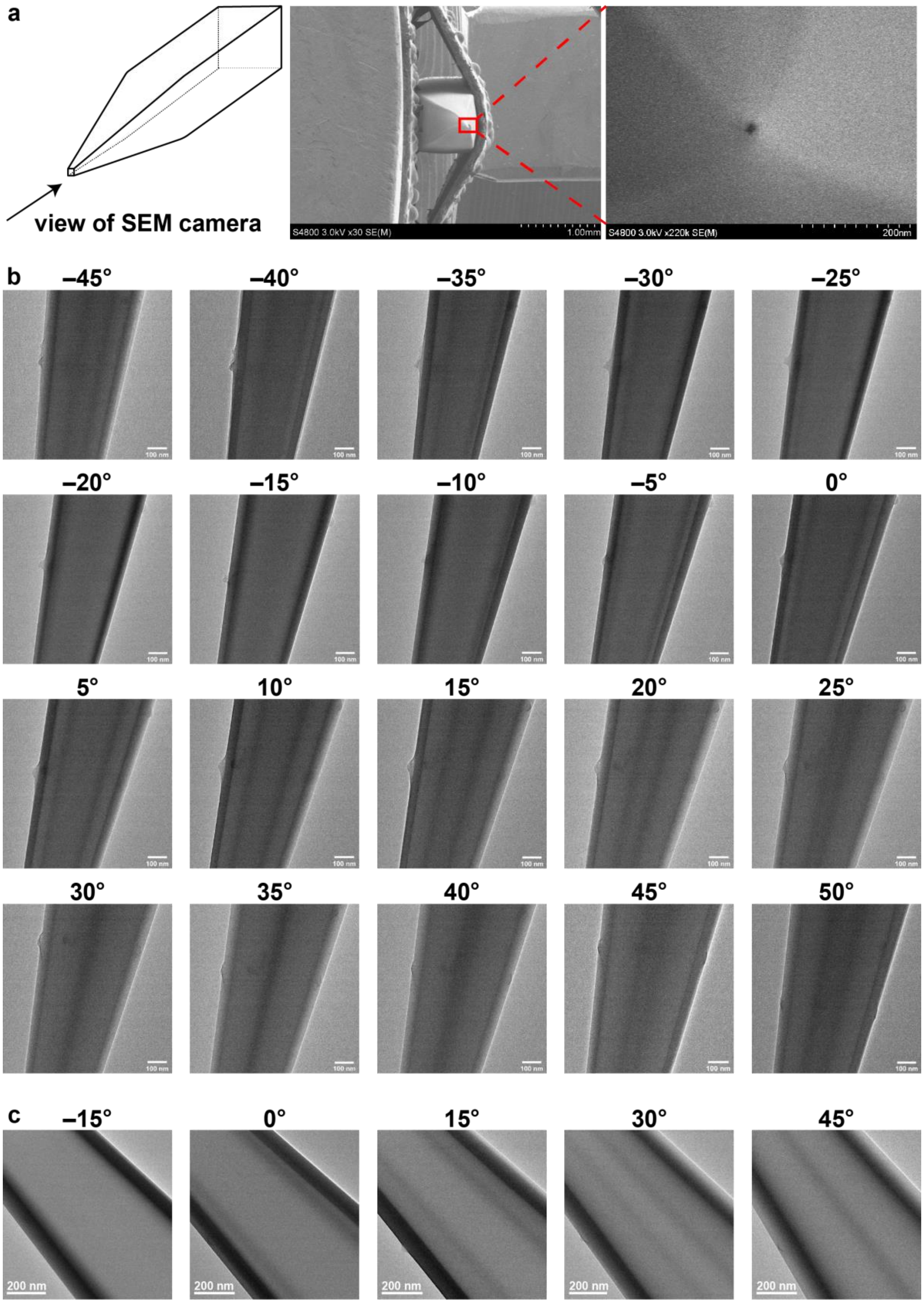
a
view of SEM camera
S4800 3.0kV x30 SE(M)
1.00mm
S4800 3.0kV x220k SE(M)
200nm
b
–45°
–40°
–35°
–30°
–25°
–20°
–15°
–10°
–5°
0°
5°
10°
15°
20°
25°
30°
35°
40°
45°
50°
c
–15°
0°
15°
30°
45°
200 nm

Fig. S40. (a) SEM images of quartz square nanopipette showing a near square-shaped tip. The shaft of the nanopipette is facing the camera. (b) TEM images of a quartz square nanopipette filled with oDCB show the positions of the four edges of the square (black lines) as the sample holder rotates. Dose rate: 2–4 $e^{-}/(Å^{2}\cdot s)$. (c) TEM images of a quartz square nanopipette without liquid show the positions of the four edges of the square rotating the sample holder. Dose rate: 1–2 $e^{-}/(Å^{2}\cdot s)$.

Table S1. Pulling parameters for quartz nanopipettes of different tip sizes (± SD) fabrication. Heat (0–999) specifies the output power of the laser and consequently the amount of energy supplied to the glass; Filament (0–15) specifies the scanning pattern of the laser beam that is used to supply Heat to the glass; The Velocity (0–255) parameter specifies the velocity at which the puller bar must be moving before the hard pull is executed; The Delay (0–255) parameter controls the timing of the start of the hard pull relative to the deactivation of the laser; The Pull parameter controls the force of the hard pull. See the Sutter Instrument P-2000 Operation Manual for more information.

Name	Parameters	Heat	Filament	Velocity	Delay	Pull	Tip diameter (nm)
1	line 1	835	5	30	128	90	$(4.0 \pm 0.8) \times 10^2$
	line 2	865	4	25	132	110	
2	line 1	835	5	30	128	90	76 ± 6
	line 2	865	4	25	132	140	
3	line 1	835	5	30	128	90	45 ± 6
	line 2	865	4	25	132	160	
4	line 1	835	5	30	128	90	20 ± 7
	line 2	865	4	25	132	195	

Table S2. Estimation of water lifetime (s) in the nanopipettes. Assume that the quartz nanopipettes are filled with liquid for 300 μm at the beginning.

α	20 nm	45 nm	76 nm	4.0×10^2 nm
0°	4.2×10^3	7.8×10^2	2.7×10^2	9.5
15°	5.4×10^3	1.0×10^3	3.4×10^2	12
30°	6.4×10^3	1.2×10^3	4.1×10^2	15
45°	7.3×10^3	1.4×10^3	4.7×10^2	17
60°	7.8×10^3	1.5×10^3	5.2×10^2	19
75°	8.1×10^3	1.6×10^3	5.5×10^2	20
90°	8.1×10^3	1.6×10^3	5.6×10^2	20

Table S3. Estimation of *o*DCB lifetime (s) in the nanopipettes. Assume that the quartz nanopipettes are filled with liquid for 300 μm at the beginning.

α	20 nm	45 nm	76 nm	4.0×10^2 nm
0°	4.7×10^4	7.8×10^3	2.6×10^3	86
15°	6.0×10^4	9.9×10^3	3.3×10^3	1.1×10^2
30°	7.1×10^4	1.2×10^4	3.9×10^3	1.3×10^2
45°	7.7×10^4	1.3×10^4	4.5×10^3	1.5×10^2
60°	8.0×10^4	1.4×10^4	4.8×10^3	1.7×10^2
75°	7.9×10^4	1.5×10^4	5.0×10^3	1.8×10^2
90°	7.4×10^4	1.4×10^4	5.0×10^3	1.8×10^2

Table S4. Estimation of oleyamine lifetime (s) in the nanopipettes. Assume that the quartz nanopipettes are filled with liquid for 300 μm at the beginning.

α	20 nm	45 nm	76 nm	4.0×10^2 nm
0°	2.6×10^9	3.2×10^8	9.7×10^7	2.9×10^6
15°	3.3×10^9	4.1×10^8	1.2×10^8	3.7×10^6
30°	3.7×10^9	4.8×10^8	1.5×10^8	4.5×10^6
45°	3.7×10^9	5.2×10^8	1.6×10^8	5.1×10^6
60°	3.5×10^9	5.3×10^8	1.7×10^8	5.6×10^6
75°	3.1×10^9	5.2×10^8	1.7×10^8	5.9×10^6
90°	2.5×10^9	4.9×10^8	1.7×10^8	6.0×10^6

Table S5. Summary of droplet evaporation experiments. A: FEI Tecnai F20; B: FEI Tecnai T20; C: JEM-2100 Plus HC; a: Pt precursor solution; b: Pt(acetylacetonate)$_2$ in oleylamine/DCM; c: oleylamine/*o*DCB; d: DCM; e: *o*DCB; i: open (Fig. 1E); ii: closed during irradiation (fig. S11, S20–21); iii: covered by vacuum grease (fig. S22a); iv: covered by big droplet (fig. S22b). '-' means no record.

Num.	TEM	Sol.	D_{tip} (nm)	Tip state	Liquid loss	Droplet formation	Dose rate ($e^{-}Å^{-2}s^{-1}$)
1	A	a	20	-	no	no	1
2	A	a	-	-	no	no	297-409
3	A	a	20	-	yes	yes	395
4	A	a	20	-	yes	yes	439
5	A	a	20	i	no	no	90
6	A	a	20-100	i	no	no	96
7	A	a	>250	i	no	no	97
8	A	a	237	i	no	no	246
9	A	a	104	i	no	no	-
10	A	a	100	i	no	no	201-435
11	A	a	20	i	Partially	no	90
12	A	a	20-40	i	Partially	no	90
13	A	a	100	i	yes	gel-like	183
14	A	a	20-100	i	yes	no	94-232
15	A	a	<100	i	yes	yes	150-900
16	A	a	20	ii	no	no	58-240
17	A	a	20	iii	no	no	671
18	A	a	105	iii	no	no	-
19	A	a	-	iii	no	no	-
20	A	a	20	iii	Partially	no	8
21	A	a	-	iv	no	no	198
22	A	a	-	iv	Partially	no	-
23	B	a	20	i	no	no	8
24	B	a	20	ii	no	no	96
25	B	a	20	ii	Partially	no	3
26	B	a	20	iii	no	no	6
27	B	a	20	iii	no	no	-
28	B	a	20	iii	no	no	-
29	B	c	20	i	yes	no	33
30	B	c	20	i	yes	no	62
31	B	c	20	ii	yes	yes	46
32	C	a	-	-	no	no	64
33	C	a	20	-	no	no	25-182
34	C	a	20	-	no	no	36-125
35	C	a	100	i	no	no	4

Num.	TEM	Sol.	D_{tip} (nm)	Tip state	Liquid loss	Droplet formation	Dose rate ($e^{-}Å^{-2}s^{-1}$)
36	C	a	20	i	no	no	11
37	C	a	20	i	no	no	26
38	C	a	20	i	no	no	59
39	C	a	50	i	no	no	64
40	C	a	>250	i	no	no	94
41	C	a	20	i	no	no	103
42	C	a	60	i	no	no	106
43	C	a	20	i	no	no	112
44	C	a	20-100	i	no	no	112
45	C	a	20	i	no	no	134
46	C	a	110	i	no	no	141
47	C	a	112	i	no	no	144
48	C	a	210	i	no	no	147
49	C	a	20	i	no	no	153
50	C	a	20-80	i	no	no	167
51	C	a	264	i	no	no	170
52	C	a	50	i	no	no	179
53	C	a	20	i	no	no	192
54	C	a	20-50	i	no	no	200
55	C	a	40	i	no	no	222
56	C	a	60	i	no	no	225
57	C	a	40	i	no	no	279
58	C	a	118	i	no	no	283
59	C	a	20	i	no	no	291
60	C	a	30	i	no	no	311
61	C	a	50	i	no	no	354
62	C	a	285	i	no	no	-
63	C	a	>250	i	no	no	0-809
64	C	a	20	i	no	no	100-452
65	C	a	20	i	no	no	101-127
66	C	a	60	i	no	no	272-306
67	C	a	25	i	no	no	2-809
68	C	a	40	i	no	no	35-1256
69	C	a	20	i	no	no	36-45
70	C	a	40	i	no	no	49-170
71	C	a	20	i	no	no	65-32
72	C	a	20	i	no	no	85-550
73	C	a	40	i	Partially	no	96
74	C	a	20	i	Partially	no	174
75	C	a	40	i	Partially	no	181

Num.	TEM	Sol.	D_{tip} (nm)	Tip state	Liquid loss	Droplet formation	Dose rate ($e^{-}Å^{-2}s^{-1}$)
76	C	a	20	i	Partially	no	201
77	C	a	20	i	Partially	no	256
78	C	a	20	i	Partially	no	4-209
79	C	a	40	i	Partially	no	78-705
80	C	a	30	ii	no	no	200
81	C	a	20	ii	no	no	224
82	C	a	20	ii	no	no	21-60
83	C	a	20	ii	Partially	no	164
84	C	a	20	ii	Partially	no	58-74
85	C	a	20	ii	yes	yes	38-480
86	C	a	40	iii	no	no	60
87	C	a	20	iii	no	no	-
88	C	a	20	iii	no	no	-
89	C	a	20	iii	no	no	-
90	C	a	20	iii	no	no	-
91	C	a	20	iii	no	no	-
92	C	a	30	iii	no	no	-
93	C	a	50	iii	no	no	-
94	C	a	50	iii	no	no	-
95	C	a	20	iii	no	no	19-200
96	C	a	20	iii	Partially	no	32
97	C	a	20	iv	no	no	635
98	C	b	-	-	Partially	no	96-119
99	C	b	-	-	yes	gel-like	66-230
100	C	b	20	i	no	no	67
101	C	b	20	i	no	no	205
102	C	b	54	i	no	no	-
103	C	b	50	i	no	no	26
104	C	b	30	i	no	no	27
105	C	b	20-150	i	yes	gel-like	12-86
106	C	b	20-45	i	yes	gel-like	60-258
107	C	b	-	ii	Partially	no	16
108	C	b	20	ii	Partially	no	20-154
109	C	b	20	ii	yes	gel-like	62-192
110	C	b	20	ii	yes	gel-like	59-79
111	C	b	20	ii	yes	gel-like	66-379
112	C	b	20	iv	no	no	51
113	C	b	-	iv	Partially	no	23-41
114	C	c	20	i	no	no	42-955
115	C	c	20	i	no	no	47-372

Num.	TEM	Sol.	D_{tip} (nm)	Tip state	Liquid loss	Droplet formation	Dose rate ($e^{-}Å^{-2}s^{-1}$)
116	C	d	-	-	no	no	166
117	C	e	20	-	no	no	5-113

Movie S1 (separate file). This movie shows GNRs experiencing diffusion at a wider part of the nanopipette. Each frame is 0.1594 s, played at 60 frames/s.

Movie S2 (separate file). This movie shows GNRs interacted to form tip-to-tip assembly in the aqueous solution with trajectories of individual particles. Each frame is 0.1594 s, played at 7 frames/s.

Movie S3 (separate file). This movie shows the electron-induced etching of Au nanoparticles. Each frame is 0.2 s, played at 5 frames/s.

Movie S4 (separate file). This movie shows the monomer attachment pathway of Pt nanoparticles' nucleation and growth process. Solution: *o*DCB/oleyamine platinum precursor solution. Each frame is 0.2 s, played at 30 frames/s.

Movie S5 (separate file). This movie shows the nanoparticle coalescence process occurring at the contact line (a) with that in evaporating solvent (b), with trajectories of individual particles tracked for comparisons. Solution: *o*DCB/oleyamine platinum precursor solution. Each frame is 0.2 s, played at 10 frames/s.

Movie S6 (separate file). This movie shows a GNR grew up to a GNA in the presence of CTAB. Each frame is 0.1594 s, played at 60 frames/s.

Movie S7 (separate file). This movie shows the dynamics and degradation of ssDNA in D_2O with contours of ssDNA. Solution: 5 μM ssDNA in D_2O. Each frame is 0.1594 s, played at 30 frames/s.

Movie S8 (separate file). This movie shows the liquid film becoming thin, dewetted and finally beaded on both sides of the inner wall. Solution: *o*DCB/oleyamine platinum precursor solution. Each frame is 1 s, played at 10 frames/s.

Movie S9 (separate file). This movie shows nanodroplets formed and the evaporation of a plain pinned droplet at 150 e/(Å2·s). The solution is an *o*DCB/oleyamine platinum precursor solution. Each frame is 0.2 s, played at 30 frames/s.

Movie S10 (separate file). This movie shows that the coffee ring effect occurs for firmly pinned nanodroplets, plain (a) or containing nanoparticle tracers (b), in an *o*DCB/oleyamine platinum precursor solution. Droplet diameters were plotted in real time for comparisons. Each frame is 0.2 s, played at 10 frames/s in (a) and 2 s, 10 frames/s in (b).

Movie S11 (separate file). This movie shows pinned droplet evaporation with nanoparticles and “stick-slip” droplets with and without particles at 900 $e^-/(Å^2·s)$. Solution: *o*DCB/oleyamine platinum precursor solution. Each frame is 0.2 s, played at 30 frames/s.

Movie S12 (separate file). This movie shows stick-slip dynamics occur for loosely pinned nanodroplets that reverse the nano coffee ring, plain (a) or containing nanoparticle tracers (b), in *o*DCB/oleyamine platinum precursor solution. Droplet diameters were plotted in real time for comparisons. Each frame is 2 s, played at 10 frames/s. Plateaus indicate that the sticking stage

occurs at 808–824 s, 806–846 s, and 850–894 s, three times for the plain nanodroplet; 812–840 s, and 860–990 s, two times for the nanodroplet containing nanoparticles.

Movie S13 (separate file). This movie shows the high ratio of stick-slip to pinning at 450–550 $e^{-}/(Å^{2}\cdot s)$ with contours of nanodroplets. Solution: *o*DCB/oleyamine platinum precursor solution. Each frame is 0.797 s, played at 15 frames/s.

Movie S14 (separate file). This movie shows no evaporation at 21 $e^{-}/(Å^{2}\cdot s)$. Solution: *o*DCB/oleyamine platinum precursor solution. Each frame is 0.1594 s, played at 60 frames/s.

Movie S15 (separate file). This movie shows a full view of nanoparticle coalescence at the contact line. Solution: *o*DCB/oleyamine platinum precursor solution. Each frame is 0.2 s, played at 30 frames/s.

Movie S16 (separate file). This movie shows particles' directed motion by capillary flow with micro coffee ring forming. Solution: 0.65 μL 1.25% carboxylate-modified polystyrene latex beads were dissolved in 400 μL glycerol and 400 μL deionized water. Each frame is 0.02 s, played at 50 frames/s.

SI References

1. J. Lu *et al.*, Room-temperature grafting from synthesis of protein–polydisulfide conjugates via aggregation-induced polymerization. *J. Am. Chem. Soc.* **144**, 15709-15717 (2022).
2. Q. Wang *et al.*, Controlled growth and shape-directed self-assembly of gold nanoarrows. *Sci. Adv.* **3**, e1701183 (2017).
3. H. Zheng *et al.*, Observation of Single Colloidal Platinum Nanocrystal Growth Trajectories. *Science* **324**, 1309-1312 (2009).
4. J. M. Yuk *et al.*, High-resolution EM of colloidal nanocrystal growth using graphene liquid cells. *Science* **336**, 61-64 (2012).
5. S. Zhang, M. Li, B. Su, Y. Shao, Fabrication and use of nanopipettes in chemical analysis. *Annu. Rev. Anal. Chem.* **11**, 265-286 (2018).
6. H. Wang, B. Li, Y. J. Kim, O. H. Kwon, S. Granick, Intermediate states of molecular self-assembly from liquid-cell electron microscopy. *Proc. Natl. Acad. Sci. U. S. A.* **117**, 1283-1292 (2020).
7. J. C. Crocker, D. G. Grier, Methods of digital video microscopy for colloidal studies. *J. Colloid Interface Sci.* **179**, 298-310 (1996).
8. K. Koo, J. Park, S. Ji, S. Toleukhanova, J. M. Yuk, Liquid-flowing graphene chip-based high-resolution electron microscopy. *Adv. Mater.* **33**, 2005468 (2021).
9. H. Wang, Z. Xu, S. Mao, S. Granick, Experimental guidelines to image transient single-molecule events using graphene liquid cell electron microscopy. *ACS Nano* **16**, 18526-18537 (2022).
10. Q. Chen *et al.*, Interaction potentials of anisotropic nanocrystals from the trajectory sampling of particle motion using *in situ* liquid phase transmission electron microscopy. *ACS Cent. Sci.* **1**, 33-39 (2015).
11. N. M. Schneider *et al.*, Electron–water interactions and implications for liquid cell electron microscopy. *J. Phys. Chem. C* **118**, 22373-22382 (2014).
12. Y. Xiang *et al.*, Tuning the morphology of gold nanocrystals by switching the growth of {110} facets from restriction to preference. *J. Phys. Chem. C* **112**, 3203-3208 (2008).
13. Q. Zhang *et al.*, Intertwining roles of silver ions, surfactants, and reducing agents in gold nanorod overgrowth: Pathway switch between silver underpotential deposition and gold–silver codeposition. *Chem. Mater.* **28**, 2728-2741 (2016).
14. W. Wang, I. Erofeev, P. Nandi, H. Yan, U. Mirsaidov, Evolution of anisotropic arrow nanostructures during controlled overgrowth. *Adv. Funct. Mater.* **31**, 2008639 (2021).
15. F. Zeng *et al.*, Real-time imaging of sulfhydryl single-stranded DNA aggregation. *Commun. Chem.* **6**, 86 (2023).
16. S. Keskin *et al.*, Visualization of multimerization and self-assembly of DNA-functionalized gold nanoparticles using in-liquid transmission electron microscopy. *J. Phys. Chem. Lett.* **6**, 4487-4492 (2015).
17. N. Jiang, Note on in situ (scanning) transmission electron microscopy study of liquid samples. *Ultramicroscopy* **179**, 81-83 (2017).
18. H. Miyake, Y. Tanaka, T. Takada, Characteristic of charge accumulation in glass materials under electron beam irradiation. *IEEE Trans. Dielectr. Electr. Insul.* **14**, 520-528 (2007).
19. U. M. Mirsaidov, H. Zheng, D. Bhattacharya, Y. Casana, P. Matsudaira, Direct observation of stick-slip movements of water nanodroplets induced by an electron beam. *Proc. Natl. Acad. Sci. U. S. A.* **109**, 7187-7190 (2012).
20. T. J. Woehl, T. Prozorov, The mechanisms for nanoparticle surface diffusion and chain self-assembly determined from real-time nanoscale kinetics in liquid. *J. Phys. Chem. C* **119**, 21261-21269 (2015).
21. W. Chen, J. Koplik, I. Kretzschmar, Molecular dynamics simulations of the evaporation of particle-laden droplets. *Phys. Rev. E* **87**, 052404 (2013).
22. J. M. Grogan, N. M. Schneider, F. M. Ross, H. H. Bau, Bubble and pattern formation in liquid induced by an electron beam. *Nano Lett.* **14**, 359-364 (2014).
23. Y. Tomo, S. Nag, H. Takamatsu, Observation of interfacial instability of an ultrathin water film. *Phys. Rev. Lett.* **128**, 144502 (2022).

24. J.-Y. Li *et al.*, Modes of nanodroplet formation and growth on an ultrathin water film. *J. Phys. Chem. B*, 3732-3741 (2024).
25. W. J. Jasper, N. Anand, A generalized variational approach for predicting contact angles of sessile nano-droplets on both flat and curved surfaces. *J. Mol. Liq.* **281**, 196-203 (2019).
26. C. Lee, X. Wei, J. W. Kysar, J. Hone, Measurement of the elastic properties and intrinsic strength of monolayer graphene. *Science* **321**, 385-388 (2008).
27. L. Wang *et al.*, Stress distribution around Fe_5Si_3 and its effect on interface status and mechanical properties of Si_3N_4 ceramics. *J. Am. Ceram. Soc.* **101**, 856-864 (2017).
28. G. Guerrini, A. H. A. Lutey, S. N. Melkote, A. Fortunato, High throughput hybrid laser assisted machining of sintered reaction bonded silicon nitride. *J. Mater. Process. Technol.* **252**, 628-635 (2018).
29. W. H. Kao, Y. L. Su, J. H. Horng, K. X. Zhang, Effects of Ti–C:H coating and plasma nitriding treatment on tribological, electrochemical, and biocompatibility properties of AISI 316L. *J. Biomater. Appl.* **31**, 215-229 (2016).
30. C.-F. Han, Y.-Z. Guo, C.-J. Chung, C.-H. Shen, J.-F. Lin, Effects of SiO_2 film thickness and operating temperature on thermally-induced failures in through-silicon-via structures. *Microelectron. Reliab.* **83**, 1-13 (2018).
31. M. Dal Bó *et al.*, An estimate of quartz content and particle size in porcelain tiles from young's modulus measurements. *Ceram. Int.* **43**, 2233-2238 (2017).
32. A. Zamani Zakaria, K. Shelesh-Nezhad, Quantifying the particle size and interphase percolation effects on the elastic performance of semi-crystalline nanocomposites. *Comput. Mater. Sci.* **117**, 502-510 (2016).
33. N. Bhattarai, D. L. Woodall, J. E. Boercker, J. G. Tischler, T. H. Brintlinger, Controlling dissolution of PbTe nanoparticles in organic solvents during liquid cell transmission electron microscopy. *Nanoscale* **11**, 14573-14580 (2019).
34. J. M. Grogan, H. H. Bau, The nanoaquarium: A platform for in situ transmission electron microscopy in liquid media. *J. Microelectromech. Syst.* **19**, 885-894 (2010).
35. E. Jensen, A. Burrows, K. Mølhave, Monolithic chip system with a microfluidic channel for in situ electron microscopy of liquids. *Microsc. Microanal.* **20**, 445-451 (2014).
36. K. Tanimura, T. Tanaka, N. Itoh, Creation of quasistable lattice defects by electronic excitation in SiO_2. *Phys. Rev. Lett.* **51**, 423-426 (1983).
37. M. A. Stevens-Kalceff, Electron-irradiation-induced radiolytic oxygen generation and microsegregation in silicon dioxide polymorphs. *Phys. Rev. Lett.* **84**, 3137-3140 (2000).
38. P. Avouris, R. E. Walkup, Fundamental mechanisms of desorption and fragmentation induced by electronic transitions at surfaces. *Annu. Rev. Phys. Chem.* **40**, 173-206 (1989).
39. M. L. Knotek, Stimulated desorption. *Rep. Prog. Phys.* **47**, 1499-1561 (1984).
40. V. Jamali *et al.*, Anomalous nanoparticle surface diffusion in LCTEM is revealed by deep learning-assisted analysis. *Proc. Natl. Acad. Sci. U. S. A.* **118**, e2017616118 (2021).
